# Supplementary figures and images for: Sleep deprivation and sleep intensity exert distinct effects on cerebral vasomotion and brain pulsations driven by the respiratory and cardiac cycles
Source: PLoS Biol. 2025 Nov 20;23(11):e3003500. doi: 10.1371/journal.pbio.3003500 (PMC12633874; doi:10.1371/journal.pbio.3003500)

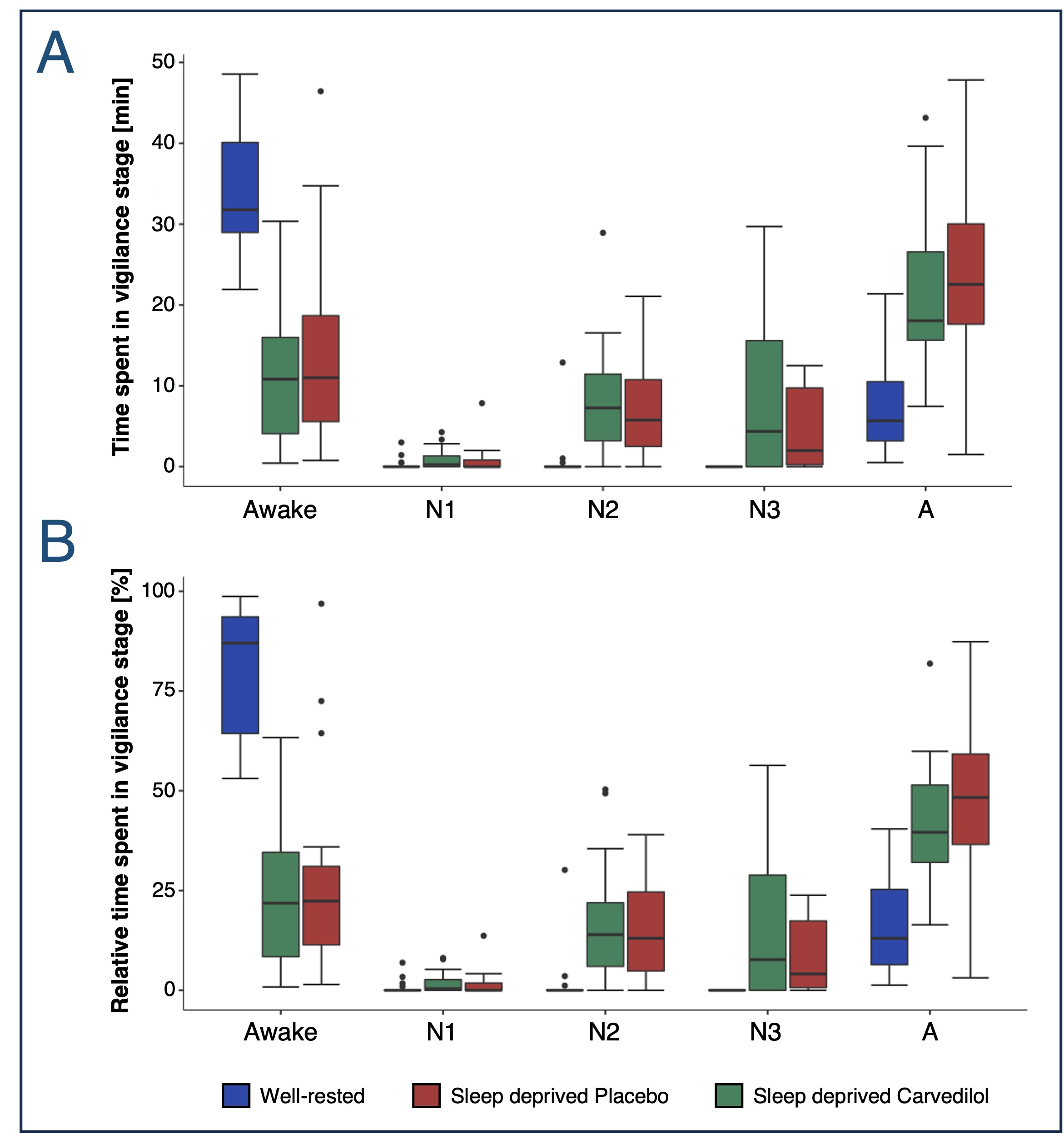

Supplement: S1 Fig — Boxplots illustrate (A) absolute and (B) relative time spent in wakefulness and NREM sleep stages N1, N2, and N3 during rested and sleep-deprived (placebo and carvedilol) scan sessions. All 30-sec EEG epochs recorded simultaneously with MR scans were included in analyses. EEG-epochs were included in analyses when the two independent EEG scorers agreed on staging. Epochs with scorer disagreement and epochs scored as artifacts were categorized as “A”. There is no difference between carvedilol and placebo conditions (Student’s paired t test, pall > 0.05). Box-plot elements include: median (center line), upper and lower quartiles (box limits), 1.5× interquartile range (whiskers), and outliers (points). N = 20. (TIFF) [file pbio.3003500.s001.tiff]

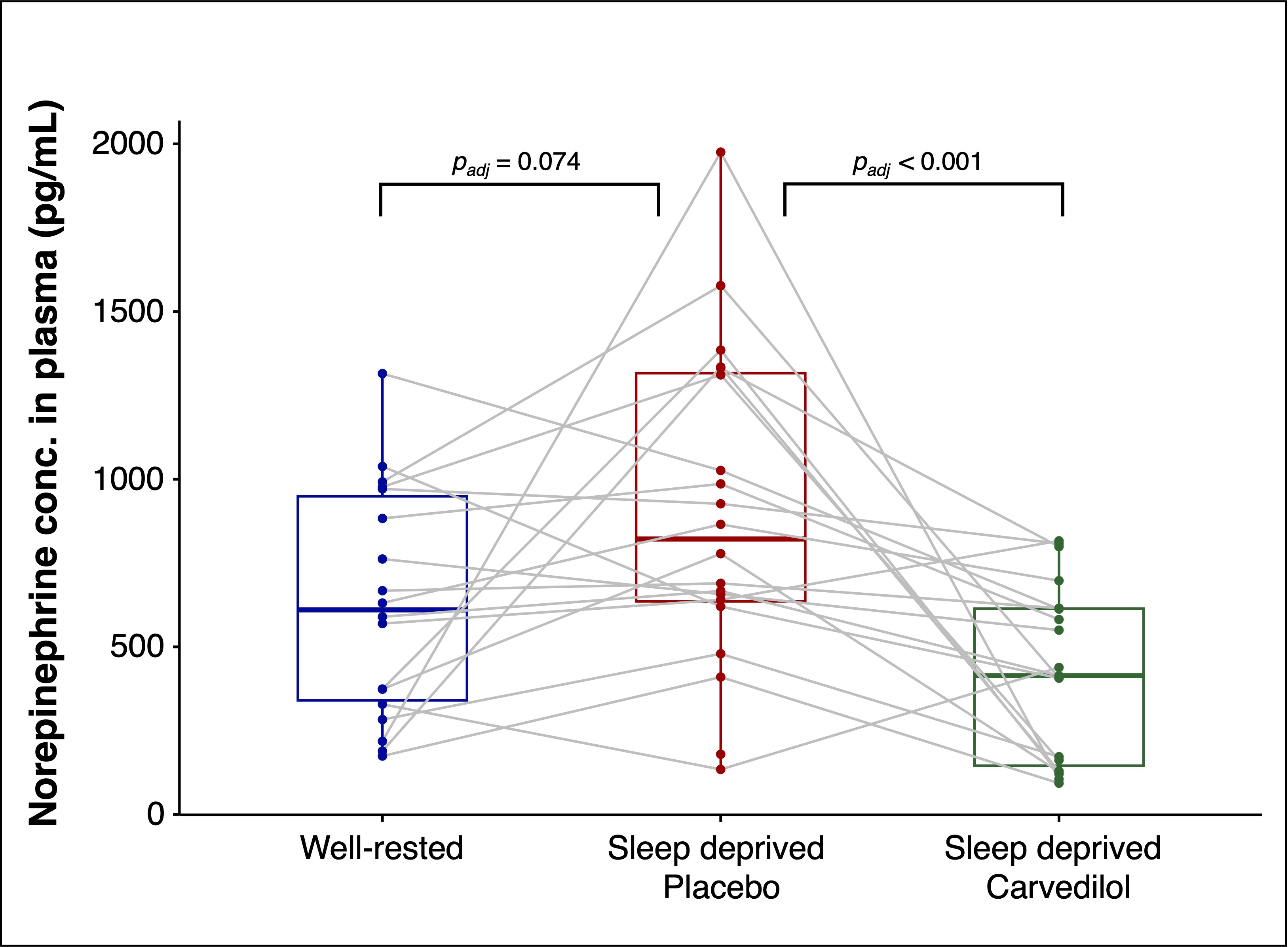

Supplement: S2 Fig — Normal range: 200–1,700 pg/mL. p-values are from a linear mixed model have been adjusted for multiple comparisons with Bonferroni correction Box-plot elements include: median (center line), upper and lower quartiles (box limits), and 1.5× interquartile range (whiskers). N = 20. (TIFF) [file pbio.3003500.s002.tiff]

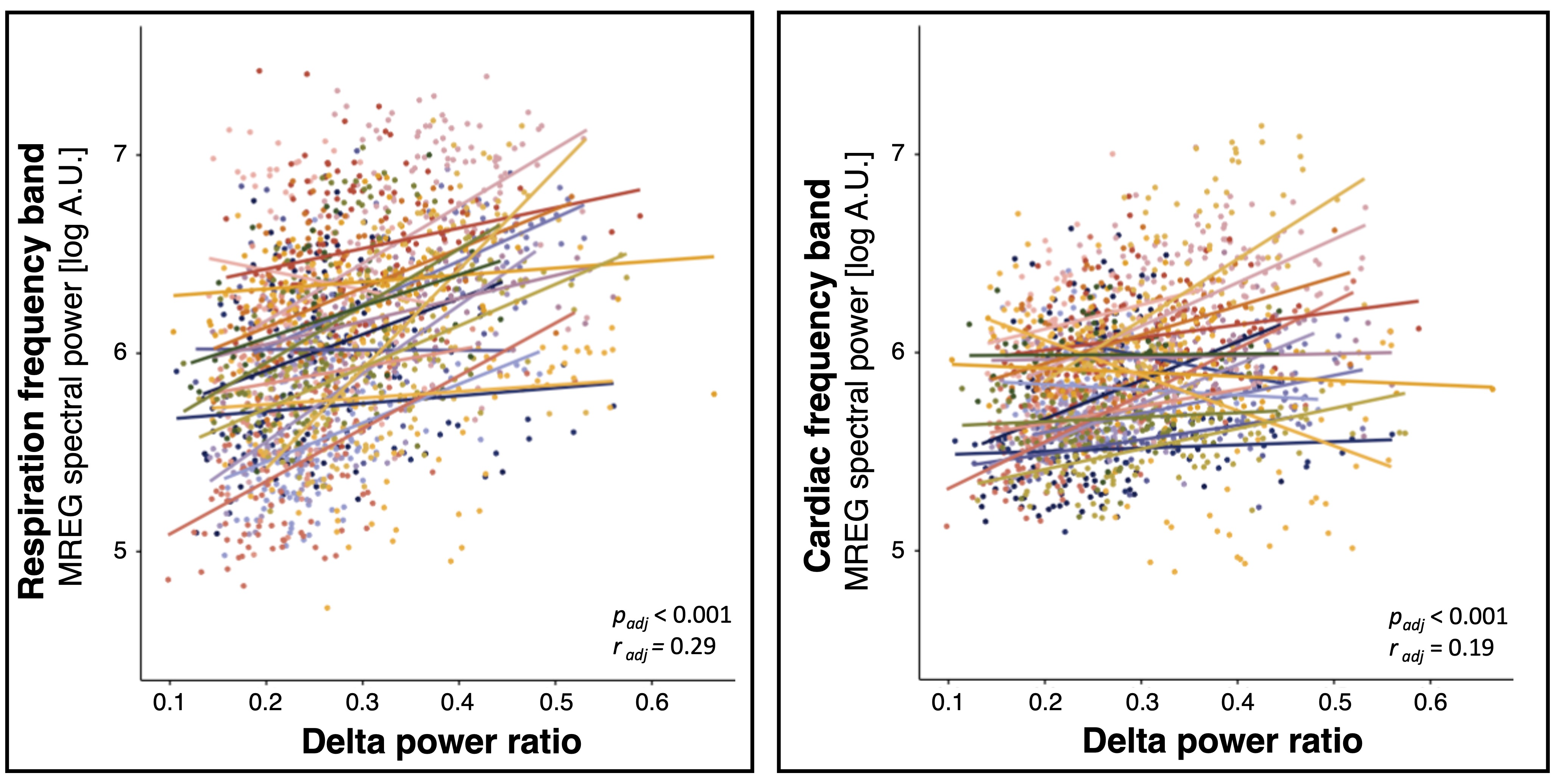

Supplement: S3 Fig — Each dot represents a 30-s epoch and each participant’s data is shown with a unique color. rest and pest are estimated correlation coefficient and estimated p-value from linear mixed models, where repeated measurements are taken into account. (A) A positive slope was observed for 17 of 19 participants in the respiration frequency band, and of these 13 were statistically significantly positively correlated. (B) For the cardiac band, 16 of 20 participants were positive and 11/20 had a significant positive slope. (TIFF) [file pbio.3003500.s003.tiff]

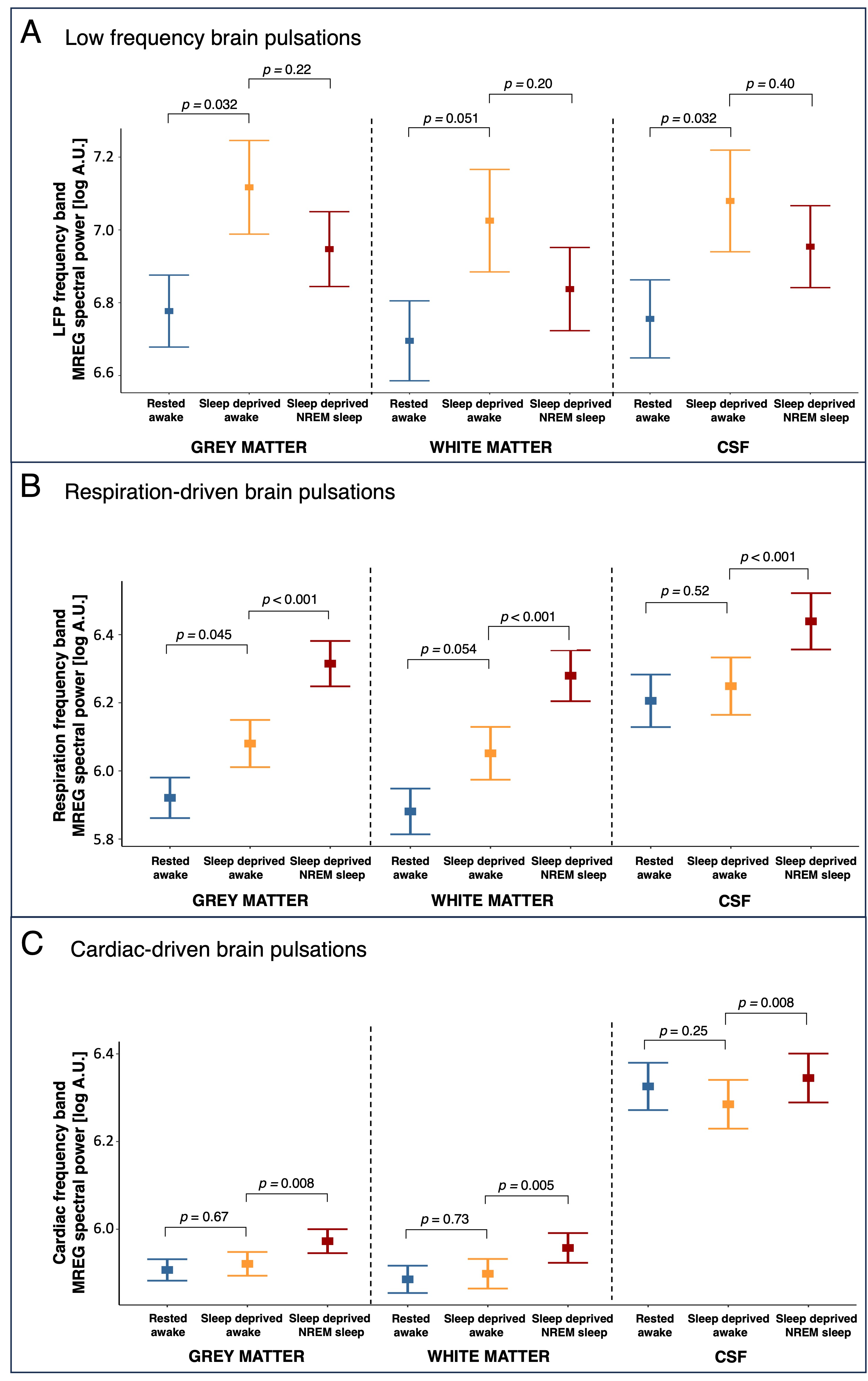

Supplement: S4 Fig — Results from sensitivity analyses of the effects of sleep deprivation and NREM sleep on MREG power in the (A) LFP, (B) respiration, and (C) cardiac frequency bands across three tissue types (gray matter, white matter and CSF). Error plots represent estimates (estimated means ± SEM) from linear mixed models, run separately for each tissue type. (TIFF) [file pbio.3003500.s004.tiff]
