# Supplementary material for: Sleep deprivation and sleep intensity exert distinct effects on cerebral vasomotion and brain pulsations driven by the respiratory and cardiac cycles
Source: PLoS Biol. 2025 Nov 20;23(11):e3003500. doi: 10.1371/journal.pbio.3003500 (PMC12633874; doi:10.1371/journal.pbio.3003500)
Supplement: S1 Table — (DOCX) [file pbio.3003500.s005.docx]

**S1 Table. Wakefulness period leading up to MR/EEG scan sessions.**

|  |  | **Sleep deprived scans** | | |
| --- | --- | --- | --- | --- |
|  | **Before**  **well-rested** | **Before**  **placebo** | **Before**  **carvedilol** | **Placebo vs carvedilol** |
| **Hours from wake-up-time to scan start (h)** | 11.1 ± 0.4 | 34.9 ± 0.3 | 34.8 ± 0.3 | *p* = 0.21 |
|  |  |  |  |  |
| **Prolonged wakefulness EEG-recording (h)** | - | 29.7 ± 3.2 | 28.5 ± 5.8 | *p* = 0.33 |
| **NREM sleep (min)** | - | 1.4 ± 5.2 | 0.8 ± 1.7 | *p* = 0.56 |
| **REM sleep (min)** | - | 0.0 ± 0.0 | 0.0 ± 0.0 | *NA* |

Time period participants spent awake between waking up from their 8-hours standardised sleep to the beginning of scan sessions. *p*-values represent results from students paired t-tests. All data are shown as mean ± SD. *N* = 20. NREM sleep: Non-rapid eye movement sleep stages N1-N3, REM sleep: Rapid eye movement sleep.
