# Supplementary material for: Sleep deprivation and sleep intensity exert distinct effects on cerebral vasomotion and brain pulsations driven by the respiratory and cardiac cycles
Source: PLoS Biol. 2025 Nov 20;23(11):e3003500. doi: 10.1371/journal.pbio.3003500 (PMC12633874; doi:10.1371/journal.pbio.3003500)
Supplement: S2 Table — (DOCX) [file pbio.3003500.s006.docx]

**S2 Table. Standardised sleep during nights before study days.**

|  | **Before**  **well-rested** | **Before  sleep deprived**  **placebo** | **Before  sleep deprived**  **carvedilol** | **Diff. between nights** |
| --- | --- | --- | --- | --- |
| **TIB (h)** | 7.9 ± 0.2 | 7.9 ± 0.3 | 7.9 ± 0.1 | *p* = 0.34 |
| **TST (h)** | 7.4 + 0.3 | 7.4 ± 0.3 | 7.4 ± 0.3 | *p* = 0.16 |
| **Sleep efficiency (%)** | 93.6 ± 3.4 | 94.4 ± 2.7 | 93.8 ± 3.5 | *p* = 0.25 |
| **NREM sleep (h)** | 5.5 ± 0.3 | 5.4 ± 0.4 | 5.4 ± 0.4 | *p =* 0.21 |

Data across the three EEG-monitored nights of 8-hr standardised sleep leading up to study days. Estimates and *p*-values are from a linear mixed model (hours of NREM sleep) and Friedman tests (TIB, TST & Sleep efficiency). All data are shown as mean ± SD. *N =* 20. TIB: time in bed, TST: Total sleep time, Sleep efficiency: % time spent asleep of time spent in bed (after lights are off), NREM sleep: duration of NREM sleep stages N1, N2 and N3.
