# Supplementary material for: Sleep deprivation and sleep intensity exert distinct effects on cerebral vasomotion and brain pulsations driven by the respiratory and cardiac cycles
Source: PLoS Biol. 2025 Nov 20;23(11):e3003500. doi: 10.1371/journal.pbio.3003500 (PMC12633874; doi:10.1371/journal.pbio.3003500)
Supplement: S3 Table — (DOCX) [file pbio.3003500.s007.docx]

**S3 Table. 30-sec dataset for evaluation of respiration- and cardiac-driven brain pulsations.**

|  |  | **Placebo** | | **Carvedilol** | |
| --- | --- | --- | --- | --- | --- |
|  | **Well-rested**  **wakefulness** | **Sleep deprived wakefulness** | **Sleep deprived**  **sleep (N2&N3)** | **Sleep deprived wakefulness** | **Sleep deprived sleep (N2&N3)** |
| **Participants in analysis (N)** | 20 | 17 | 17 | 15 | 19 |
| **Included 30-sec epochs (n)** | 35.6 [28.1, 43.0] | 10.8 [5.2, 16.3] | 28.7 [20.2, 37.2] | 9.5 [4.7, 14.4] | 33.8 [25.6, 42.1] |
| **Respiration rate (min^-1^)** | 15.2 [14.1, 16.4] | 16.2 [14.9, 17.7] | 14.9 [13.7, 16.2] | 16.2 [14.8, 17.7] | 14.3 [13.2, 15.5] |
| **Heart rate (min^-1^)** | 60.9 [57.5, 64.4] | 59.5 [56.1, 63.1] | 53.8 [50.8, 57.1] | 59.6 [56.2, 63.2] | 54.8 [51.7, 58.0] |
| **MAP (mmHg)** | 91.3 [89.4, 93.3] | 92.5 [90.1, 94.9] | | 88.2 [85.8, 90.5] | |
| **Pulse pressure (mmHg)** | 45.7 [42.4, 48.9] | 45.2 [42.1, 48.2] | | 44.3 [40.7, 47.9] | |

Data included in 30-sec dataset for analyses of sleep deprivation- and NREM sleep effects on spectral power in respiration and cardiac frequency bands. Values are shown as mean and 95% confidence intervals and are determined from linear mixed models to account for interindividual variance. Participants in analysis: Number of participants included for analysis in each condition with at least one 30-sec MREG-epoch with EEG-confirmed vigilance state (see methods). Respiration and heart rates: Physiological measurements recorded simultaneously with MREG. Respiration and heart rates are estimated for all 30-sec MREG epochs and used to determine epoch-wise respiration and cardiac frequency bands. MAP (mean arterial blood pressure = 1/3 x diastolic blood pressure + 2/3 x systolic blood pressure) and pulse pressure (difference between systolic and diastolic blood pressure) are averages of blood pressure measurements performed immediately before and after scans.
