# Supplementary material for: Sleep deprivation and sleep intensity exert distinct effects on cerebral vasomotion and brain pulsations driven by the respiratory and cardiac cycles
Source: PLoS Biol. 2025 Nov 20;23(11):e3003500. doi: 10.1371/journal.pbio.3003500 (PMC12633874; doi:10.1371/journal.pbio.3003500)
Supplement: S4 Table — (DOCX) [file pbio.3003500.s008.docx]

**S4 Table. 5-min dataset for evaluation of LFPs.**

|  |  | **Placebo** | | **Carvedilol** | |
| --- | --- | --- | --- | --- | --- |
|  | **Well-rested**  **wakefulness** | **Sleep deprived wakefulness** | **Sleep deprived**  **sleep (N2&N3)** | **Sleep deprived wakefulness** | **Sleep deprived sleep (N2&N3)** |
| **Participants in analysis (N)** | 19 | 12 | 14 | 12 | 17 |
| **Included 5-min scans (n)** | 1.6 [1.3, 1.8] | 1.1 [0.9, 1.3] | 3.1 [2.1, 4.1] | 1.3 [0.7, 1.8] | 3.3 [2.5, 4.1] |

Data included in 5-min dataset for analyses of sleep deprivation and NREM sleep effects on spectral power in the LFP frequency band (0.012 - 0.034 Hz). Values are shown as mean and 95% confidence intervals and are determined from linear mixed models to account for interindividual variance. Participants in analysis: Number of participants included in analysis in each condition, with at least one 5-min MREG scan with 80% (8/10 epochs) EEG-confirmed vigilance state (see methods).
