# Supplementary material for: Sleep deprivation and sleep intensity exert distinct effects on cerebral vasomotion and brain pulsations driven by the respiratory and cardiac cycles
Source: PLoS Biol. 2025 Nov 20;23(11):e3003500. doi: 10.1371/journal.pbio.3003500 (PMC12633874; doi:10.1371/journal.pbio.3003500)
Supplement: S5 Table — (DOCX) [file pbio.3003500.s009.docx]

**S5 Table. 30-sec dataset for evaluation of sleep depth effects.**

|  | **Placebo** | | **Carvedilol** | |
| --- | --- | --- | --- | --- |
|  | **N2 sleep** | **N3 sleep** | **N2 sleep** | **N3 sleep** |
| **Participants in analysis (N)** | 16 | 14 | 17 | 12 |
| **Included 30-sec epochs (n)** | 14.5 [9.1, 19.9] | 8.1 [4.5, 11.7] | 14.6 [8.9, 20.3] | 17.8 [7.1, 28.6] |
| **Respiration rate (min^-1^)** | 14.5 [13.4, 15.6] | 14.4 [13.3, 15.6] | 14.2 [13.4, 15.3] | 13.6 [12.6, 14.7] |
| **Heart rate (min^-1^)** | 52.3 [49.5, 55.2] | 53.9 [51.0, 56.9] | 54.6 [51.8, 57.6] | 54.5 [51.6, 57.5] |

Data included in 30-sec data set for sleep depth analyses. Values are shown as mean and 95% confidence intervals and are determined from linear mixed models to account for interindividual variance. Participants included in analysis: Number of participants in each condition who had at least one 30-sec epoch, where EEG-scorers agreed on either N2 or N3 NREM sleep. Respiration and heart rates were recorded simultaneously with MREG and estimated for all 30-sec MREG-epochs and subsequently used to determine epoch-wise respiration and cardiac spectral power.
