# Supplementary material for: Sleep deprivation and sleep intensity exert distinct effects on cerebral vasomotion and brain pulsations driven by the respiratory and cardiac cycles
Source: PLoS Biol. 2025 Nov 20;23(11):e3003500. doi: 10.1371/journal.pbio.3003500 (PMC12633874; doi:10.1371/journal.pbio.3003500)
Supplement: S6 Table — (DOCX) [file pbio.3003500.s010.docx]

**S6 Table. Recovery nights after sleep deprivation.**

|  | **Recovery sleep** | |  |
| --- | --- | --- | --- |
|  | **Placebo** | **Carvedilol** | **Treatment**  **effect** |
| **TIB: Time in bed (h)** | 8.0 ± 0.0 | 7.9 ± 0.3 | *p* = 0.37^⊥^ |
| **TST: total sleep time (h)** | 7.8 ± 0.1 | 7.7 ± 0.4 | *p* = 0.39^⊥^ |
| **Sleep efficiency (%)** | 97.3 ± 1.5 | 97.1 ± 1.6 | *p* = 0.60^⊥^ |
| **Sleep latency (h)** | 0.1 ± 0.1 | 0.1 ± 0.1 | *p* = 0.71^⊥^ |
| **REM latency (h)** | 1.4 ± 0.8 | 1.2 ± 0.7 | *p* = 0.27 |
| **NREM total (%)** | 70.6 ± 3.8 | 70.9 ± 3.5 | *p* = 0.51 |
| **REM total (%)** | 26.8 ± 3.9 | 26.3 ± 3.6 | *p* = 0.38 |
| **Stage N1 (%)** | 3.5 ± 2.3 | 2.6 ± 1.2 | *p* = 0.03^⊥^ |
| **Stage N2 (%)** | 37.4 ± 5.9 | 39.4 ± 7.0 | *p* = 0.10 |
| **Stage N3 (%)** | 29.7 ± 7.2 | 28.9 ± 8.4 | *p* = 0.76 |
| **WASO (%)** | 1.4 ± 0.9 | 1.7 ± 1.2 | *p* = 0.31 |

Characteristics of EEG-recorded recovery sleep after sleep deprivation in each treatment arm. Analyses were restricted to the first 8 hours (480 minutes). Sleep stages are presented as percentages of total time in bed. All data are shown as mean ± SD. *p*-values are from paired students t-tests or Wilcoxon signed rank tests (denoted ⊥). *N* = 20. TIB: time in bed, TST: total sleep time, Sleep latency and REM latency: time from lights-off to the first occurrence of stage N2 sleep, WASO: wakefulness after sleep onset.
