# Supplementary material for: Sleep deprivation and sleep intensity exert distinct effects on cerebral vasomotion and brain pulsations driven by the respiratory and cardiac cycles
Source: PLoS Biol. 2025 Nov 20;23(11):e3003500. doi: 10.1371/journal.pbio.3003500 (PMC12633874; doi:10.1371/journal.pbio.3003500)
Supplement: S1 File — (DOCX) [file pbio.3003500.s011.docx]

Supplementary Materials

**Statistical models for all reported results**

# *(i)* *Study cohort and descriptives:*

Paired two-tailed Student’s t-tests were used to compare the mean time intervals from wake-up time to scan start and the mean time spent in NREM/REM sleep during the periods of prolonged wakefulness prior to scans (S1 Table), as well as the relative time spent in wakefulness/sleep during MR-scans carvedilol and placebo conditions (S1 Fig) between. Paired t-tests were also used to evaluate the difference in reaction time and lapses of attention between rested wakefulness and sleep deprived wakefulness. *N* = 20.

Sleep during the standardised nights leading up to the three study sessions was compared by evaluating PSG-recorded sleep patterns across the nights (S2 Table). A linear mixed model including night type (before-well-rested vs before-placebo vs before-carvedilol) as a fixed effect and subject ID and study week (week 1-3) as random intercepts (study week being nested into subject ID), was used to evaluate mean differences in ‘Hours of NREM sleep (h)’. P-value for testing the fixed effect was obtained using a Wald test. Friedman tests were used to evaluate differences in ‘Time in bed (h)’, ‘Total sleep time (h)’ and ‘sleep efficiency (%)’ between the three nights, as these measures were not normally distributed. *N* = 20.

Effect of sleep deprivation and NREM sleep on EEG delta power ratio as well as on respiration- and heart rates (recorded simultaneously with MREG) were assessed in 30sec epochs from well-rested (awake scans) and sleep deprived placebo (awake and sleep scans) conditions (S3 Table) using linear mixed models. Sleep deprivation (well-rested vs. sleep-deprived) and vigilance state (awake vs NREM sleep) were included as additive fixed effects and Subject ID, period effect (well-rested scan, sleep-deprived scan 1, sleep-deprived scan 2) and scan type (lights on, lights off) were included as random intercepts with scan type being nested into period effect itself nested into subject ID. This linear mixed model will be referred to as LMM-*ii*. P-values for testing fixed effects were obtained using Wald tests. *N =* 20.

***(ii) Sleep deprivation enhances spectral power in the LFP frequency band:***

Effects of sleep deprivation and NREM sleep on whole-brain LFPs were evaluated in 5 min MREG scans from well-rested (awake scans) and sleep deprived placebo (awake and NREM sleep scans) conditions. Whole-brain spectral power within the LFP band was calculated by summing the spectral power of all bins included in the 0.01221 – 0.03418 Hz frequency range, after which it was log transformed to mitigate skewness in the distribution before being analysed. A linear mixed model with same fixed and random effect structure as LMM-*i* was used for analysis and P-values for testing fixed effects were obtained using Wald tests. The expected log spectral power at various conditions (e.g. sleep-deprived wakefulness) was computed from the mixed model estimates to illustrate the model fit (Fig 3B and S4A Fig). *N* = 20 (number of participants with data in each of the three conditions can be seen in Table S4).

The correlation between mean log spectral power in the LFP band (in either sleep deprived awake or NREM sleep scans) and PVT measurements (either pre-scan measurements or difference between pre- and post-scan measurements) was assessed considering two measurements per individual: one from placebo- and one from carvedilol conditions. A Wald test for the association between spectral power and PVT measures was obtained from a linear mixed model based on subject ID and adjusted for treatment and period effect nr (denoted *p*_adj_). The corresponding correlation coefficient (*r_adj_*) was evaluated (without proximation) as follows:

We introduce the following notations:

- $Y_{1,j}$ and $Y_{2,j}$ denote the spectral power at, respectively, placebo and carvedilol for individual $j$.
- $Z_{1,j}$ and $Z_{2,j}$ denote the PVT measurement at, respectively, placebo and carvedilol for individual $j$.
- $X_{j}$ denotes the covariates relative to individual $j$, here scan week.

We are interested the following correlation coefficient between spectral power and PVT measures:

$$r_{\mathrm{adj}}=cor\left( Y_{1,j},Z_{1,j}\left| X \right) \right)=cor\left( Y_{2,j},Z_{2,j}|X \right)$$

assumed to be independent of the treatment and scan week. We estimate this correlation under the following linear mixed model:

$$Y_{t,j}=\alpha_{t}+\beta X_{t,j}+u_{j}+\varepsilon_{t,j}$$

$$Z_{t,j}=\mu_{t}+\gamma X_{t,j}+v_{j}+\xi_{t,j}$$

Where $\left[ \begin{matrix} u_{j} \\ v_{j} \end{matrix} \right]\sim N\left( \left[ \begin{matrix} 0 \\ 0 \end{matrix} \right],\left[ \begin{matrix} \tau_{1} & \tau_{12} \\ \tau_{12} & \tau_{2} \end{matrix} \right] \right)$ and $\left[ \begin{matrix} \varepsilon_{t,j} \\ \xi_{t,j} \end{matrix} \right]\sim N\left( \left[ \begin{matrix} 0 \\ 0 \end{matrix} \right],\left[ \begin{matrix} {\sigma_{1}}^{2} & r_{\mathrm{adj}}\sigma_{1}\sigma_{2} \\ {r_{\mathrm{adj}}\sigma}_{1}\sigma_{2} & {\sigma_{2}}^{2} \end{matrix} \right] \right)$

using restricted maximum likelihood (REML).

For reference, Pearson correlation coefficients between mean log LFP spectral power and PVT measures were evaluated (regardless of subject ID, treatment and period effect nr) and denoted *r*_raw_ (Fig 4A, 4B, 4C and 4D). *N_awake_* = 16 and *N_sleep_* = 17.

For the sensitivity analysis conducted in a 2-min MREG dataset, the approach and linear mixed model were similar to those described for the 5-min dataset.

# *(iii) NREM sleep enhances spectral power in the respiration and cardiac frequency bands; N3 more so than N2 sleep:*

Effects of sleep deprivation and NREM sleep on whole-brain respiration- and cardiac-driven brain pulsations were evaluated in 30sec epochs from well-rested (awake scans) and sleep deprived placebo (awake and NREM sleep scans) conditions. Whole-brain spectral power within individually tailored respiratory- and cardiac frequency bands were log transformed to mitigate skewness in the distribution before being analysed using a linear mixed model with the same random effect structure as LMM-*i* and the same fixed effects with the addition of the base-10 logarithm of simultaneously recorded respiration or heart rates. *N* = 20 (number of participants with data in each of the three conditions can be seen in S3 Table).

Effects of sleep depth on spectral power within respiratory and cardiac frequency bands were evaluated in all 30sec epochs from the sleep deprived placebo condition classified as either wakefulness, N2 sleep or N3 sleep (*see* ‘MREG data analysis’). Analyses were performed as described above, but with sleep depth (sleep-deprived awake vs N2 vs N3) included as an additive fixed effect together with respiration or heart rates (log transformed). *N* = 19 (number of participants with data in each of the three conditions can be seen in S5 Table).

P-values for testing fixed effects in both of the above linear mixed models were obtained using Wald tests. The expected log spectral power at the various conditions (e.g. sleep-deprived wakefulness) was computed from the mixed model estimates under mean respiration or heart rates of all 30-sec epochs included in the respective analyses – and used to illustrate the model fit (Fig 3E and 3G; Fig 5B and 5D; S4B and S4C Fig).

Effects of vigilance and sleep stage on spectral power in the control frequency band (3.54 – 3.74 Hz) were evaluated as described above for the cardiorespiratory frequency bands.

**(*iv*) *EEG delta-power during MR-imaging correlates with spectral power in respiration and cardiac frequency bands:***

The association between log spectral power in individually tailored respiration- and cardiac frequency bands and simultaneously recorded delta power ratios was evaluated in all 30-sec epochs from well-rested and sleep deprived placebo scans, regardless of their visually scored vigilance and/or sleep states (*see* ‘Quantitative analyses of EEG during MRI’*)*.

We performed a linear mixed model with the same random effect structure as LMM-*i*, but with EEG delta power ratio and respiration or heart rates (log transformed) included as fixed effects. The reported p-value (denoted *p*_adj_) is the one of the delta power ratio in the mixed model.

The corresponding correlation coefficient (*r*_adj_) was deduced from the mixed model estimates using an approximation suggested by Lipsitz et al. [1] : $r = \beta/\sqrt{\beta^{2} + {\mathrm{df}\sigma}_{\hat{\beta}}^{2}}$, where $\beta$ denotes the delta power ratio parameter in the mixed model, $\sigma_{\hat{\beta}}$ its standard error, and $df$ its degree of freedoms. For reference, Pearson correlation coefficients were evaluated (regardless of subject ID) and denoted *r*_raw_. For reference, Pearson correlation coefficients between delta power ratio and log spectral power in all included 30-sec epochs were evaluated (regardless of subject ID, respiration and heart rates, period effect and scan type) and denoted *r*_raw_ (Fig 4F, 4G and S4 Fig). *N* = 20.

***(v) NREM sleep primarily enhances spectral power in respiration and cardiac frequency bands in grey and white matter:***

To explore whether the strength of brain pulsations differed between tissue types, we assessed spectral power within LFP, cardiac and respiratory frequency bands in grey matter, white matter and CSF. Data from well-rested (awake) and sleep deprived placebo (awake and NREM sleep) conditions were included in analysis and a linear mixed model with the same random effect structure as LMM-*ii* and the same fixed effects with the addition of tissue type (GM vs WM vs CSF), respiration or heart rates (log transformed; only for resp/card frequency bands) and the interaction between vigilance state and tissue type. P-values and estimates to illustrate the model fits (Fig 6C) were calculated as described in ***(ii)*** and ***(iii).*** *N* = 20 (number of participants with data in each of the three conditions can be seen in S3 and S4 Tables).

***(vi) Adrenergic antagonism decreases spectral power in LFP and cardiac frequency bands***:

Effects of carvedilol (randomised to sleep-deprived session 1 or sleep-deprived session 2) were assessed as follows: Paired two-tailed Student’s t-tests were used to compare blood pressure (S3 Table) and sleep characteristics during recovery sleep (REM latency, NREM total, REM total, Stage N2, stage N3; S4 Table) between placebo and carvedilol conditions. For recovery sleep characteristics not normally distributed (TIB, TST, Sleep efficiency, Sleep latency, Stage N1; Supplementary Table 4), Wilcoxon signed rank tests were used to compare the two conditions. *N* = 20. Plasma NE levels measured post-scan were evaluated using a linear mixed model with condition (well-rested vs placebo vs carvedilol) as a fixed effect and subject ID as random effect, with Bonferroni-adjusted p-values (S2 Fig). *N* = 20. Linear mixed models were also used to evaluate the effect of treatment on whole-brain log spectral power within the LFP, respiration and cardiac frequency bands as well as on respiration and heart rates. In these models, vigilance state (awake vs NREM sleep), treatment (placebo vs carvedilol), respiration and heart rates (log transformed; only included for resp./card. frequency bands) and the interaction between vigilance state and treatment were included as additive fixed effects, while the random effect structure was similar to LMM-*i*. P-values and estimates to illustrate the model fits (Fig 7) were calculated as described in ***(ii)*** and ***(iii).*** *N* = 20 (number of participants with data in each of the three conditions can be seen in S3 and S4 Tables).

**Power calculation**

The sample size for the study was based on data from Xie et al. [2], which suggest a within-subject Cohen’s dz of 2.2 for brain fluid flow differences between sleep and wakefulness, and 0.9 for the effect of adrenergic inhibition. With an alpha level of 0.01 and 85% power, the estimated sample sizes were *N* = 7 and *N* = 20 for the two parts, respectively. Thus, a study cohort of *N* = 20 was chosen.

The Cohen’s dz found by Xie et al. is based on rodent data and may be too optimistic for our study as variability and measurement error are typically more pronounced in human studies. For that reason, we chose to apply an alpha level of 0.01 in the power calculation instead of the 0.05 level applied in the main statistical analyses.

**Sensitivity analysis in 2-min MREG dataset: Effect of sleep deprivation and NREM sleep on LFPs (0.02 – 0.034 Hz)**

We conducted a sensitivity analysis of the effect of sleep and sleep deprivation on LFPs using a 2-minute MREG dataset with stricter vigilance state classification than the primary 5-min dataset. Here, all four 30-second EEG epochs (100%) in each 2-minute interval were required to be classified as either wakefulness or NREM sleep (stages N2 or N3) by the two independent EEG scorers (see classification criteria in Methods). In addition, wakefulness scans had to occur during lights-on periods and sleep scans during lights-off periods, as was also the case for 5-min scans. Due to the shorter time window, data were then high-pass filtered at 0.02 Hz, and LFPs were analysed in a narrower frequency band (0.02–0.034 Hz) than in the 5-min intervals. Otherwise, spectral analysis and statistical procedures were performed as described in Methods for the 5-minute dataset.

Analysis of this 2-minute dataset (N_RestedAwake_= 19; N_SleepdeprivedAwake_= 12; N_N2N3_= 16) showed that sleep deprivation was associated with an increase LFP spectral power of 96% (p = 0.038), while NREM sleep led to a numerical, but non-significant, decrease in power compared to sleep-deprived wakefulness (p = 0.38). These findings confirm the main results observed in the 5-minute dataset.

**Effect of sleep deprivation and NREM sleep on LFPs in a 0.01 – 0.098 Hz frequency band in the 5-minute MREG dataset**

To assess the robustness of our main LFP findings, we conducted a sensitivity analysis on the same 5-min dataset used for primary analyses, applying a broader frequency band of 0.01–0.098 Hz instead of the originally chosen 0.01–0.034 Hz band (see S4 Table).

We found that sleep deprivation increased LFP spectral power in the 0.01–0.098 Hz band by 146% (p = 0.01), while NREM sleep led to a 101% reduction compared to the preceding period of sleep-deprived wakefulness (*p* = 0.02).

These results are thus consistent with those observed using the narrower B-wave band, but showed stronger effect sizes and lower p-values. We did not pursue additional analyses in this broader range, as a systematic comparison of LFP frequency bands is outside the scope of the present study.

**Supplementary References**

1. Lipsitz SR, Leong T, Ibrahim J, Lipshultz S. A Partial Correlation Coefficient and Coefficient of Determination for Multivariate Normal Repeated Measures Data. Journal of the Royal Statistical Society: Series D (The Statistician). 2001;50: 87–95. doi:10.1111/1467-9884.00263

2. Xie L, Kang H, Xu Q, Chen MJ, Liao Y, Thiyagarajan M, et al. Sleep drives metabolite clearance from the adult brain. Science. 2013;342: 373–377. doi:10.1126/science.1241224
