# Supplementary material for: Sleep deprivation and sleep intensity exert distinct effects on cerebral vasomotion and brain pulsations driven by the respiratory and cardiac cycles
Source: PLoS Biol. 2025 Nov 20;23(11):e3003500. doi: 10.1371/journal.pbio.3003500 (PMC12633874; doi:10.1371/journal.pbio.3003500)
Supplement: S2 File — (PDF) [file pbio.3003500.s012.pdf]

Effects of sleep deprivation and adrenergic inhibition on glymphatic flow in humans

Experimental protocol

Research supervisor

Gitte Moos Knudsen, professor, M.D.

Other project members

Sebastian C. Holst, Ph. D.
Maiken Nedergaard, professor, M.D.
Poul Jennum, professor, M.D.

Collaborators

Klinik for Klinisk Fysiologi og Nuklearmedicin, PET og cyklotronenheden, and Radiologisk klinik, Rigshospitalet.
Dansk Center for Søvnmedicin, klinisk neurofysiologisk afdeling, Rigshospitalet.

Address

Neurobiology Research Unit, building 6931
Rigshospitalet
Blegdamsvej 9
2100 København Ø

Project description

The aim of this project is:

- To investigate the rate and extent of the glymphatic system in the human brain in both wakefulness and sleep.
- To examine the association between adrenergic inhibition, glymphatic flow and sleep intensity to assess causal relationships.
- To investigate whether the sleep dependent glymphatic flow, and its modulation by an adrenergic antagonist, predict changes in cognitive performance.

Scientific background

Sleep is a universal biological process critical to maintenance of life. Lack of or insufficient sleep, has been associated with a range of diseases including obesity, cardiovascular disease, reduced cognition, impaired learning, and increased risk of motor vehicle accidents (Landolt

et al., 2014). Sleep is also associated with pronounced alterations in the brain, including reduced firing of arousal maintaining monoaminergic neurons (Holst and Landolt, 2015).

Recently, a novel molecular function of sleep was described in rodents. A macroscopic pathway in the central nervous system, known as the glymphatic system, was revealed to be specifically activated during non-rapid eye movement sleep, enhanced by up to 90% when compared to wakefulness (Xie et al., 2013). The glymphatic system facilitates the clearance of interstitial waste products from neuronal metabolism, an effect that may explain how the brain, despite its high metabolic rate and the fragility of neurons to toxic waste products, lacks a conventional lymphatic system.

It has long been a mystery why astrocytes in the brain express the water channel aquaporin-4 (AQP4). Astrocytes encapsulate the brain vasculature and form a boundary to the perivascular space. The glymphatic system provide a possible explanation and suggests that the AQP4 channels may enable glymphatic flow into the brain parenchyma (Jessen et al., 2015). Intriguingly, it has been shown that adrenergic antagonists specifically enhance expression of AQP4 channels (Yasui et al., 1997) and that the inhibition of adrenergic receptors enhance glymphatic clearance (Xie et al., 2013), suggesting a causal relationship.

Because the sleep dependent effects of the glymphatic flow has so far only been described in rodent models, in this project, we aim to demonstrate the existence of the glymphatic system in humans. With newly developed magnetic resonance imaging (MRI) protocols such as ultra-fast magnetic resonance encephalography (MREG), the extent of the glymphatic system can now be investigated non-invasively in humans (Kiviniemi et al., 2016).

In order for us to quantify the change in glymphatic clearance between sleep and wakefulness, it is necessary to measure the glymphatic process in both vigilance states, requiring that volunteers nap in the MRI scanner. Moreover, to clarify causal relationships, we will challenge the glymphatic system via adrenergic inhibition. To do so, we will administer the α- and β- adrenergic antagonist carvedilol that readily crosses the blood-brain barrier (Stenehjem et al., 2009; Wang et al., 2011). The drug will be perorally administered before sleep in the MRI, in a double blind, placebo controlled manner. To assess sleep quality and function, cognitive testing will be performed before and after the nap in the MRI scanner. Moreover, to distinguish sleep and wakefulness, electroencephalic recordings (EEG) will be performed during MR-imaging. Because sleep is a strong homeostatic regulated process, sleep quality, duration and timing will be controlled by EEG monitoring, immediately prior to and during the study to ensure that data is intra- and inter-individually comparable.

What is fMRI and EEG?

Functional magnetic resonance imaging (fMRI) provides excellent spatio-temporal resolution, and allows for visualization of local changes in brain blood flow (arterial spin

labeling, ASL), activity (task based fMRI), connectivity (resting-state fMRI, rs-fMRI) and biochemical composition (MR spectroscopy, MRS). In addition, an ultra-fast magnetic resonance encephalography (MREG) method that allows for visualization of cerebral fluid flow was recently developed (Kiviniemi et al., 2016). The electroencephalography (EEG) on the other hand lacks high spatial resolution but possesses an outstanding temporal resolution. EEG is essential to distinguish between the three vigilance states of wakefulness, non-rapid eye movement (NREM) and rapid eye movement (REM) sleep. EEG slow wave activity (SWA) during NREM sleep, is the best and most well established marker of homeostatic sleep need (Achermann and Borbély, 2011). Both EEG and fMRI are non-invasive imaging modalities. The primary aim of applying fMRI and EEG simultaneously is to measure glymphatic flow in the brain while at the same time determining whether the subjects are asleep or awake.

What is the clinical relevance?

Humans spend roughly a third of their life asleep, yet the physiological benefit of sleep remains elusive. Current sleep pharmacology is almost solely symptomatic, either treating sleepiness or promoting sleep. This study aims to describe a physiological origin of why humans need sleep, which may lead the way for the development of drugs or cognitive therapies to enhance or promote sleep. Moreover, the here suggested manipulation of glymphatic clearance with the adrenergic antagonist carvedilol, and the assessment of corresponding improvements in cognitive performance, may propose a causal way to enhance sleep. This study may therefore, define entirely new approaches for enhancing sleep quality and reduce or delay the need for sleep without compromising cognitive function.

Hypotheses

Hypothesis 1: Sleep promotes cerebrospinal fluid pulsations (glymphatic flow) in the human brain. Challenging the sleep-homeostat by sleep deprivation promotes glymphatic flow further. The rate of glymphatic flow is expected to be proportional to simultaneously measured EEG slow wave activity in NREM sleep.

Hypothesis 2: Promoting sleep with the adrenergic antagonist carvedilol will enhance glymphatic clearance and sleep intensity, providing evidence for causality.

Hypothesis 3: Glymphatic flow is associated with cognitive performance levels following sleep. Enhanced glymphatic flow predicts cognitive performance improvements.

Study design and experimental methods

To test the hypotheses listed above, the study will enrol healthy volunteers. Enrolled subjects will go through a study protocol consisting of a polysomnographic screening night followed by cerebral EEG and MR-imaging sessions, before and after sleep deprivation. Adjacent to

each imaging session, subjects will perform a battery of cognitive tests and questionnaires. Sleep-deprivation and the sleep-deprived imaging sessions will be performed twice, in a placebo controlled, cross-over manner with the selective adrenergic antagonist carvedilol.

Recruitment

Subjects will be recruited from a locally established database (www.nru.dk) of potential volunteers who have expressed a specific interest in participating in a brain-research experiment. Furthermore, subjects will be recruited from www.forsøgspersoner.dk. The advertisement used for this website is attached. The database ensures that we can contact suitable subjects who likely fit our inclusion and exclusion criteria. Subjects who have previously participated in another study in the research unit and who have consented to be contacted again will also be approached. As a general rule and in accordance with Danish law, all participants will provide us with written informed consent to be enrolled in the study. This is further described in the section "*Guidelines for obtaining informed consent*."

In- and exclusion criteria

Inclusion criteria:

1. Healthy volunteer (male or female) between 18 and 35 years.
2. Good sleeper with sleep efficiency above 80%.

Exclusion criteria:

1. Current or former primary psychiatric disorder (DSM IV Axis I or WHO ICD-10 diagnostic classification).
2. Current or former primary psychiatric disorder (DSM IV Axis I or WHO ICD-10 diagnostic classification) among first degree relatives (parents or siblings).
3. Current or previous neurological disease, severe somatic disease, or the consumption of drugs likely to influence the test results.
4. Claustrophobia or fear of being in an MR-scanner.
5. Alcohol or drug abuse.
6. Regular smoking or nicotine addiction
7. Extreme morning or evening type, or extreme short or long sleeper.
8. Disordered sleep, regular shift-work or extreme tiredness (e.g. ESS > 10).
9. Crossing of multiple time zones within the last 6 months.
10. Extreme use of stimulants such as caffeine.
11. Not adhering to the prescribed sleep-wake schedule before study initiation.
12. Left handedness.
13. Obesity (BMI > 27.5).
14. Non- fluent in Danish or pronounced visual or auditory impairments.

15. Current or past learning disability.
16. Large head size (>59 cm in circumference).
17. Pregnancy. This is tested by a pregnancy test performed immediately prior to the baseline measurements.
18. Lactation.
19. Contraindications for MRI (pacemaker, metal implants, etc.).
20. Allergy to the ingredients in the administered drug.
21. Abnormal ECG (e.g. prolonged QT syndrome, etc.)
22. Dizzy when changing from supine to upright position (e.g. postural orthostatic tachycardia syndrome).
23. Mild hypotension (blood pressure below 100/70 mmHg) or hypertension (blood pressure above 140/90 mmHg).

General information about included volunteers

Healthy subjects are recruited as described above. We need 25 subjects to conduct the study (power calculations are given below) and another 20 subjects to perform and validate the neuropsychological testing. We will therefore recruit and screen up to 50 subjects in order to take account for potential drop-outs and excluded subjects. After confirmed inclusion, all volunteers will receive a personal plan with time and dates for examinations and tests. All volunteers will be requested not to drink alcohol immediately prior to or during the study. Moreover, the consumption of caffeine will be prohibited in the week before the study starts. This includes the daily consumption of >2 cups of coffee, black tee, or caffeinated soft drinks. During this week, subjects will be required to wear an activity monitor (accelerometer) to verify their adherence to a prescribed 16-hour wake, 8-hour sleep schedule. Before baseline measurements, and before both sleep deprivation sessions, subjects will be asked to provide a urine sample to screen for drug consumption and (for women) to do a pregnancy test. All urine samples are discarded immediately after the analysis.

Study design and randomization

The study design is illustrated in Figure 1 and can be divided into four consecutive steps: 1. Interviewing, 2. Screening, 3. Baseline and 4. Sleep deprivation/adrenergic challenge. A flow-chart illustrating these four steps is shown in Figure 2.

The study will generally be adapted to subject's habitual sleep time. As such, bed and wake times may vary, whereas the time between tests, drug administration and imaging is fixed.

1. Interviewing: During the interview, most inclusion and exclusion criteria will be verified and subjects are asked to fill out trait-related questionnaires related to handedness, chronotype and habitual sleep duration, etc. A medical history will also be acquired by

either a medical doctor, a nurse or a medical student under proper supervision, to ensure subjects are in good health and to perform blood pressure measurements.

2. Screening: To ensure that volunteers are good sleepers and physically healthy before study enrolment they will be equipped with an activity monitor (small accelerometer with the size of a watch) and undergo a polysomnographic screening night in a sleep clinic. Here potential ECG, sleep and breathing disorders will be diagnosed. Only subjects free of sleep and chronobiologic disorders, and with a high sleep efficiency, will be included.

3. Baseline: To assess the level of glymphatic clearance under standardized baseline conditions, healthy subjects will be enrolled. Following nocturnal adaptation and baseline 8-hour sleep recordings (~24:00 to ~8:00 o'clock, according to habitual sleep times), subjects will be prepared for the simultaneous EEG and fMRI experiment at ~16:00 o'clock, 8 hours after awakening. A battery of neuropsychological tests and questionnaires is performed ~2h prior (~14 o'clock), and ~30 min after (~19:00 o'clock) the EEG/fMRI session. The battery assesses e.g. visual attention (TVA), simple reaction time (psychomotor vigilance task), the Cambridge gambling task, the verbal affective memory test (VAM), and the affective faces go/no-go test. Questionnaires include the tiredness symptoms scale (TSS), Karolinska and Stanford sleepiness scale (KSS, SSS), the Profile of Mood States (POMS), and visual analogue scales (VAS), all related to mood and wellbeing. Immediately before initiating the test-battery, biological materials (blood and saliva samples) will be collected. The EEG/fMRI imaging will consist of a ~1hour awake part, followed by a ~1hour sleep opportunity. This protocol allows for the assessment of cerebral glymphatic flow under standardized wakefulness. Moreover, it provides an opportunity to measure glymphatic flow during a nap, at low sleep pressure (without sleep deprivation). Finally, the cognitive test battery before and after fMRI/EEG imaging will allow us to compare changes in cognitive performance with the intensity of glymphatic flow.

4. Sleep deprivation and adrenergic challenge: Two days succeeding the baseline scan, the same 25 healthy subjects that underwent the baseline night will be included in a double blind, placebo-controlled, crossover, sleep-deprivation study (Figure 1). In the first week, sleep-deprivation will be initiated two days succeeding the baseline scan. The second sleep-deprivation routine will be performed a week later, without a baseline imaging session (see Figure 1 for further details). The one-week delay allows subjects to recover from the lack of sleep. Sleep is assessed by a portable EEG device, either at home, or in a sleep clinic. EEG monitoring will be done at baseline, the nights before sleep deprivation

starts, and in the recovery nights. Additionally, to validate the applied cognitive tests, 20 subjects who only performs cognitive testing during a single round of sleep deprivation (without being MR scanned and without receiving the carvedilol/placebo treatment) will be included.

On the night of sleep-deprivation, subjects will be kept awake while under constant supervision by a researcher. After the night without sleep, subjects will undergo the same protocol as during the baseline day. This will ensure that all questionnaires, cognitive tests and EEG/fMRI data are directly comparable to the baseline. Placebo, or the adrenergic antagonist "carvedilol" (25 mg), is perorally administered immediately after the cognitive test battery is completed at ~15 o'clock, 31 hours after the prior standardized night of sleep. The imaging session is initiated 1h later, (after 32h of prolonged wakefulness). This timing ensures that EEG/fMRI imaging is performed when carvedilol has reached its maximum plasma concentration of approximately 50-75 ng/ml (C_{max}), which occurs about ~1 hour (t_{max}) after administration (GlaxoSmithKline, 1989; Gehr et al., 1999).

The protocol also ensures that data collected during sleep deprivation is directly comparable to the baseline, thereby allowing us to determine sleep-deprivation induced changes in the glymphatic system and to describe associated cognitive consequences. Blinding is ensured by having a person not involved in data acquisition or data analysis randomize and hand out the placebo or active drug. Moreover, the placebo controlled, cross over design allows us to quantify the effects of the carvedilol on the glymphatic system in humans, and determine whether adrenergic inhibition, as described in rodents, enhances the glymphatic system and the cognitive improvements after a nap. Sleep-deprivation ends by a standardized recovery sleep episode.

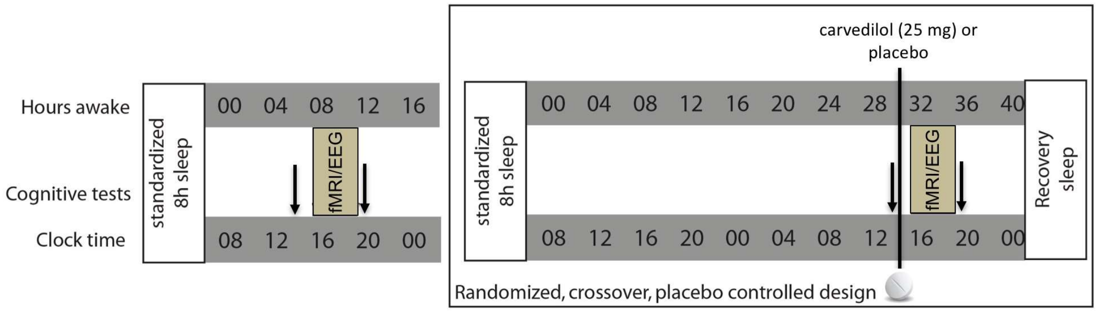

Figure 1: Overview of the described sleep-deprivation study. Baseline conditions (left) with fMRI/EEG imaging (yellow box) at ~16 o'clock (8h awake), followed by a randomized, crossover, double blind, placebo controlled study (black box, repeated twice). The adrenergic antagonist carvedilol is administered after 31h-sustained wakefulness (dotted line). fMRI/EEG imaging is performed 1h later, following 32h awake at the same circadian time point as baseline (~16 o'clock). 2h before (30h awake), and 30 minutes after imaging sessions (~35 h awake) a cognitive test battery is performed (arrows), to assess the effects of the adrenergic intervention and sleep in the MR scanner. Before study enrolment (not illustrated) an 8h screening night with high density EEG

is performed to exclude subjects with sleep disorders.

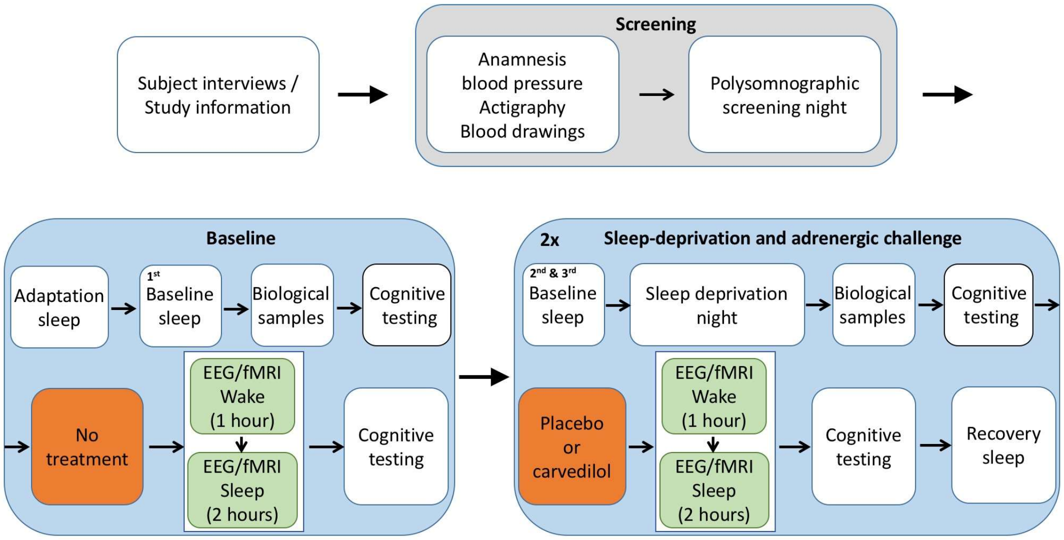

Figure 2: Detailed flow-chart of the described sleep-deprivation study.

Brief overview of the experimental elements:

Interviewing:

1) Entry questionnaires and generalized assessments of traits.

Screening:

1) Anamnesis and blood pressure measurement.
2) Activity monitoring and outpatient polysomnographic screening in sleep clinic.

Baseline protocol:

1) Standardized 8-hour adaptation and baseline sleep monitored at home or in sleep clinic.
2) Anamnesis, blood pressure check and collection of biological material (blood, saliva and urine samples).
3) Questionnaires and cognitive testing.
4) Simultaneous MR and EEG recordings of the brain during wakefulness and during a sleep opportunity.

Sleep-deprivation and adrenergic challenge:

1) Standardized 8-hour baseline sleep monitored at home or in sleep clinic.
2) Anamnesis and blood pressure check.

3) Supervised nocturnal sleep-deprivation.
4) Intervention (placebo or carvedilol).
5) Questionnaires and cognitive testing.
6) Simultaneous MR and EEG scan of the brain in wakefulness and sleep.
7) Standardized recovery sleep monitored at home or in sleep clinic.

The study will be performed exclusively with healthy young participants. The treatment is administered in a double-blind, placebo controlled, cross-over fashion. Unblinding will be possible in case of a medical emergency.

Driving and operating heavy machinery

Volunteers will be under constant supervision on the days of drug administration and sleep-deprivation, during which time subjects will not be allowed to drive or operate heavy machinery. Following recovery sleep (≥ 8 hours) subjects are expected to have sufficiently recovered from sleep deprivation (Van Dongen and Dinges, 2005) and from the effects of adrenergic modulation to again perform normally. Subjects will be neurologically tested before they leave the hospital to ensure that they are sufficiently recovered. The peroral administration of 25 mg carvedilol is expected to result in a plasma concentration of about 3.1 mg after 21 hours (44.2 μg/kg in a 70 kg adult). As such, following recovery sleep, the subjects do not need to take further precautionary measures.

Detailed description of the experimental protocol

1) Anamnesis and medical examination:
   Detailed information on past illnesses, hereditary predispositions, tobacco and alcohol consumption are acquired. A clinical examination of blood pressure, somatic and neurological functions is performed.

2) Interview questionnaires:
   Subjects will be required to complete questionnaires related to previous illnesses and traits. Trait questionnaires are about, personality, impulsiveness, stressful life events, sleep quality and chronotype. This will provide us with detailed information about wellbeing and sleep habits. Additional information to the questionnaires used can be found in the supplementary section "Questionnaires".

3) Actigraphy and polysomnographic screening night:
   Actigraphy with a wrist activity monitor is measured a week before the screening night to assess the endogenous circadian rhythm of the subjects. No restrictions are enforced during this week. Afterwards, subjects are inscribed as outpatients to a sleep clinic where they undergo a screening night. The screening night is important for excluding

neurological sleep disorders and extreme chronotypes. EEG, ECG, EMG, oxygen saturation and breathing during the sleep will be assessed. Data will be recorded and analysed in accordance with the American Association of Sleep Medicine (AASM, 2007/12). This will ensure that subjects suffering from sleep apnoea, restless legs syndrome, cardiac irregularities or a related undiagnosed sleep or rhythm disorder are excluded.

4) Collection of biological material:
   Urine samples: Urine samples will be acquired before baseline measurements, and before both sleep deprivation sessions in order to screen for drug consumption and (for women) to do a pregnancy test. The total amount of urine collected will not exceed 100 ml.
   Saliva samples: Saliva samples will be repeatedly collected at home and on the test days, and these samples will be used to measure stress hormone levels, including cortisol and testosterone. Home samples will be forwarded to the research unit for analysis. The amount of saliva collected will not exceed 75 ml in total volume.
   Blood samples: Blood samples used to determine relevant genotypes and to measure various biomarkers and biochemical variables will be taken during the first anamnesis (before the screening night, see Figure 2), when subjects are initially enrolled in the study. The total volume of withdrawn blood may amount up to 200 ml and is only performed once. Biochemical blood samples are acquired to ensure that subjects are healthy, including liver and kidney function tests, anemia (including s-transferrin), thyroid tests (thyroideatal), immune system assessments and blood sugar level measurements. Moreover, samples will be used to investigate specific molecular and genetic markers associated with the regulation of sleep and wakefulness. More exactly, the molecular measurements and DNA testing are used to investigate molecular markers related to brain-derived neurotrophic factor (BDNF) expression, CYP450 activity, adrenergic, adenosinergic, dopaminergic and serotoninergic signaling. It has previously been suggested that genotype status may affect the pharmacological response to adrenergic medications (Taylor, 2007; Johnson and Liggett, 2011; Sehrt et al., 2011). As such, genetic status may be included as a variable in the data analysis, and data can be corrected in relation to the individual subject's genotype.

5) Questionnaires and cognitive tests:
   State-like performance, sleepiness and wellbeing will be assessed briefly before and after imaging sessions with a duration of about 1 hour per time. The questionnaires are related to the subject's psychological well-being (transient), and include: mood (POMS), perceived stress (visual analogue scales) and sleepiness (KSS, SSS, TSS).
   Moreover, neuropsychological performance, including emotional memory (verbal

affective memory test), psychomotor vigilance (PVT test), risk taking and updating (Cambridge gambling task) affective responses (affective go/no-go test), will be assessed on a personal computer. The self-reported data and cognitive tests, will allow us to obtain an understanding of the psychological effects of sleep deprivation, and evaluate the effects of adrenergic inhibition in healthy subjects. It will also allow us to estimate the beneficial effects the sleep episode in the scanner. Additional information to the questionnaires used can be found in the supplementary section "Questionnaires".

6) Brain imaging:
   An MRI scanner is composed of a long tube surrounded by a coil that forms a powerful magnetic field. Test subjects are placed in the centre of the tube in a supine position. By changing gradients in the magnetic field and transmitting radio waves, resonance of the atomic nuclei of the brain can be induced. The subject may register changes in the sounds the MRI makes depending on the specific MRI pulse sequence, but beside these changing sounds, the subjects are unaware of the imaging and the scan is entirely non-invasive. To quantify glymphatic flow, different standardized fMRI protocols will be applied. These include changes in brain blood flow (ASL), resting state blood oxygenation imaging (rs-fMRI) and ultra-fast magnetic resonance encephalography (MREG). To quantify whether or not subjects are awake or asleep, EEG will be measured simultaneously with MRI, using MRI-compatible EEG equipment. Because sleep can only reliably be quantified using EEG, the use of simultaneous EEG and fMRI is in our study protocol is essential. We do not expect to be able to identify any obvious clinical effects of carvedilol.

7) Monitoring of sleep:
   To assess the quality and intensity of sleep at: 1. baseline, 2. before the initiation of sleep deprivation, 3. in recovery nights, and 4. Throughout prolonged wakefulness; subjects will be equipped with special transportable home EEG devices during relevant nocturnal sleep periods and during prolonged wakefulness. These non-invasive devices can measure the EEG and thereby sleep quality and intensity. The device also monitors the sleep onset and wake up times to ensure compliance with the study protocol. Sleep EEG will be monitored at home if subjects live in the Copenhagen area and have a reasonably dark, quiet and undisturbed bedroom. If not, sleep will be monitored using the same EEG equipment in a sleep clinic.

Data processing and statistics

MR and EEG data:

The analysis of the MRI and EEG data will be performed at the Neurobiology Research Unit (NRU), Rigshospitalet. Data will be processed and analyzed according to the most recent

scientific standards using SPM (Wellcome Department of Neuroimaging), FreeSurfer (Athinoula A. Martinos Center for Biomedical Imaging), MATLAB (MathWorks, Inc.) or similar data processing software. Potential further processing in other national and international laboratories may occur, yet always in accordance with the Danish Act on processing of personal data.

Statistics:
The general aim of the study is to establish the extend of sleep-dependent glymphatic flow in healthy adults. The following statistical analyses have been scheduled:

- Comparison of MREG data between wakefulness and sleep under baseline conditions (paired t-tests) will reveal whether glymphatic flow is enhanced by sleep.
- Correlation analysis between sleep intensity in the scanner (EEG delta power) and the simultaneously measured glymphatic flow to show that the two measures are associated.
- Correlation analysis between MREG data and changes in questionnaire variables and cognitive test performance, to investigate if subjective and objective improvements of a nap is associated with glymphatic flow.
- Comparison of placebo treated MREG data in the sleep-deprived condition with those measured at baseline to investigate if sleep deprivation enhances glymphatic flow.
- Comparisons of treatments (placebo vs. carvedilol) for the MREG data at the sleep deprived condition to show that adrenergic antagonism enhances glymphatic flow.

The comparison of MREG data across vigilance states, before and after sleep deprivation and with or without treatments, will initially be assessed with a 3-way repeated measure mixed model ANOVA with the factors condition (baseline, sleep-deprived), vigilance state (sleep, awake) and treatment (placebo, carvedilol), according to statistical standards. A similar procedure will be used for other variables when appropriate.

Required sample size:
Statistical power analysis for simple two-tailed paired t-test has been performed in G*power (G*power 3.1 software). Effect sizes (dz) are based on the data from (Xie et al., 2013), which suggests a dz between sleep and wakefulness for glymphatic flow of 2.2, and a dz between wakefulness and adrenergic inhibition for glymphatic flow of 0.9. With an alpha level = 0.01, and 85% power, the estimated sample size is n=7 and n=20, for the two parts, respectively. Taken together, this suggest that a sample size of 25 healthy individuals is sufficient to reveal the beneficial effects of sleep loss and adrenergic inhibition on glymphatic flow.

Research biobank

As described above, in this project biological material such as blood and saliva samples will

be collected in order to measure relevant biomarkers, to do genotyping and to ensure that all subjects are healthy. The collected samples will be analysed in larger portions in order to minimize measurement noise, and therefore we will for this project create a research biobank. Data collection for this will be reported to the Danish Data Protection Agency. The research biobank is terminated when the project is concluded, which is expected to be no later than December 31st, 2021. If any excess biological material is still available at this time, it will be transferred to the CIMBI Biobank, which is an approved, existing biobank under the Neurobiology Research Unit, Copenhagen University Hospital, Rigshospitalet. All collected material will be of relevance to this project, and no extra material will hence be collected exclusively for later use in the CIMBI Biobank.

Blood tests

Clinical biochemical tests: To ensure that subjects are healthy, blood samples are taken and used for biochemical tests that may indicate abnormalities or disease. The tests include liver and kidney function tests, anaemia (including s-transferrin), thyroid function, immune system assessments and blood sugar level measurements.

Biomarkers and genetic testing: Used for genotyping of polymorphisms related to sleep-wake regulation and the adrenergic signalling. These include determination of the brain-derived neurotrophic factor, and polymorphisms associated with CYP450 activity, adenosine, dopamine and the serotonin neurotransmitter systems. These targets may be used as variables in the subsequent data analysis, in particular to allow us to correct results with respect to subject's individual genotypes.
Blood samples are taken during the first anamnesis (before the screening night, see figure 2), when subjects are initially enrolled in the study. The total volume of withdrawn blood may amount up to 200 ml and is only performed once.

Saliva samples

The subject will be repeatedly asked to collect saliva samples, both at home and on test days. The home samples are forwarded to the research unit for measurement of biomarkers, including cortisol, melatonin and testosterone. Further information can be found in the "Collection of biological material" section above. The amount of saliva collected will not exceed 75 ml in total volume.

The Cimbi Database & Cimbi Biobank

As part of a coherent set of previous research projects, all of which have had as overall objective to relate imaging studies of the brain's serotonin system to genetic and biochemical markers, behavior, personality traits and memory function, NRU has for more than a decade

together with its collaborating partners in the Center for Integrated Molecular Brain Imaging (CIMBI) systematically studied a large group of healthy subjects, as well as smaller groups of neuropsychiatric patients, and thereby been able to establish a unique and internationally recognized research database (the CIMBI database) containing data from a considerable number of PET and MRI scans, neuropsychological tests, questionnaires and biochemical measures as well as an associated biobank (the CIMBI Biobank) containing all the collected biological samples. The CIMBI Database and Biobank have been approved by the Danish Data Protection Agency (protocol number 2007-58-0015 [local journal number 30-0291]).
By collecting data from a relatively large group of people, it is possible to identify patterns and correlations that we would not otherwise be able to demonstrate. To reveal these complex relationships, sophisticated mathematical models and statistical methods are used. The primary purpose of the Database is to generate new and novel hypothesis. Since data collected in the current project is valuable, unique and costly to obtain, and since it is important that such research data can be reused for scientific purposes to the fullest and most reasonable extent, data and excess biological material from the current project will be transferred to the CIMBI Database & Biobank after a specific approval for this has been obtained from the Danish Data Protection Agency.

Feasibility and ethical considerations

Facilities

The Neurobiology Research Unit (NRU) has years of experience with the use of neuroimaging modalities for the functional characterization of the human brain. All necessary equipment, software and research expertise to the project, are available from the collaborating laboratories. A broad and dedicated staff of physicians, neuropsychologists, pharmacists, nurses, molecular biologists, radio chemists, engineers and physicists are present in the co-operative staff. It is a psychologist, or another qualified person under the supervision of such an individual, who will be performing the neuropsychological evaluation.

Expected duration

The study will be initiated when approval has been obtained from the Regional Research Ethics Committee of the Capital Region of Denmark and may run until the end of 2021.

Financial support for the study

The experiments are performed at the Neurobiology Research Unit (NRU) at Copenhagen University Hospital (Rigshospitalet). NRU is part of the Center for Experimental Medicine NeuroPharmacology (NeuroPharm), which is supported with 15.6 mio DKK by the Innovation Fund Denmark and 4.8 mio DKK from the Lundbeck Foundation until 2021. The described project has already received 700.000 DKK in support from the Lundbeck

Foundation. Additional funding will be sought in the course of the study from several national and international foundations. However, neither existing nor future funding, will be allowed to influence the design, execution, data analysis or publication of results. When additional funding is acquired, the research ethical committees will be informed, and relevant participant information will be adapted and submitted to the research ethical committees. All funding is managed through the research accounts at Rigshospitalet, which are subjected to public control. The involved researchers have no private or economic interests in the funds and institutions that support the project. No support has been sought from commercial sponsors.

Principal investigator

The principal investigator is Gitte Moos Knudsen, who is head of the Neurobiology Research Unit where the work will be performed. Professors Maiken Nedergaard and Poul Jennum are collaborating researchers together with postdoc fellow Sebastian Camillo Holst.
Additional personnel may be associated with the study at a later date.

Rules for publishing

All results, positive, negative, as well as inconclusive will be published. There is no financial or corporate funding and the study is solely investigator driven. The results will be sought to be published in reputable journals that publish research related to the brain (e.g. Brain, Neuroimage, Nature Neuroscience, the journal of Neuroscience, etc.). Co-authorship is settled according to the Vancouver rules.

Financial compensation

The study is not associated with any health-related direct benefits for the participants. Therefore, volunteers are provided compensation for lost wages. According to the study schedule, this includes 500 DKK for completing the interview, interview-questionnaires and screening night, 1.000 DKK for completing the baseline measurements and 2.000 DKK for each completion of the sleep deprivation and adrenergic challenge. Subjects who only complete a single round of sleep deprivation without being MR scanned and without receiving carvedilol/placebo will receive 1.000 DKK. The maximum total compensatory fee for subjects completing the study is therefore 5.500 DKK. This remuneration is taxable as B income for the recipient. Fees will be paid jointly, or per day of participation if agreed. The amount will be paid to participants publicly recognized personal account (NEM-konto).

Insurance

No special conditions apply.

Confidentiality

During the recruitment phase, sensitive personal information will be acquired. Paper forms and other sensitive material on paper, will be kept in a locked file. Electronic information that can be traced to an identifiable person will be stored on password-protected computers behind secure "firewalls" in accordance with the Danish Act on processing of personal data. Tissue samples (blood and saliva) are labeled with unique identification numbers and stored safely as a research biobank in locked freezers or locked freezer rooms, equipped with alarms, for processing in larger portions. The key to the matching CPR numbers will be kept strictly confidential. After project-related analyses have been conducted, any excess material will be transferred to the CIMBI Biobank, as described above, and will eventually be destroyed according to the CIMBI Biobank approval (2007-58-0015, 30-0291). Blood samples from the CIMBI Biobank can only be (re-)used after new specific approval from the Research Ethics Committees. Subject names will never be used in scientific publications or presentations. Information that can identify a trial participant, will, in accordance with Danish laws ("Autorisationsloven", "Sundhedsloven", and the law on doctor-patient confidentiality) be strictly confidential and not available to anyone other than the staff working on the project. Subjects are not informed of acquired genotypes, mainly because health aspects of having a particular genotype is not sufficiently documented.

Patient medical record

In this project, no information from the participants' medical journal will be used.

Risks and discomforts

Adverse reactions to carvedilol

Carvedilol is a selective α_1-and β-adrenergic blocker and a racemic mixture. The drug blocks β_1 and β_2 adrenoreceptors, via S(-) enantiomer activity with a high dissociation constant (K_D) of 0.9 nM. The α_1-adrenergic blocking activity on the other hand, is present in both R(+) and S(-) enantiomers with a dissociation constant (K_D) of 11 nM (Ruffolo et al., 1990). Carvedilol is lipophilic and readily crosses the blood-brain barrier (Stenehjem et al., 2009; Wang et al., 2011). Clinically, carvedilol is used to treat mild to moderate congestive heart failure (CHF) by promoting vasodilation and vascular resistance. The planned amount of 25 mg, is a normal, safe, and well tolerated pharmacological dose, often used in healthy controls (GlaxoSmithKline, 1989; Sehrt et al., 2011).

If anything, there will be only mild and temporary side effects such as dizziness or nausea. No long-term effects of participation in the study are expected. A nurse, a medical student or a medical doctor will be present when the drug is given and to monitor subjects. In the unlikely event that the volunteers experience severe hypotension and bradycardia, appropriate medical treatment (atropine, blood volume expansion, intravenous glucagon or

dobutamin) will be given.
Following imaging and cognitive testing, subjects are escorted home (or to the sleep clinic) where EEG monitoring equipment is mounted and subjects allowed to sleep for at least 8 hours.

Incidents and side-effects
To avoid incidents, blood pressure is examined and subjects are questioned on the common side effects of carvedilol immediately before going into the MR scanner. After imaging the subjects are questioned again, before beginning with the cognitive test session. Side-effects may include: dizziness, fainting, reduced heart rate and lowered blood pressure. Also, shortness of breath, chest pain or swelling of the extremities may occur. Subjects are monitored for potential side effects until they go to sleep 9 hours post treatment.

Definition of adverse events and countermeasures:
For carvedilol, adverse events include illness or discomfort that require medical attention. Expected adverse reactions are those associated with already known side effects of carvedilol. Any other adverse event will be characterized as unexpected.
If an unexpected side-effect should occur, it will be reported the Board of Health (Sundhedsstyrelsen) using the e-form. Serious adverse reactions or events requiring hospitalization, resulting in persistent or significant disability, death, life-threatening conditions, incapacity, congenital abnormalities or birth defects are unlikely to occur as a consequence of the intervention.

Blood drawings and biological materials
At the first interview, and before imaging sessions, intravenous blood samples will be taken from a large vein near the elbow joint. Blood sampling may sometimes result in a hematoma (bruise) at the injection site. Blood drawing equipment will be handled by an experienced person under medical or nursing observation. When blood drawing is complete, haemostasis is secured by manual compression and subjects are instructed to sit relaxed in a chair for a few minutes. Infections occur extremely infrequent. The amount of blood drawn will be of maximum 200 ml and therefore does not exceed what is normally taken from a blood-donor visiting the blood bank (i.e. a maximum of 500 ml). Therefore, minimal discomfort is expected. In cases where blood and urine samples reveal drug use, subjects will not be confronted with the observation and the information will not be disclosed to third parties, but they will be excluded from the study.

Imaging and scans
The study will be performed on an MR scanner that is also used for normal clinical

diagnostics. The scanner meets IEC standards, which is the established international requirement to MRI equipment. The emitted radio waves can heat biological tissue slightly, but only to a negligible degree. MR scans have no medical side effects. MR scans only possess risks to people with foreign metal objects in their body (e.g. metal in the eyes, pacemakers, insulin pumps, artificial heart valves, artificial stapes – i.e. artificial ear bones, metal clips in the brain or heart). As is the clinical standard, our questionnaires will address and ask about such foreign metal objects, and the information will appear on the screening questionnaire under contraindications to the MRI. Some subjects become anxious or claustrophobic from lying in the scanner due to its tubular design. The staff at the MRI scanner is trained to create a secure environment around the scans, and the subjects can have the scanning interrupted immediately at any given time. Noise from the scanner will be diminished by earplugs or by MRI compatible headphones. The scans last a maximum of 270 minutes. It can be somewhat uncomfortable to lie still for such a long time in the scanner, but the staff will always be present during the scan and assists if the scan becomes uncomfortable. If subjects decide that they are not able to fall asleep in the scanner, the scan time is shortened or aborted. The scan will also be shortened or aborted if the subjects wake up in the scanner and are unable to fall asleep again.

Neuropsychological testing

Most people find it exciting to perform neuropsychological testing and perceive it as entertaining. Due to the cross-over, placebo controlled design, some tests are repeatedly performed. However, some people may perceive the testing as quite strenuous, especially since they are asked to make an effort in answering correctly and to the best of their ability. The testing will not include psychoanalytic elements or questions that can be perceived as delicate or crossing personal boundaries.

Safety measures

The neurophysiological testing and MR imaging will be performed by skilled staff, including physicians experienced with the techniques. Side-effects from carvedilol are checked before and after the imaging sessions, including blood-pressure measurements, to test for sudden events of hypotension. In case severe side effects from carvedilol occur, and in case these side effects cannot be corrected by the support staff, an appropriate treatment with intravenous glucagon will be used to rapidly restore well-being.

The staff performing the scans are also trained in the treatment of acute allergic reactions, and there is immediate access to medicines and equipment for treating such conditions.

When subjects finish imaging and cognitive testing on the sleep deprived day, they will be accompanied either home, or to the sleep clinic, by a member of the staff. This ensures that they are not a danger to themselves or others in the traffic, and ensures that the home EEG

monitoring equipment, used to record recovery sleep, is mounted correctly. The responsible staff will not leave the subjects before they are safely in bed.

Interruption of the trial

Subjects are free to abort the study at their request at any time. The study may also be aborted if subjects feel uncomfortable or sick during the study, or if they do not comply with the protocol. Termination may also be due to staff illness or equipment failure. Should this happen, subjects are informed of the cause of disruption and possibly be asked if they would participate again at a later date. If subject has unacceptable side-effects or pain from participation, the study will be interrupted, even though the subject is willing- or desire to continue.

The study itself may also be prematurely terminated if the results already at an early stage are sufficient to draw a scientific conclusion, or if new knowledge renders the study unnecessary or irrelevant.

Informing subjects on the outcome of the study

Subjects will be informed of the results of the study to the extent they wish. Because many of the scientific tests cannot be interpreted on an individual basis and require long-term data analysis, the information provided to individual subjects cannot directly be related to the scientific outcome of the study. In the oral and written information that subjects receive, it is emphasized that the scientific experiments conducted are not equal to a clinical diagnostic test. This implies that the subject participating in the study cannot consider their participation as a thorough health check-up, which can be used to rule out e.g. tumours and deformities. In case clear abnormalities are observed on the anatomical images, subjects will be informed and advised on how to best seek medical attention, but only if they have specifically consented to receive such information. Such findings may result in an entry into the subjects' public health records, which may affect their possibility and costs of life insurance. For that reason, subjects have to provide informed consent, acknowledging that their participating in the study potentially can change their ability to acquire a life insurance in the future, e.g. if abnormalities are observed on the MRI images.

Guidelines for obtaining informed consent

We will recruit from our already established database of potential volunteers who have expressed interest in participating in studies investigating the brain. Furthermore, subjects will be recruited from www.forsøgspersoner.dk. The advertisement used for this website is attached. Subjects will be approached by phone or e-mail. Specific information on the study is given orally by one of the above listed supervisors. Information's about the study may be exchanged via phone, or in a personal meeting. After the initial oral information has been

given, written information is sent or handed over. We attempt to always complete the first information meeting in person (at NRU), where the volunteers are offered to bring a bystander or family member. Volunteers are asked to reserve at least 20 minutes for the meeting, which allows the subjects to meet the responsible personnel or project nurse and get the oral information in a mitigating manner. Undisturbed meeting facilities are available at NRU. If volunteers are only accessible by phone, they are asked to confirm that they have 20 minutes available for the phone interview. If in doubt, another more suitable time for the interview is arranged. After the oral explanation of the study protocol, and the exchange of the written information, subjects are given a minimum of 2 days to thoroughly read the papers at home. Hereafter the subjects are encouraged to contact the supervising staff, by phone or email, in case they have any additional or clarifying questions. At this stage, subjects are permitted to submit or deliver the signed consent form. Only when we have received the signed consent form will we enrol the subject in the study and begin the experimental procedures.

Summary of the research ethical statement

The glymphatic system is a fundamentally new function of sleep, proposed to facilitate the clearance of interstitial waste from the brains parenchyma. This discovery may explain why sleep is such a fundamental and widespread biological process. Importantly however, evidence of a sleep dependent glymphatic system has not yet been described in humans. The proof of a human sleep dependent glymphatic system may lead to novel ways in which sleep can be improved or enhance, in both health and disease.

By taking advantage of the newly developed ultra-fast magnetic resonance encephalography (MREG) imaging, we will be able to provide the first evidence of the glymphatic system in healthy human volunteers and important biological questions will be addressed. These include whether glymphatic flow in humans is enhanced during sleep, and what the consequences of a night without sleep might have on glymphatic flow. Perhaps even more intriguing, we will answer the question whether glymphatic flow can predict improved cognitive performance following a nap. Finally, adrenergic antagonists have been shown to promote glymphatic flow in rodents. This suggests that adrenergic antagonists may be a novel pharmacological intervention to boost or improve sleep. By administering the adrenergic antagonist carvedilol, we aim to investigate whether the adrenergic system can boost sleep in humans. The use of novel imaging modalities and safe pharmacological intervention in humans can heighten drug development and reduce the use of animal models in preclinical phase 2 and phase 3 trials.

Side effects that may occur in the course of imaging or due to pharmacological interventions, are all expected to be transient. There are no known long-term risks of participating in this study. Because carvedilol at the prescribed dose has previously been used safely in similar

studies, side effects are highly unlikely. Moreover, with the access to glucagon to rapidly counteract any adverse effects of carvedilol, we believe that any discomfort caused by the study, is outweighed by the benefits to medical research and the scientific contributions this study is expected to provide. It is not expected that subjects themselves have any therapeutic or health benefits from their participation.

In the case of unacceptable, unexpected or serious adverse advents, the study can be terminated immediately. Termination may occur even though the subjects are willing to further participate. Therefore, the drawbacks and potential side-effects from participating in the study are considered fair.

References

Achermann P, Borbély AA (2011) Sleep homeostasis and models of sleep regulation. In: Principles and Practice of Sleep Medicine, 5 ed. (Kryger MH, Roth T, Dement WC, eds), pp 431–444. St. Louis: Saunders.

Bailey B (2003) Glucagon in β-Blocker and Calcium Channel Blocker Overdoses: A Systematic Review. Journal of Toxicology: Clinical Toxicology 41:595–602.

Gehr TWB, Tenero DM, Boyle DA, Qian Y, Sica DA, Shusterman NH (1999) The pharmacokinetics of carvedilol and its metabolites after single and multiple dose oral administration in patients with hypertension and renal insufficiency. Eur J Clin Pharmacol 55:269–277.

GlaxoSmithKline (1989) A study to assess the acute effects of a standard meal on the pharmacokinetics of carvedilol, after administration of a single oral dose of 25mg carvedilol in normal man. A9907.

Holst SC, Landolt H-P (2015) Sleep Homeostasis, Metabolism, and Adenosine. Current Sleep Medicine Reports 1:27–37.

Jessen NA, Munk ASF, Lundgaard I, Nedergaard M (2015) The Glymphatic System: A Beginner's Guide. Neurochem Res:1–17.

Johnson JA, Liggett SB (2011) Cardiovascular pharmacogenomics of adrenergic receptor signaling: clinical implications and future directions. Clin Pharmacol Ther 89:366–378.

Kiviniemi V, Wang X, Korhonen V, Keinänen T, Tuovinen T, Autio J, LeVan P, Keilholz S, Zang Y-F, Hennig J, Nedergaard M (2016) Ultra-fast magnetic resonance encephalography of physiological brain activity - Glymphatic pulsation mechanisms? J Cereb Blood Flow Metab 36:1033–1045.

Landolt H-P, Sousek A, Holst SC (2014) Effects of acute and chronic sleep deprivation. In: ESRS Sleep Medicine Textbook, 1st ed. (Bassetti CL, Dogas Z, Peigneux P, eds), pp 49–62.

Ruffolo RR, Gellai M, Hieble JP, Willette RN, Nichols AJ (1990) The pharmacology of carvedilol. Eur J Clin Pharmacol 38 Suppl 2:S82–S88.

Sehrt D, Meineke I, Tzvetkov M, Gültepe S, Brockmöller J (2011) Carvedilol pharmacokinetics and pharmacodynamics in relation to CYP2D6 and ADRB pharmacogenetics. Pharmacogenomics 12:783–795.

Shepherd G (2006) Treatment of poisoning caused by beta-adrenergic and calcium-channel blockers. Am J Health Syst Pharm 63:1828–1835.

Stenehjem DD, Hartz AMS, Bauer B, Anderson GW (2009) Novel and emerging strategies in drug delivery for overcoming the blood-brain barrier. Future Med Chem 1:1623–1641.

Taylor MRG (2007) Pharmacogenetics of the human beta-adrenergic receptors. Pharmacogenomics J 7:29–37.

Van Dongen HPA, Dinges DF (2005) Sleep, circadian rhythms, and psychomotor vigilance. Clin Sports Med 24:237–49–vii–viii.

Wang J, Ono K, Dickstein DL, Arrieta-Cruz I, Zhao W, Qian X, Lamparello A, Subnani R, Ferruzzi M, Pavlides C, Ho L, Hof PR, Teplow DB, Pasinetti GM (2011) Carvedilol as a potential novel agent for the treatment of Alzheimer's disease. NBA 32:2321.e1–2321.e12.

Xie L, Kang H, Xu Q, Chen MJ, Liao Y, Thiyagarajan M, O'Donnell J, Christensen DJ, Nicholson C, Iliff JJ, Takano T, Deane R, Nedergaard M (2013) Sleep drives metabolite clearance from the adult brain. Science 342:373–377.

Yasui M, Serlachius E, Löfgren M, Belusa R, Nielsen S, Aperia A (1997) Perinatal changes in expression of aquaporin-4 and other water and ion transporters in rat lung. J Physiol 505 (Pt 1):3–11.
