# Supplementary material for: Sleep deprivation and sleep intensity exert distinct effects on cerebral vasomotion and brain pulsations driven by the respiratory and cardiac cycles
Source: PLoS Biol. 2025 Nov 20;23(11):e3003500. doi: 10.1371/journal.pbio.3003500 (PMC12633874; doi:10.1371/journal.pbio.3003500)
Supplement: Attachment — Submitted filename: PBiologyReview.pdf [file pbio.3003500.s014.pdf]

This is an interesting paper that aims at investigating the levels of various types of pulsatile brain fluid (and presumably brain tissue) movement across sleep stages. This is motivated by their potential relevance for brain waste clearance, which an increasing number of studies suggest may be sleep-dependent, with particular contribution from slow-wave (SW) sleep or N3 sleep. Mechanistically, this clearance may be aided by CSF fluid movement, which previous studies have shown to result from various types of vascular pulsations. These include passive (barogenic) pulsations with cardiac and pulmonary cycles, and (slower) active (vasogenic) pulsations from brief changes in neuronal activity or changes in intravascular CO_2 from autonomic instabilities. These slower pulsations are referred to as LFO's by the authors.

Previous work has also shown that LFOs are stronger outside N3 and thus do not seem to explain previous experimental findings of increased tracer clearance with N3-like conditions (when SW density is highest). Here it is shown among other things, that N3 like conditions may have stronger barogenic pulsations. This would then suggest that N3-preferential clearance may be aided by these pulsations, rather than LFOs investigated previously. Although no causal relationship between barogenic vascular pulsations and waste clearance is demonstrated, or a mechanistic explanation of why these pulsations would be stronger in N3 sleep, the work provides intriguing information about the dependence of the strength of these pulsations on sleep stage. Most of my comments relate to methodological and interpretational aspects and discussion of existing literature. I believe addressing these comments would strengthen the work.

1. It is not clear why cardiac and respiratory pulsations would be stronger during N3 sleep compared to other states. For example, the cardiac cycle becomes more regular and slower during N3. Would increased pulsation power during N3 then mean that each pulse has stronger effects? Is it possible that some of the pulsation power during other states is missed due to it being outside the integration window? Stabilizing of cardiac and respiratory rate are well known properties of N3 sleep. This is partly because N3 is less punctuated with micro arousals. For example, cardiac rate changes during microarousals (which are most prevalent outside N3) can easily lead to rate increases of 0.2Hz, possibly moving energy outside the window. This can and should be tested by performing the same analysis that is performed on the MREG data to the peripheral cardiac signal. Ditto for the respiratory pulsations.
2. The differences in power of cardiac and respiratory pulsations between conditions are very small. For example, in Fig.4D, the difference appears to about 30% and 60% respectively between N3 and N2. Considering N2 duration typically is about twice that of N3 during overnight sleep, it is hard to see why N3 pulsations would be more beneficial for clearance than N2 pulsations. This is even harder when not using power of square root of power, which may be a more accurate gauge of pulsation amplitude. This needs more explanation/discussion.
3. Referencing is frequently inaccurate and should be corrected:
   Line 74: "Emerging preclinical evidence indicates that slow waves also play an important role in brain fluid dynamics (7)". Reference 7 is a review paper and it is

not clear what evidence in this paper supports this statement. Please cite direct evidence. Also, reference 7 cites the Fultz Science paper, but does not cite Picchioni (reference 46 in current manuscript), who shows, in contrast with Fultz, that a dominant contribution to CSF flow during N2 is autonomic, not neural activity. Autonomic activity (CO_2 changes, sympathetic vasoconstriction) is shown to be a major contributor to LFOs studied here and this is not mentioned at all in the entire manuscript, nor in Fultz. This should be corrected to provide an even-handed account.

Line 76: Reference 10 shows change in interstitial ion concentration (not interstitial space) during sleep.

Line 77: It should be made clear here that reference 9 refers to anesthesia, where slow waves may be of different nature. Also, Reference 11 shows increased CSF tracer infusion with both NREM and REM slow electrical activities. These are quite different, with the latter not typically called EEG slow waves

Line 81: "Respiratory and cardiac rhythms generate pressure waves that physically propel CSF into and through the parenchyma [15,16]". Reference 15 does not show influx into or through the parenchyma. Reference 16 shows influx into the spinal cord (not "through"), and contradicts 15 in that respiratory, not cardiac effects dominate.

Line 97: "intracranial pressure …. are also influenced by sleep stage [33,34]. These references talk about REM and N1/N2 having the strongest effects. How does this compare to the other references? More generally, some of the references talk about N3 like conditions being more important for clearance, and some showing evidence for other stages to have more importance. It would be helpful to clearly spell out this dichotomy and show how the current study may clarify it.

Line 358: "There were also no significant changes in blood pressure, heart and respiration rates in sleep-deprived wakefulness compared to rested wakefulness. That is, we do not find evidence that autonomic arousal alone explains the observed increases in LFOs, as proposed by Picchioni and colleagues [46]." First, the autonomic arousals reported by Picchioni are accompanied my *momentary* changes in respiratory flow rate, which are a major part the LFOs. Picchioni did not say anything about *average* heart/respiratory rates or blood pressure. The authors did not investigate this so should not make this statement. Further, Picchioni does not propose a contribution from autonomic arousal *alone*! They state that their findings suggest that: "CSF pulsations do not require slow waves and in part may result from autonomic arousal or autonomic instability". See their Figure S1 demonstrating that, during sleep, a combination of neural and autonomic effects contribute, with the latter dominating during N2.

Line 316: References [42,43] should be [43,44]?

Line 488: References 60 and 61 are not relevant to pulse sequence used here. Ref 62 should be cited instead here.

4. The manuscript would benefit from sharpening up the terminology. At times, imprecise, and confusing language is used:

"**brain oscillations/pulsations**": What is meant? Surely, the MRI resolution is insufficient to separate fluid (blood, CSF) from tissue. What is the MREG signal sensitive to? Likely not movement of brain tissue itself, whose visualization needs strong encoding gradients. Almost certainly, most of the pulsatility observed in this work comes from CSF, either in ventricular or subarachnoid space.

"**fluid**". What is meant? Blood or CSF?

"**cerebrovascular vasomotor**" what is this. Does this include all of CO_2, sympathetic, and local neural activity mediated effects? Maybe use "vasomotor" or "vasogenic" effects and make clear they can originate from various mechanisms (maybe by using a reference).

Line 92: "while light NREM sleep enhances CSF flow into the fourth ventricle", here and throughout manuscript, distinguish between net flow and pulsatile movement.

Line 149: "respiration" : respiratory rate

Throughout manuscript: Clarify that the CSF signal is taken from the ventricles, and that "grey matter" and "white matter" unavoidably contain a mixture of signals due to partial volume effects in light of the coarse resolution of MREG. In fact, the "grey matter" signal is likely dominated by subarachnoid CSF and this may even be the case for white matter. This should be clarified.

5. Several statements are not well justified**.**

   Abstract: "In summary, our findings indicate that sleep pressure promotes cerebrovascular oscillations, while sleep slow waves synchronise with respiration- and cardiac-driven pulsations in the brain parenchyma".
   This synchronization has not been demonstrated so this statement is not warranted.

   Line 323: "and that increases in CSF influx to the brain parenchyma will amplify these pulsations" CSF influx into the parenchyma has not been demonstrated, see above.

   Line 379: "the association between sleep slow waves – i.e. deeper sleep and higher delta power – and brain pulsation strength strongly support the recent suggestion that the synchronous neuronal activity of slow-wave sleep drive fluid and solute movement physio-mechanically into and through the brain [11,53]" How would higher delta power increase cardiac and respiratory cycle related pulsations?

   Line 387: "Consequently, we here provide support for the hypothesis that humans - alike mice – have a sleep- and slow-wave dependent exchange of CSF and interstitial fluid, clearing the brain of waste products". This statement I feel is inaccurate. I think what has been shown is that barogenic pulsations appear strongest during N3, potentially providing a mechanistic explanation for a previously observed preference for waste clearance to occur during N3-like conditions.

   Line 420: "these important findings offer crucial insights into how the vasomotor system in the brain responds to sleep deprivation, potentially serving as a means to mitigate the accumulation of brain waste products"

Line 422: “Furthermore, we illustrate that as sleep depth increases, brain pulsations induced by respiration and cardiac activity intensify in both grey and white matter. As these brain pulsations likely reflect brain fluid flow, our observation aligns with preclinical evidence of a slow-wave-dependent influx of CSF into the brain parenchyma.

6. The authors report an intriguing reduction in cardiac-frequency MREG power with NREM sleep versus placebo. Cardiac pulsations are presumably not related to myogenic mechanisms. Could the authors offer an explanation for this?
