# Supplementary material for: Sleep deprivation and sleep intensity exert distinct effects on cerebral vasomotion and brain pulsations driven by the respiratory and cardiac cycles
Source: PLoS Biol. 2025 Nov 20;23(11):e3003500. doi: 10.1371/journal.pbio.3003500 (PMC12633874; doi:10.1371/journal.pbio.3003500)
Supplement: Attachment — Submitted filename: CSFreview2.pdf [file pbio.3003500.s016.pdf]

The authors have done a commendable job revising the manuscript. While the revision is much improved, I still have lingering issues with terminology and inadequately supported claims. While I believe the results are of significant interest, I think these issues should be addressed before acceptance for publication.

Chiefly, the terminology often remains vague and/or ambivalent and at the same time suggestive, making the work prone to misinterpretation and appear to have unwarranted claims. What the authors measure are MRI signal fluctuations in different frequency bands. The manuscript uses numerous terms to tie these to physiological pulsations and to eventually equate them to movement of interstitial fluid in support of waste clearance.

For example, in the abstract alone, five different terms are used to describe pulsations and several of these are vague/hard to understand ("brain oscillations", "brain pulsations", "physiological brain pulsations", "cerebrovascular oscillations", "pulsations in the brain parenchyma"). Why not say : "we used rapid MRI to study vascular pulsations originating from cardiac and respiratory cycles as well as from myogenic mechanisms, all of which cause movement of CSF and brain tissue and may have relevance for glymphatic clearance".

Another vague term is "brain fluid flow" (as well as "waste clearing fluid flow", "brain fluid dynamics", "glymphatic flow"). What is this? First, fluid could mean CSF, blood, and interstitial (extracellular) fluid. Upon rereading the manuscript, I believe the authors mean the latter. Secondly, the authors need to distinguish between convective or "net" flow and pulsatile flow.

Related to the term "brain fluid flow", and critical to the claims in the manuscript, the authors seem to interpret part of the MREG signal to reflect interstitial fluid movement. This however does not make sense considering the minuscule pulsatile movements of brain tissue (see the literature on MRI displacement encoding, e.g. Greitz et al Neuroradiology 1992). In tissue, these can only be observed with the use of strong encoding gradients, and even then only in the phase data (not magnitude data). Previous MRI research has applied various types of MRI contrast (including T2* that the authors claim MREG uses. The understanding of this work is that cardiac and respiratory vascular pulsations result mostly in signals at or near the pial surface, and the slower pulsations (myogenic effects from neurovascular control and CO_2/sympathetic mechanisms) accentuate areas with high blood volume (grey matter, large veins and their surrounds). At the minimum, they authors should explain how MREG would be able to detect interstitial fluid pulsations, and what are potential pitfalls with this interpretation.

Detailed comments are interspersed (in red) with the authors' responses to the previous review below.

1. It is not clear why cardiac and respiratory pulsations would be stronger during N3 sleep compared to other states. For example, the cardiac cycle becomes more regular and slower during N3. Would increased pulsation power during N3 then mean that each pulse has stronger effects? Is it possible that some of the pulsation power during other states is missed due to it being outside the integration window? Stabilizing of cardiac and respiratory rate are well known properties of N3 sleep. This is partly because N3 is less punctuated with micro arousals. For example, cardiac rate changes during microarousals (which are most prevalent outside N3) can easily lead to rate increases of 0.2Hz, possibly moving energy outside the window. This can and should be tested by performing the same analysis that is performed on the MREG data to the peripheral cardiac signal. Ditto for the respiratory pulsations.

Authors' response:
Thank you for this insightful comment.
The reviewer asks whether our observations could be confounded by respiratory and cardiac differences between N3 sleep and other sleep states. We believe this is unlikely, as brain pulsation power in the respiratory or cardiac frequency bands was computed for each 30-second MREG epoch based on the simultaneously recorded respiration- and heart rate signals (see lines 622-637). That is, if heart or respiration rates changed in an individual during e.g. N2 sleep, the frequency window for assessing MREG spectral power was shifted accordingly. We understand if this critical information can be missed, due to the journal requirements where the method follows results. For that reason, we have now also added the information to Results:
Lines 209–2011:
"
.. MREG spectral power within the respiration and cardiac frequency bands was
2"
individually tailored for each 30-sec epoch based on simultaneously recorded respiration- and heart rate signals (see Methods)"
.
Moreover, as also described in the manuscript, we included epoch-specific respiration- and heart-rates as fixed effects in our linear mixed models to account for both the 1/F effect and potential influences of changing cardiorespiratory rates on brain pulsation power.
We have now added the following sentence to the model description:
Lines 675–676:
"To account for vigilance-state related changes in peripheral cardiorespiratory signals as well as the impact of..
As per the reviewer's suggestion, we have now applied the same analysis used on the MREG data to the peripheral cardiac and respiratory signals. When we assess the effect of sleep depth on respiration and heart rates, we find no change in respiration rate and only a slight increase in heart rate from N2 to N3 sleep (+1.6%, p = 0.01; Table S5). This finding is now added to the Results:
Lines 150-152:
"
…while the heart rate increased slightly during N3 vs. N2 sleep (1.6%, p = 0.01) with no change in respiration rate (S5 Table)."
That is, in this data set we did not see any major differences in heart rate between N2 and N3 sleep. We propose that this could be due to the volunteers being sleep deprived which may be associated with higher sympathetic nervous system activity and increased cardiovascular reactivity (Bigalke et al., 2021. DOI: https://doi.org/10.1096/fasebj.2021.35.S1.02387).

What I meant is that outside N3, K-complexes and micro-arousals lead to biphasic changes in heart rate over a brief (<30s interval) periods. This does not need to result in a change of epoch-averaged hear rate. However, associated pulsation energy can be missed as spectral band is broadened (to as much as 0.4 Hz!). To avoid this confound, the authors should perform the suggested analysis on the cardiac signal. This means analyzing the spectral power of the cardiac (e.g. ECG) signal. The analyses the authors refer to do not accomplish this.

2. The differences in power of cardiac and respiratory pulsations between conditions are very small. For example, in Fig.4D, the difference appears to about 30% and 60% respectively between N3 and N2. Considering N2 duration typically is about twice that of N3 during overnight sleep, it is hard to see why N3 pulsations would be more beneficial for clearance than N2 pulsations. This is even harder when not using power of square root of power, which may be a more accurate gauge of pulsation amplitude. This needs more explanation/discussion.
Authors' response
While we follow the logic of the reviewer re. the duration of N2 vs N3, we also think it is difficult to quantitatively assess the association between brain pulsation power and brain clearance, and that includes statements re. the expectations about what to expect from changes in power. What we do find, and for the first time in humans, is that N3 sleep is associated with higher brain pulsation power than N2. To the extent this comparison is possible, the magnitude of change in brain pulsations parallels findings of Xie et al. of a 60% expansion in interstitial space volume and a 90% increase in CSF tracer inflow between wakefulness and natural sleep in mice.
We have added the following to the Discussion in lines 399-402:
"Notably, when participants fell asleep and entered NREM sleep, the strength of respiration- and cardiac-driven brain pulsations increased by 73% and 35%, 3*which can cautiously be compared to the ~60% increase in interstitial space volume reported by Xie el al. in naturally sleeping rodents [8]."*
And lines 421-425:
"
.. However, as the quantitative impact of these stronger pulsations in N3 versus in N2 sleep is difficult to assess directly with our study design, it should be mentioned that the duration of N2 in a typical night's sleep is about twice as long as that of N3 sleep, potentially leaving more time for brain clearance to take place. This leaves open the question whether the cumulative time spent in NREM sleep or the total amount of sleep slow waves is more important for glymphatic function"

OK!

3. Referencing is frequently inaccurate and should be corrected:
Line 74: "Emerging preclinical evidence indicates that slow waves also play an important role in brain fluid dynamics (7)". Reference 7 is a review paper and it is not clear what evidence in this paper supports this statement. Please cite direct evidence.
Authors' response:
We would appreciate if the reviewer could point us to a more recent comprehensive and complete review on brain fluid dynamics, glymphatic regulation, and the proposed role of slow waves to introduce readers to the topic in the Introduction. Also, the review does not stand alone but is followed by citations of original research (Refs. 8–10).

I read all the four references the authors used to support their statement (refs 7-10), but none of them shows a causal relationship between SWA and brain fluid dynamics. Reference 9 does show a relationship, but it is only correlational. Therefore, this statement is not warranted.

Also, reference 7 cites the Fultz Science paper, but does not cite Picchioni (reference 46 in current manuscript), who shows, in contrast with Fultz, that a dominant contribution to CSF flow during N2 is autonomic, not neural activity. Autonomic activity (CO_2 changes, sympathetic vasoconstriction) is shown to be a major contributor to LFOs studied here and this is not mentioned at all in the entire manuscript, nor in Fultz. This should be corrected to provide an evenhanded account.

Authors' response:

Thank you for pointing this out.

We have now added the Picchioni reference (reference 41 in revised manuscript) to the Introduction line 106:

"Additionally, drowsiness and autonomic arousal appear to enhance LFOs in wakefulness [40, 41], ..
"

Ref 41 shows that autonomic arousal is dominant during N2 (not "drowsiness" or "wakefulness") so this statement is misleading. Better to state "LFOs are enhanced during drowsiness and light sleep [40,41] an have a dominant contribution from autonomic arousals [41]". By the way, the first discovery of this was by Fukunaga et al, MRI 24, 2006.

The following statement has also been revised to clarify that NREM sleep promotes CSF influx to the brain, without implying that this process occurs exclusively during sleep:

Lines 67-69: *"NREM sleep enhances the flow of cerebrospinal fluid (CSF) from perivascular spaces into and through the brain parenchyma, which in turn* 4*causes an increase in interstitial… space volume and an exchange of CSF and interstitial fluid [8–10]."*

In the Discussion (lines 382-387), we have also expanded our discussion of the potential contribution of autonomic arousal to the effect of sleep deprivation on LFOs:

"Lastly, because autonomic arousal has been proposed as a potential mechanism for LFO regulation [41], we examined markers of sympathetic activity between well-rested and sleep-deprived wakefulness. However, in our data, blood pressure, heart rate and respiration rate did not differ between the two conditions, indicating that factors beyond both neural slow waves and autonomic arousal likely contribute to the increase in awake LFOs following sleep-deprivation."

Line 76: Reference 10 shows change in interstitial ion concentration (not interstitial space) during sleep.

Authors' response: We would prefer to keep reference 10 in the manuscript for the following reason: Ding et al (Ref 10) investigate the relationship between sleep-wake states and changes in interstitial ion concentration but also demonstrate that changing the awake state to that of the sleep state (or vice versa) via administration of awake- or sleep-inducing artificial CSF, is associated with the expected increase in EEG delta power and with an associated increase in interstitial space volume. This paper thus presents an important piece of evidece that vigilance state is associated with interstitial space volume changes (see Fig 5B, C and D in Ding et al., 2016 paper).

Line 77: It should be made clear here that reference 9 refers to anesthesia, where slow waves may be of different nature. Also, Reference 11 shows increased CSF tracer infusion with both NREM and REM slow electrical activities. These are quite different, with the latter not typically called EEG slow waves

Authors' response: We agree and have now added the information that the mice in [9] were anaesthetized. Additionally, Reference 11 has been removed from this statement, but has been retained it in the Discussion section.
Lines 69-70:
"
..This influx of CSF strongly correlates with EEG slow waves in anaesthetized mice [9] and facilitates the ….
"

Line 81: "Respiratory and cardiac rhythms generate pressure waves that physically propel CSF into and through the parenchyma [15,16]". Reference 15 does not show influx into or through the parenchyma. Reference 16 shows influx into the spinal cord (not "through"), and contradicts 15 in that respiratory, not cardiac effects dominate.
5Authors' response: We acknowledge that the current statements may be somewhat imprecise. To address this, we have revised the text and incorporated three additional references to provide stronger support for the statements:
Lines 73-78: "*While sleep overall promotes brain fluid flow, it is believed that this flow is driven by an interplay of low frequency cerebrovascular oscillations, respiration, and the cardiac cycle. Respiratory in- and exhalations, as well as the heartbeat, generate pressure waves that propagate through the brain, physically propelling CSF into and through CSF spaces and the brain parenchyma [14-18].*

References 14-18 should be separated and placed after each relevant statement. I believe only 15 and 18 show evidence of parenchymal involvement!

In parallel, intrinsic cerebrovascular oscillations, also referred to as vasomotion, facilitate CSF-flow along periarterial spaces and into the brain [19,20].

Not sure what "*intrinsic cerebrovascular oscillations"* or "vasomotion" refer to. "Vasomotion" is a highly controversial term and originally was used refer to oscillations that originate in arteries. They have been observed in a specific frequency band in isolated arteries or in animals under physiological stress. I am not of aware of any human data that show such oscillations where the origin is in the arteries. Always, there is a potential other explanation (neural activity, or autonomic activity). Similarly, much of what is discussed in 19 and 20 is neurogenic (evoked by neuronal stimuli through neurovascular response) or possibly with an autonomic component. Why not stay away from "vasomotion" and just refer to LFO??

.
Line 97: "intracranial pressure …. are also influenced by sleep stage [33,34]. These references talk about REM and N1/N2 having the strongest effects. How does this compare to the other references? More generally, some of the references talk about N3 like conditions being more important for clearance, and some showing evidence for other stages to have more importance. It would be helpful to clearly spell out this dichotomy and show how the current study may clarify it.
Authors' response: We have intentionally taken a cautious approach in describing the relationship between ICP B-waves and sleep stage as [ref 34 and 35 in revised manuscript] have not (to our knowledge) been replicated, and report varying results depending on whether they assess B-wave frequency, amplitude, wavelength, or morphology. Based on these studies, we believe it is appropriate to say that sleep stages are associated with B-waves but the exact nature and stage-specific characteristics of this relationship remain too uncertain to state definitively in the Introduction.
We do, however, elaborate on these findings in the Discussion (lines 375–377) and have now slightly revised the relevant section of the text in the Introduction.
Lines 92-95:
"…invasively measured intracranial pressure (ICP) B-waves (0.5 – 2 waves pr min) – which are thought to reflect cerebrovascular oscillations [33] – are also influenced by the sleep-wake cycle and sleep stage [34,35].

”

We agree with the broader point that the Introduction would benefit from a clearer presentation of the current state of knowledge, the dichotomy in previous findings, and how our study may help reconcile this.

To address this, we have updated the following section of the Introduction:

Lines 96-110:

“However, understanding how changes in vigilance states and slow wave activity relate to fluid flow in the human brain requires non-invasive methods that allow for natural sleep during continuous measurements of both brain fluid dynamics and EEG. Accelerated brain imaging techniques, such as Magnetic Resonance Encephalography (MREG) [36], have emerged as valuable tools for this purpose. MREG is a functional T2-weighted imaging method with a temporal resolution on the order of milliseconds, enabling non-invasive assessments of low frequency brain oscillations (LFOs) as well as brain pulsations induced by the*
6*respiratory and cardiac cycles – all of which have been proposed to reflect brain fluid motion [16,37]. Previous studies have shown that NREM sleep (N1 and N2) enhances all three types of physiological brain oscillations [38], with sleep effects involving larger brain regions in N2 than in N1 sleep [39]. Additionally, drowsiness and autonomic arousal appear to enhance LFOs during wakefulness [40,41], while sleep deprivation specifically increases LFOs in N1 sleep [39]. Although the influence of circadian rhythm and sleep propensity on these findings remain unclear, they suggest that both NREM sleep, sleep depth and wake-related factors modulate MR-detected physiological brain pulsations, with potentially distinct effects on LFOs and cardiorespiratory brain pulsations.”*

Line 358: “There were also no significant changes in blood pressure, heart and respiration rates in sleep-deprived wakefulness compared to rested wakefulness. That is, we do not find evidence that autonomic arousal alone explains the observed increases in LFOs, as proposed by Picchioni and colleagues [46].” First, the autonomic arousals reported by Picchioni are accompanied my momentary changes in respiratory flow rate, which are a major part the LFOs. Picchioni did not say anything about average heart/respiratory rates or blood pressure. The authors did not investigate this so should not make this statement. Further, Picchioni does not propose a contribution from autonomic arousal alone! They state that their findings suggest that: ”CSF pulsations do not require slow waves and in part may result from autonomic arousal or autonomic instability”. See their Figure S1 demonstrating that, during sleep, a combination of neural and autonomic effects contribute, with the latter dominating during N2.

Authors’ response: We thank the reviewer for this comment.

We can see that the text could be interpreted as suggesting that Picchioni et al. claim that autonomic arousal alone drives LFOs. Our intention was to acknowledge their findings and to speculate whether the influence of autonomic arousal on LFOs could help explain the increase we observed following sleep deprivation.

We have now rewritten the sentence to:

Lines 382-387:

“Lastly, because autonomic arousal has been proposed as a potential mechanism for LFO regulation [41], we examined markers of sympathetic activity between well-rested and sleep-deprived wakefulness. However, in our data, blood pressure, heart rate and respiration rate did not differ between the two conditions, indicating that factors beyond both neural slow waves and autonomic arousal likely contribute to the increase in awake LFOs following sleep-deprivation.”

Line 316: References [42,43] should be [43,44]?

Authors’ response: Thank you for noticing this. The references have been corrected.

Line 488: References 60 and 61 are not relevant to pulse sequence used here. Ref 62 should be cited instead here.

7Authors’ response: Thank you for noticing this. We have replaced [60, 61] with [62].

4. The manuscript would benefit from sharpening up the terminology. At times, imprecise, and confusing language is used: “brain oscillations/pulsations”: What is meant? Surely, the MRI resolution is insufficient to separate fluid (blood, CSF) from tissue. What is the MREG signal sensitive to? Likely not movement of brain

tissue itself, whose visualization needs strong encoding gradients. Almost certainly, most of the pulsatility observed in this work comes from CSF, either in ventricular or subarachnoid space. "fluid". What is meant? Blood or CSF? "cerebrovascular vasomotor" what is this. Does this include all of CO_2, sympathetic, and local neural activity mediated effects? Maybe use "vasomotor" or "vasogenic" effects and make clear they can originate from various mechanisms (maybe by using a reference).

Authors' response: We thank the reviewer for the constructive feedback. We have now changed the text as detailed below (see A and B).

A) Brain oscillations/pulsations:

The terms *"respiration- and cardiac-driven brain pulsations"* are aligned with prior studies investigating MREG spectral power, i.e. MR-detected pulsations in the brain, within the respiratory and cardiac frequency ranges.

The term Low frequency brain oscillations (LFOs) denotes MREG spectral power in the 0.012-0.034 Hz frequency range and does not make any presumptions about the specific biological origin of this signal.

Oscillatory signals in the brain < 0.1 Hz are currently referred to by various terms in the literature (e.g., cerebrovascular oscillations, *slow oscillations*, *vasomotion*, *vasomotor pulsation*, *vasogenic oscillations*, *B-waves/pseudo B-waves*, etc.).

Here, we generally use 'cerebrovascular oscillations', but also incorporate some of the other terms into the manuscript to acknowledge this diversity.

However, we recognize that this could deserve a little more specificity and therefore tightened up the language in the manuscript.

Regarding the suggestion to elaborate on LFO mechanisms, we briefly address these in the Introduction (lines 77–81). A more detailed discussion of local mediators falls outside the scope of this study, as they were not directly investigated and compared here.

Lastly, as a common term for the three investigated physiological signals in the brain, we originally used *"brain oscillations."* However, reading through the paper again, we acknowledge the reviewer's point and have revised the manuscript to use individual descriptors where possible and now instead use the terms *"physiological brain pulsation"* when referring to all signals collectively.

B) MREG signal:

MREG is a functional T2*-weighted ultrafast imaging technique (10 Hz), meaning that the signal obtained every 100 ms is influenced by both the BOLD (Blood-Oxygen-Level-Dependent) signal and water in, for example, CSF and interstitial fluid spaces.

See my main comments. There is no evidence, and it is very unlikely that MREG specifically detects interstitial fluid movement. No physical mechanisms is presented to even lend support to this possibility.

8Due to the relatively low spatial resolution of the MREG signal, we agree with the reviewer that the signal in each voxel—both in grey and white matter—originates from blood and water in CSF spaces, the neuropil, and the vasculature.

As such, the measured signal oscillations reflect changes in these components that are synchronized with the respiration, heartbeat, or occur in the low-frequency range. The MREG signal strength is therefore likely influenced by CSF and blood flow, interstitial water/CSF content, and the compliance of brain tissue within the captured voxels. We elaborate on this interpretation of the signal in the Discussion (lines 340–358; see also reviewer response above) and have also added the current interpretation of MREG-detected brain pulsation to the Introduction (line 132).

To clarify the sensitivity of the MREG signal for the reader, we have added the information that MREG is T2*-weighted accelerated MR imaging techniques to both the Introduction (line 100) and the Discussion (line 340) of the manuscript.

Line 92: "while light NREM sleep enhances CSF flow into the fourth ventricle", here and throughout manuscript, distinguish between net flow and pulsatile movement.

Authors' response: The below sentences have been revised in accordance with the reviewer's suggestion.

Lines 88-89: "
…light NREM sleep enhances pulsatile movements of CSF in the fourth ventricle"
Line 353: *"… temporally associated effects on CSF oscillations."*
Lines 377-378: *"Similarly, both the slow oscillating CSF signal in the fourth ventricle and its.."*
Line 149: "respiration" : respiratory rate Throughout manuscript: Clarify that the CSF signal is taken from the ventricles, and that "grey matter" and "white matter" unavoidably contain a mixture of signals due to partial volume effects in light of the coarse resolution of MREG. In fact, the "grey matter" signal is likely dominated by subarachnoid CSF and this may even be the case for white matter. This should be clarified.
Authors' response:
Thank you for noting the inconsistency in the use of "respiration" versus "respiratory rate." We have now revised the manuscript to ensure that "respiration" is used when the word is a noun, and "respiratory" is used when it is an adjective.

This was not a noun versus adjective issue. The respiratory cycle can affect MREG through either changes in respiratory rate and/or depth. The authors should specify what is meant.

In Methods (lines 563-568), we clarified already how the CSF signal is defined. We have now further elaborated on this in the limitations section:
9Lines 450-453:
"Moreover, given the coarse spatial resolution of MREG, we assessed CSF pulsations in the lateral ventricles to minimize partial volume effects from adjacent tissues. As a result, our data do not allow for examination of CSF pulsations in the fourth ventricle, as has been done by other studies cited here."
Regarding the point that grey and white matter masks include interstitial fluid and CSF, this is a central argument supporting our interpretation that the MREG signal reflects water content and/or tissue compliance. We hope this is clearly conveyed in the following revised statement from the Discussion section:
Lines 343-350:
"Our data support this interpretation for respiration- and cardiac-driven brain pulsations demonstrating that, irrespective of vigilance state, these pulsations are significantly stronger in CSF spaces than in grey and white matter. During rested wakefulness, they are also more pronounced in grey matter compared to white matter

My point was that the "grey matter" mask has more CSF than the "white matter" mask. So if the pulsation energy is mostly in CSF, then based on partial volume effects you expect more MREG signal in CSF versus GM versus WM, irrespective of the tissue water content or compliance or interstitial fluid ("brain fluid") content. This gets back to the imprecise use of language mentioned above.

. Considering the differences in water and CSF content, as well as tissue viscosity, across these three brain regions [43,44], our results indicate that the relative water content of a given region (or voxel) is closely associated with the strength of the MREG-detected respiration- and cardiac-driven brain pulsations. Together, this suggests that an increase in CSF influx to the brain parenchyma will amplify brain pulsatility."

This is a stretch, see above.

5. Several statements are not well justified.
Abstract: "In summary, our findings indicate that sleep pressure promotes cerebrovascular oscillations, while sleep slow waves synchronise with respiration-

and cardiac-driven pulsations in the brain parenchyma". This synchronization has not been demonstrated so this statement is not warranted.

Authors' response: We acknowledge that the original statement could be interpreted as implying that EEG slow waves occur simultaneously with respiration- and cardiac-driven brain pulsations, which we do not demonstrate. We have now revised the statement in the abstract:

Lines 55-57:

"In summary, our findings indicate that sleep pressure promotes cerebrovascular oscillations, while slow-wave-rich sleep amplifies respiration- and cardiac-driven pulsations in the brain parenchyma."

Line 323: "and that increases in CSF influx to the brain parenchyma will amplify these pulsations" CSF influx into the parenchyma has not been demonstrated, see above.

Authors' response: In context, this specific sentence appears in the Discussion and reflects our interpretation of what an increase in the strength of MREG-detected pulsations in grey and white matter may indicate.

The sentence has been revised to better convey this interpretation.

10Lines 349–350:

"Together, this suggests that an increase in CSF influx into the brain parenchyma will amplify brain pulsatility."

This is a stretch, see above.

Line 379: "the association between sleep slow waves – i.e. deeper sleep and higher delta power – and brain pulsation strength strongly support the recent suggestion that the synchronous neuronal activity of slow-wave sleep drive fluid and solute movement physio-mechanically into and through the brain [11,53]" How would higher delta power increase cardiac and respiratory cycle related pulsations?

Authors' response: If sleep slow waves – which are more prominent with higher delta power and deeper sleep – increase influx of CSF into the brain parenchyma, we would expect respiration- and cardiac-driven brain pulsations to likewise strengthen as delta power and sleep depth increases. This is what we observe.

We have changed the Discussion to make this clearer:

Lines 395-398:

"Because the strength of these MREG detected physiological brain pulsations are likely to scale with tissue water content, their enhancement during NREM sleep is compatible with a sleep-dependent increase in CSF influx to the brain parenchyma, which could be explained by an expansion of extracellular space volume, similar to observations made in the mouse brain [8,10]."

This is a stretch, see above.

And

Lines 409-413:

"

.. the association between more sleep slow waves – i.e. deeper sleep and higher delta power – and the increase in strength of respiration and cardiac driven brain pulsations is in line with the recent suggestion that the synchronous neuronal activity of slow-wave sleep may physio-mechanically drive fluid and solute movement into and through the brain [51, 52]."

Line 387: "Consequently, we here provide support for the hypothesis that humans - alike mice – have a sleep- and slow-wave dependent exchange of CSF and interstitial fluid, clearing the brain of waste products". This statement I feel is inaccurate. I think what has been shown is that barogenic pulsations appear strongest during N3, potentially providing a mechanistic explanation for a previously observed preference for waste clearance to occur during N3-like conditions.

Authors' response: We are unsure why (if that is correctly understood) the reviewer focuses purely on the results re. the observations in N3 sleep; the study has a number of additional outcomes that align with the interpretations made. However, to make a more clear distinction between our data and discussion regarding their potential implications for waste clearance, we have modified as shown below:
11Lines 418-421:
"Consequently, we here provide support for the hypothesis that humans - alike mice – have a sleep- and slow-wave-dependent influx of CSF into the brain parenchyma, which, by causing an exchange of CSF and interstitial fluid, may be involved in clearing the brain of waste products"

This is a stretch, see above. The simplest explanation is that partial volume effects with CSF explain the GM-WM difference in MREG signal. The statement should be weakened and the (I believe based on physics) much more likely explanation should be discussed.
.
Line 420: "these important findings offer crucial insights into how the vasomotor system in the brain responds to sleep deprivation, potentially serving as a means to mitigate the accumulation of brain waste products"
Authors' response: It is not clear to us what aspect of this statement the reviewer finds problematic, if any. We believe our data justify this speculation.

Apologies, I sent the pre-final version of my review. I was not sure about the logic here. Since cardiac and respiratory band MREG signals do not show the increase with sleep deprivation, do the authors then suggest that they are less relevant for clearance.

Line 422: "Furthermore, we illustrate that as sleep depth increases, brain pulsations induced by respiration and cardiac activity intensify in both grey and white matter. As the brain pulsations likely reflect brain fluid flow, our observation aligns with preclinical evidence of a slow-wave-dependent influx of CSF into the brain parenchyma.

Authors' response: It is unclear which aspect of this statement the reviewer is questioning. Consequently, we have not changed any text.

Apologies, I sent the pre-final version of my review. As above, I take issue with the statement "As the brain pulsations likely reflect brain fluid flow"
