# Supplementary material for: Sleep deprivation and sleep intensity exert distinct effects on cerebral vasomotion and brain pulsations driven by the respiratory and cardiac cycles
Source: PLoS Biol. 2025 Nov 20;23(11):e3003500. doi: 10.1371/journal.pbio.3003500 (PMC12633874; doi:10.1371/journal.pbio.3003500)
Supplement: Attachment — Submitted filename: ResponseToReviewers_PBIOLOGY-D-25-00068R1.docx [file pbio.3003500.s017.docx]

**PLOS Biology ms #PBIOLOGY-D-25-00068R1**

**Response to Reviewers**

**Sleep deprivation promotes cerebrovascular oscillations while respiration- and cardiac-driven brain pulsations escalate with sleep intensity**

**Authors**

Sara Marie Ulv Larsen^1,2,†^, Sebastian Camillo Holst^1,3,†^, Anders Stevnhoved Olsen^1,4^, Brice Ozenne^1,5^, Dorte Bonde Zilstorff^1^, Kristoffer Brendstrup-Brix^1^, Pia Weikop^6^, Simone Pleinert^1^, Vesa Kiviniemi^7,8^, Poul Jørgen Jennum^9,10^, Maiken Nedergaard^2,6,11^, Gitte Moos Knudsen^1,10*^

______________________________________________________________________

July 15, 2025

Dear Dr. Schnell,

Dear editorial board of PLOS Biology,

Thank you for considering our manuscript “Sleep deprivation promotes cerebrovascular oscillations while respiration- and cardiac-driven brain pulsations escalate with sleep intensity” (PBIOLOGY-D-25-00068) for publication in PLOS Biology. We are grateful for the reviewers’ insightful comments to our manuscript. Below, we have addressed each of the comments point-by-point and when appropriate, made changes to our manuscript which we believe has also increased the clarity of our paper. We hope that you will find the revised version of the manuscript appropriate for publication in the PLOS Biology and we look forward to hearing from you.

On behalf of the authors,
Prof. Gitte M. Knudsen

Reviewer comments (black text)

Authors’ response (blue text)

**Reviewer # 1**

This is an interesting paper that aims at investigating the levels of various types of pulsatile brain fluid (and presumably brain tiss2ue) movement across sleep stages. This is motivated by their potential relevance for brain waste clearance, which an increasing number of studies suggest may be sleep-dependent, with particular contribution from slow-wave (SW) sleep or N3 sleep. Mechanistically, this clearance may be aided by CSF fluid movement, which previous studies have shown to result from various types of vascular pulsations. These include passive (barogenic) pulsations with cardiac and pulmonary cycles, and (slower) active (vasogenic) pulsations from brief changes in neuronal activity or changes in intravascular CO2 from autonomic instabilities. These slower pulsations are referred to as LFO’s by the authors. Previous work has also shown that LFOs are stronger outside N3 and thus do not seem to explain previous experimental findings of increased tracer clearance with N3-like conditions (when SW density is highest). Here it is shown among other things, that N3 like conditions may have stronger barogenic pulsations. This would then suggest that N3-preferential clearance may be aided by these pulsations, rather than LFOs investigated previously. Although no causal relationship between barogenic vascular pulsations and waste clearance is demonstrated, or a mechanistic explanation of why these pulsations would be stronger in N3 sleep, the work provides intriguing information about the dependence of the strength of these pulsations on sleep stage. Most of my comments relate to methodological and interpretational aspects and discussion of existing literature. I believe addressing these comments would strengthen the work.

Authors’ response: We thank the reviewer for the thorough read-through and thoughtful assessment of our manuscript. The reviewer’s comments and suggestions for improving the clarity and interpretation of our findings have been instrumental for the revision. We have carefully considered all points raised and revised the manuscript accordingly, as detailed below.

1. It is not clear why cardiac and respiratory pulsations would be stronger during N3 sleep compared to other states. For example, the cardiac cycle becomes more regular and slower during N3. Would increased pulsation power during N3 then mean that each pulse has stronger effects? Is it possible that some of the pulsation power during other states is missed due to it being outside the integration window? Stabilizing of cardiac and respiratory rate are well known properties of N3 sleep. This is partly because N3 is less punctuated with micro arousals. For example, cardiac rate changes during microarousals (which are most prevalent outside N3) can easily lead to rate increases of 0.2Hz, possibly moving energy outside the window. This can and should be tested by performing the same analysis that is performed on the MREG data to the peripheral cardiac signal. Ditto for the respiratory pulsations.

Authors’ response:

Thank you for this insightful comment.

The reviewer asks whether our observations could be confounded by respiratory and cardiac differences between N3 sleep and other sleep states. We believe this is unlikely, as brain pulsation power in the respiratory or cardiac frequency bands was computed for each 30-second MREG epoch based on the simultaneously recorded respiration- and heart rate signals (see lines 622-637). That is, if heart or respiration rates changed in an individual during e.g. N2 sleep, the frequency window for assessing MREG spectral power was shifted accordingly. We understand if this critical information can be missed, due to the journal requirements where the method follows results. For that reason, we have now also added the information to Results:

Lines 209–2011:
*“.. MREG spectral power within the respiration and cardiac frequency bands was individually tailored for each 30-sec epoch based on simultaneously recorded respiration- and heart rate signals (see Methods)”.*

Moreover, as also described in the manuscript, we included epoch-specific respiration- and heart-rates as fixed effects in our linear mixed models to account for both the 1/F effect and potential influences of changing cardiorespiratory rates on brain pulsation power.

We have now added the following sentence to the model description:

Lines 675–676:

*“To account for vigilance-state related changes in peripheral cardiorespiratory signals as well as the impact of..”*

As per the reviewer’s suggestion, we have now applied the same analysis used on the MREG data to the peripheral cardiac and respiratory signals. When we assess the effect of sleep depth on respiration and heart rates, we find no change in respiration rate and only a slight increase in heart rate from N2 to N3 sleep (+1.6%, p = 0.01; Table S5). This finding is now added to the Results:

Lines 150-152:
*“…while the heart rate increased slightly during N3 vs. N2 sleep (1.6%, p = 0.01) with no change in respiration rate (S5 Table).”*

That is, in this data set we did not see any major differences in heart rate between N2 and N3 sleep. We propose that this could be due to the volunteers being sleep deprived which may be associated with higher sympathetic nervous system activity and increased cardiovascular reactivity (Bigalke et al., 2021. DOI: <https://doi.org/10.1096/fasebj.2021.35.S1.02387>).

2. The differences in power of cardiac and respiratory pulsations between conditions are very small. For example, in Fig.4D, the difference appears to about 30% and 60% respectively between N3 and N2. Considering N2 duration typically is about twice that of N3 during overnight sleep, it is hard to see why N3 pulsations would be more beneficial for clearance than N2 pulsations. This is even harder when not using power of square root of power, which may be a more accurate gauge of pulsation amplitude. This needs more explanation/discussion.

Authors’ response

While we follow the logic of the reviewer re. the duration of N2 vs N3, we also think it is difficult to quantitatively assess the association between brain pulsation power and brain clearance, and that includes statements re. the expectations about what to expect from changes in power. What we do find, and for the first time in humans, is that N3 sleep is associated with higher brain pulsation power than N2. To the extent this comparison is possible, the magnitude of change in brain pulsations parallels findings of Xie et al. of a 60% expansion in interstitial space volume and a 90% increase in CSF tracer inflow between wakefulness and natural sleep in mice.

We have added the following to the Discussion in lines 399-402:

***“****Notably, when participants fell asleep and entered NREM sleep, the strength of respiration- and cardiac-driven brain pulsations increased by 73% and 35%, which can cautiously be compared to the ~60% increase in interstitial space volume reported by Xie el al. in naturally sleeping rodents [8].”*

And lines 421-425:

“.. *However, as the quantitative impact of these stronger pulsations in N3 versus in N2 sleep is difficult to assess directly with our study design, it should be mentioned that the duration of N2 in a typical night’s sleep is about twice as long as that of N3 sleep, potentially leaving more time for brain clearance to take place. This leaves open the question whether the cumulative time spent in NREM sleep or the total amount of sleep slow waves is more important for glymphatic function”*.

**3. Referencing is frequently inaccurate and should be corrected:**

**Line 74:** “Emerging preclinical evidence indicates that slow waves also play an important role in brain fluid dynamics (7)”. Reference 7 is a review paper and it is not clear what evidence in this paper supports this statement. Please cite direct evidence.

Authors’ response:

We would appreciate if the reviewer could point us to a more recent comprehensive and complete review on brain fluid dynamics, glymphatic regulation, and the proposed role of slow waves to introduce readers to the topic in the Introduction. Also, the review does not stand alone but is followed by citations of original research (Refs. 8–10).

Also, reference 7 cites the Fultz Science paper, but does not cite Picchioni (reference 46 in current manuscript), who shows, in contrast with Fultz, that a dominant contribution to CSF flow during N2 is autonomic, not neural activity. Autonomic activity (CO2 changes, sympathetic vasoconstriction) is shown to be a major contributor to LFOs studied here and this is not mentioned at all in the entire manuscript, nor in Fultz. This should be corrected to provide an evenhanded account.

Authors’ response:
Thank you for pointing this out.

We have now added the Picchioni reference (reference 41 in revised manuscript) to the Introduction line 106:

*“Additionally, drowsiness and autonomic arousal appear to enhance LFOs in wakefulness [40, 41], ..”*

The following statement has also been revised to clarify that NREM sleep promotes CSF influx to the brain, without implying that this process occurs exclusively during sleep:

Lines 67-69: *“NREM sleep enhances the flow of cerebrospinal fluid (CSF) from perivascular spaces into and through the brain parenchyma, which in turn causes an increase in interstitial… space volume and an exchange of CSF and interstitial fluid [8–10].”*

In the Discussion (lines 382-387), we have also expanded our discussion of the potential contribution of autonomic arousal to the effect of sleep deprivation on LFOs:

*“Lastly, because autonomic arousal has been proposed as a potential mechanism for LFO regulation [41], we examined markers of sympathetic activity between well-rested and sleep-deprived wakefulness. However, in our data, blood pressure, heart rate and respiration rate did not differ between the two conditions, indicating that factors beyond both neural slow waves and autonomic arousal likely contribute to the increase in awake LFOs following sleep-deprivation.”*

**Line 76:** Reference 10 shows change in interstitial ion concentration (not interstitial space) during sleep.

Authors’ response: We would prefer to keep reference 10 in the manuscript for the following reason: Ding et al (Ref 10) investigate the relationship between sleep-wake states and changes in interstitial ion concentration but also demonstrate that changing the awake state to that of the sleep state (or vice versa) via administration of awake- or sleep-inducing artificial CSF, is associated with the expected increase in EEG delta power and with an associated increase in interstitial space volume. This paper thus presents an important piece of evidece that vigilance state is associated with interstitial space volume changes (see Fig 5B, C and D in Ding et al., 2016 paper).

**Line 77:** It should be made clear here that reference 9 refers to anesthesia, where slow waves may be of different nature. Also, Reference 11 shows increased CSF tracer infusion with both NREM and REM slow electrical activities. These are quite different, with the latter not typically called EEG slow waves

Authors’ response: We agree and have now added the information that the mice in [9] were anaesthetized. Additionally, Reference 11 has been removed from this statement, but has been retained it in the Discussion section.

Lines 69-70:

*“..This influx of CSF strongly correlates with EEG slow waves in anaesthetized mice [9] and facilitates the …. “*

**Line 81:** “Respiratory and cardiac rhythms generate pressure waves that physically propel CSF into and through the parenchyma [15,16]”. Reference 15 does not show influx into or through the parenchyma. Reference 16 shows influx into the spinal cord (not “through”), and contradicts 15 in that respiratory, not cardiac effects dominate.

Authors’ response: We acknowledge that the current statements may be somewhat imprecise. To address this, we have revised the text and incorporated three additional references to provide stronger support for the statements:

Lines 73-78: *“While sleep overall promotes brain fluid flow, it is believed that this flow is driven by an interplay of low frequency cerebrovascular oscillations, respiration, and the cardiac cycle. Respiratory in- and exhalations, as well as the heartbeat, generate pressure waves that propagate through the brain, physically propelling CSF into and through CSF spaces and the brain parenchyma [14-18]. In parallel, intrinsic cerebrovascular oscillations, also referred to as vasomotion, facilitate CSF-flow along periarterial spaces and into the brain [19,20].”*

**Line 97:** “intracranial pressure …. are also influenced by sleep stage [33,34]. These references talk about REM and N1/N2 having the strongest effects. How does this compare to the other references? More generally, some of the references talk about N3 like conditions being more important for clearance, and some showing evidence for other stages to have more importance. It would be helpful to clearly spell out this dichotomy and show how the current study may clarify it.

Authors’ response: We have intentionally taken a cautious approach in describing the relationship between ICP B-waves and sleep stage as [ref 34 and 35 in revised manuscript] have not (to our knowledge) been replicated, and report varying results depending on whether they assess B-wave frequency, amplitude, wavelength, or morphology. Based on these studies, we believe it is appropriate to say that sleep stages are associated with B-waves but the exact nature and stage-specific characteristics of this relationship remain too uncertain to state definitively in the Introduction.

We do, however, elaborate on these findings in the Discussion (lines 375–377) and have now slightly revised the relevant section of the text in the Introduction.

Lines 92-95:

*“…invasively measured intracranial pressure (ICP) B-waves (0.5 – 2 waves pr min) – which are thought to reflect cerebrovascular oscillations [33] – are also influenced by the sleep-wake cycle and sleep stage [34,35].”*

We agree with the broader point that the Introduction would benefit from a clearer presentation of the current state of knowledge, the dichotomy in previous findings, and how our study may help reconcile this.

To address this, we have updated the following section of the Introduction:

Lines 96-110:
*“However, understanding how changes in vigilance states and slow wave activity relate to fluid flow in the human brain requires non-invasive methods that allow for natural sleep during continuous measurements of both brain fluid dynamics and EEG. Accelerated brain imaging techniques, such as Magnetic Resonance Encephalography (MREG) [36], have emerged as valuable tools for this purpose. MREG is a functional T2*-weighted imaging method with a temporal resolution on the order of milliseconds, enabling non-invasive assessments of low frequency brain oscillations (LFOs) as well as brain pulsations induced by the respiratory and cardiac cycles – all of which have been proposed to reflect brain fluid motion [16,37]. Previous studies have shown that NREM sleep (N1 and N2) enhances all three types of physiological brain oscillations [38], with sleep effects involving larger brain regions in N2 than in N1 sleep [39]. Additionally, drowsiness and autonomic arousal appear to enhance LFOs during wakefulness [40,41], while sleep deprivation specifically increases LFOs in N1 sleep [39]. Although the influence of circadian rhythm and sleep propensity on these findings remain unclear, they suggest that both NREM sleep, sleep depth and wake-related factors modulate MR-detected physiological brain pulsations, with potentially distinct effects on LFOs and cardiorespiratory brain pulsations.”*

**Line 358:** “There were also no significant changes in blood pressure, heart and respiration rates in sleep-deprived wakefulness compared to rested wakefulness. That is, we do not find evidence that autonomic arousal alone explains the observed increases in LFOs, as proposed by Picchioni and colleagues [46].” First, the autonomic arousals reported by Picchioni are accompanied my momentary changes in respiratory flow rate, which are a major part the LFOs. Picchioni did not say anything about average heart/respiratory rates or blood pressure. The authors did not investigate this so should not make this statement. Further, Picchioni does not propose a contribution from autonomic arousal alone! They state that their findings suggest that: ”CSF pulsations do not require slow waves and in part may result from autonomic arousal or autonomic instability”. See their Figure S1 demonstrating that, during sleep, a combination of neural and autonomic effects contribute, with the latter dominating during N2.

Authors’ response: We thank the reviewer for this comment.

We can see that the text could be interpreted as suggesting that Picchioni et al. claim that autonomic arousal alone drives LFOs. Our intention was to acknowledge their findings and to speculate whether the influence of autonomic arousal on LFOs could help explain the increase we observed following sleep deprivation.

We have now rewritten the sentence to:

Lines 382-387:

*“Lastly, because autonomic arousal has been proposed as a potential mechanism for LFO regulation [41], we examined markers of sympathetic activity between well-rested and sleep-deprived wakefulness. However, in our data, blood pressure, heart rate and respiration rate did not differ between the two conditions, indicating that factors beyond both neural slow waves and autonomic arousal likely contribute to the increase in awake LFOs following sleep-deprivation.”*

**Line 316:** References [42,43] should be [43,44]?

Authors’ response: Thank you for noticing this. The references have been corrected.

**Line 488:** References 60 and 61 are not relevant to pulse sequence used here. Ref 62 should be cited instead here.

Authors’ response: Thank you for noticing this. We have replaced [60, 61] with [62].

4. The manuscript would benefit from sharpening up the terminology. At times, imprecise, and confusing language is used: “brain oscillations/pulsations”: What is meant? Surely, the MRI resolution is insufficient to separate fluid (blood, CSF) from tissue. What is the MREG signal sensitive to? Likely not movement of brain tissue itself, whose visualization needs strong encoding gradients. Almost certainly, most of the pulsatility observed in this work comes from CSF, either in ventricular or subarachnoid space. “fluid”. What is meant? Blood or CSF? “cerebrovascular vasomotor“ what is this. Does this include all of CO2, sympathetic, and local neural activity mediated effects? Maybe use “vasomotor” or “vasogenic” effects and make clear they can originate from various mechanisms (maybe by using a reference).

Authors’ response: We thank the reviewer for the constructive feedback. We have now changed the text as detailed below (see A and B).

A) Brain oscillations/pulsations:

The terms “respiration- and cardiac-driven brain pulsations” are aligned with prior studies investigating MREG spectral power, i.e. MR-detected pulsations in the brain, within the respiratory and cardiac frequency ranges.

The term Low frequency brain oscillations (LFOs) denotes MREG spectral power in the 0.012-0.034 Hz frequency range and does not make any presumptions about the specific biological origin of this signal.

Oscillatory signals in the brain < 0.1 Hz are currently referred to by various terms in the literature (e.g., cerebrovascular oscillations, slow oscillations, vasomotion, vasomotor pulsation, vasogenic oscillations, B-waves/pseudo B-waves, etc.). Here, we generally use ‘cerebrovascular oscillations’, but also incorporate some of the other terms into the manuscript to acknowledge this diversity.

However, we recognize that this could deserve a little more specificity and therefore tightened up the language in the manuscript.

Regarding the suggestion to elaborate on LFO mechanisms, we briefly address these in the Introduction (lines 77–81). A more detailed discussion of local mediators falls outside the scope of this study, as they were not directly investigated and compared here.

Lastly, as a common term for the three investigated physiological signals in the brain, we originally used “brain oscillations.” However, reading through the paper again, we acknowledge the reviewer’s point and have revised the manuscript to use individual descriptors where possible and now instead use the terms “physiological brain pulsation” when referring to all signals collectively.

B) MREG signal:

MREG is a functional T2*-weighted ultrafast imaging technique (10 Hz), meaning that the signal obtained every 100 ms is influenced by both the BOLD (Blood-Oxygen-Level-Dependent) signal and water in, for example, CSF and interstitial fluid spaces.

Due to the relatively low spatial resolution of the MREG signal, we agree with the reviewer that the signal in each voxel—both in grey and white matter—originates from blood and water in CSF spaces, the neuropil, and the vasculature.

As such, the measured signal oscillations reflect changes in these components that are synchronized with the respiration, heartbeat, or occur in the low-frequency range. The MREG signal strength is therefore likely influenced by CSF and blood flow, interstitial water/CSF content, and the compliance of brain tissue within the captured voxels. We elaborate on this interpretation of the signal in the Discussion (lines 340–358; see also reviewer response above) and have also added the current interpretation of MREG-detected brain pulsation to the Introduction (line 132).

To clarify the sensitivity of the MREG signal for the reader, we have added the information that MREG is T2*-weighted accelerated MR imaging techniques to both the Introduction (line 100) and the Discussion (line 340) of the manuscript.

**Line 92:** “while light NREM sleep enhances CSF flow into the fourth ventricle”, here and throughout manuscript, distinguish between net flow and pulsatile movement.

Authors’ response: The below sentences have been revised in accordance with the reviewer’s suggestion.

Lines 88-89: *“…light NREM sleep enhances pulsatile movements of CSF in the fourth ventricle”*

Line 353: *“… temporally associated effects on CSF oscillations.”*

Lines 377-378: *“Similarly, both the slow oscillating CSF signal in the fourth ventricle and its..”*

**Line 149:** “respiration” : respiratory rate Throughout manuscript: Clarify that the CSF signal is taken from the ventricles, and that “grey matter” and “white matter” unavoidably contain a mixture of signals due to partial volume effects in light of the coarse resolution of MREG. In fact, the “grey matter” signal is likely dominated by subarachnoid CSF and this may even be the case for white matter. This should be clarified.

Authors’ response:

Thank you for noting the inconsistency in the use of "respiration" versus "respiratory rate." We have now revised the manuscript to ensure that “respiration” is used when the word is a noun, and “respiratory” is used when it is an adjective.

In Methods (lines 563-568), we clarified already how the CSF signal is defined. We have now further elaborated on this in the limitations section:

Lines 450-453:

*“Moreover, given the coarse spatial resolution of MREG, we assessed CSF pulsations in the lateral ventricles to minimize partial volume effects from adjacent tissues. As a result, our data do not allow for examination of CSF pulsations in the fourth ventricle, as has been done by other studies cited here.”*

Regarding the point that grey and white matter masks include interstitial fluid and CSF, this is a central argument supporting our interpretation that the MREG signal reflects water content and/or tissue compliance. We hope this is clearly conveyed in the following revised statement from the Discussion section:

Lines 343-350:

*“Our data support this interpretation for respiration- and cardiac-driven brain pulsations demonstrating that, irrespective of vigilance state, these pulsations are significantly stronger in CSF spaces than in grey and white matter. During rested wakefulness, they are also more pronounced in grey matter compared to white matter. Considering the differences in water and CSF content, as well as tissue viscosity, across these three brain regions [43,44], our results indicate that the relative water content of a given region (or voxel) is closely associated with the strength of the MREG-detected respiration- and cardiac-driven brain pulsations. Together, this suggests that an increase in CSF influx to the brain parenchyma will amplify brain pulsatility.”*

5. Several statements are not well justified.

Abstract: “In summary, our findings indicate that sleep pressure promotes cerebrovascular oscillations, while sleep slow waves synchronise with respiration- and cardiac-driven pulsations in the brain parenchyma”. This synchronization has not been demonstrated so this statement is not warranted.

Authors’ response: We acknowledge that the original statement could be interpreted as implying that EEG slow waves occur simultaneously with respiration- and cardiac-driven brain pulsations, which we do not demonstrate. We have now revised the statement in the abstract:

Lines 55-57:

*“In summary, our findings indicate that sleep pressure promotes cerebrovascular oscillations, while slow-wave-rich sleep amplifies respiration- and cardiac-driven pulsations in the brain parenchyma.”*

**Line 323:** “and that increases in CSF influx to the brain parenchyma will amplify these pulsations” CSF influx into the parenchyma has not been demonstrated, see above.

Authors’ response: In context, this specific sentence appears in the Discussion and reflects our interpretation of what an increase in the strength of MREG-detected pulsations in grey and white matter may indicate.

The sentence has been revised to better convey this interpretation.

Lines 349–350:

*“Together, this suggests that an increase in CSF influx into the brain parenchyma will amplify brain pulsatility.”*

**Line 379:** “the association between sleep slow waves – i.e. deeper sleep and higher delta power – and brain pulsation strength strongly support the recent suggestion that the synchronous neuronal activity of slow-wave sleep drive fluid and solute movement physio-mechanically into and through the brain [11,53]” How would higher delta power increase cardiac and respiratory cycle related pulsations?

Authors’ response: If sleep slow waves – which are more prominent with higher delta power and deeper sleep – increase influx of CSF into the brain parenchyma, we would expect respiration- and cardiac-driven brain pulsations to likewise strengthen as delta power and sleep depth increases. This is what we observe.

We have changed the Discussion to make this clearer:

Lines 395-398:
*“Because the strength of these MREG detected physiological brain pulsations are likely to scale with tissue water content, their enhancement during NREM sleep is compatible with a sleep-dependent increase in CSF influx to the brain parenchyma, which could be explained by an expansion of extracellular space volume, similar to observations made in the mouse brain [8,10].”*

And

Lines 409-413:
“*.. the association between more sleep slow waves – i.e. deeper sleep and higher delta power – and the increase in strength of respiration and cardiac driven brain pulsations is in line with the recent suggestion that the synchronous neuronal activity of slow-wave sleep may physio-mechanically drive fluid and solute movement into and through the brain [51, 52].”*

**Line 387:** “Consequently, we here provide support for the hypothesis that humans - alike mice – have a sleep- and slow-wave dependent exchange of CSF and interstitial fluid, clearing the brain of waste products”. This statement I feel is inaccurate. I think what has been shown is that barogenic pulsations appear strongest during N3, potentially providing a mechanistic explanation for a previously observed preference for waste clearance to occur during N3-like conditions.

Authors’ response: We are unsure why (if that is correctly understood) the reviewer focuses purely on the results re. the observations in N3 sleep; the study has a number of additional outcomes that align with the interpretations made. However, to make a more clear distinction between our data and discussion regarding their potential implications for waste clearance, we have modified as shown below:

Lines 418-421:
*“Consequently, we here provide support for the hypothesis that humans - alike mice – have a sleep- and slow-wave-dependent influx of CSF into the brain parenchyma, which, by causing an exchange of CSF and interstitial fluid, may be involved in clearing the brain of waste products”.*

**Line 420:** “these important findings offer crucial insights into how the vasomotor system in the brain responds to sleep deprivation, potentially serving as a means to mitigate the accumulation of brain waste products”

Authors’ response: It is not clear to us what aspect of this statement the reviewer finds problematic, if any. We believe our data justify this speculation.

**Line 422:** “Furthermore, we illustrate that as sleep depth increases, brain pulsations induced by respiration and cardiac activity intensify in both grey and white matter. As the brain pulsations likely reflect brain fluid flow, our observation aligns with preclinical evidence of a slow-wave-dependent influx of CSF into the brain parenchyma.

Authors’ response: It is unclear which aspect of this statement the reviewer is questioning. Consequently, we have not changed any text.

6. The authors report an intriguing reduction in cardiac-frequency MREG power with NREM sleep versus placebo. Cardiac pulsations are presumably not related to myogenic mechanisms. Could the authors offer an explanation for this?

Authors’ response: If understood correctly, the reviewer is referring to the observed decrease in MREG spectral power within the cardiac band during NREM sleep when comparing carvedilol versus placebo. We agree that cardiac-driven brain pulsations are happening independently of low frequency cerebrovascular oscillations. The reason for this is likely because of the pharmacological profile of carvedilol. Being a nonselective β1-, β2- and α1-adrenergic antagonist, carvedilol has a vasodilatory effect and it decreases heart contractility. As such, we think that this dampening of the pulsation intensity in the cardiac frequency band by carvedilol is due to a suppression of the heartbeat-induced pressure waves propagating through the vasculature and into the brain.

We have clarified our interpretation in lines 435-440:
*“Consistent with this, we observed reductions in blood pressure, plasma norepinephrine and cardiac-driven brain pulsations, alongside the previously discussed suppression of LFOs. Accordingly, we suggest that the carvedilol-associated attenuation of cardiac-driven brain pulsations reflects its negative inotropic effect on cardiac contractability, which in turn dampens the heartbeat-induced pressure waves propagating through the vasculature and into the brain. Further supporting a primarily peripheral effect of carvedilol in our study, we found no..”*

**Reviewer # 2**

This study investigated how the brain oscillations of different frequency ranges, measured by fast fMRI, after sleep deprivation. It focuses on brain pulsations of the three frequency bands: the low-frequency band (0.012 - 0.34 Hz), respiration and cardiac pulsations bands. It was found that the low-frequency oscillations (LFOs) increased during awake periods after sleep deprivation more than during NREM sleep, and LFOs during sleep are correlated with cognitive measures of sleep pressures via psychomotor vigilance test (PVT). In contrast, the brain pulsations of respiratory and cardiac ranges increased during NREM sleep and were correlated with EEG delta power. In addition, the study also showed that carvedilol, an adrenergic antagonist, dampens LFOs. Overall, this is an important and timely study with very interesting findings. Especially, it differentially linked LFO and respiration/cardiac pulsations to sleep pressure and NREM slow wave sleep.

Authors’ response: We thank the reviewer for taking the time to assess our manuscript and appreciate the positive feedback.

My specific questions and comments are listed below.

1. Why were the PVT measures linked to LFOs during sleep (Figure 3) but not during sleep-deprived awake sessions? Since the LFOs were linked to sleep pressure and had the peak amplitude during sleep-deprived awake whereas non-significant increase during sleep (Figure 2), it would make more sense to me to study the LFOs during sleep-deprived awake.

Authors’ response: We did examine whether PVT measures were associated with LFOs during sleep-deprived wakefulness but that was not the case.
The following sentence has been revised to clarify that we examined this association in both rested and sleep-deprived wakefulness:

Lines 181-182: *“..no such correlation during either rested or sleep deprived wakefulness (p_all_ > 0.5).”*

2. It would be great to provid some comparison for the sleep scoring between the rested and sleep-deprived awake conditions. According to the Methods section, 5-min scans with more than 80% time scored as "wake" were identified and used for LFO quantifications. The 80% looks like a high percentage that would guarantee the dominance of wake periods analyzed. However, if the light sleep stages (N1 and N2) are associated with much larger LFOs than wake, which could be the case as shown in Fultz et al., Science 2019, their biased presence in the 20% of analyzed data could significantly affects the results, particularly the LFO differences seen between the rested and sleep-deprived awake conditions. Most importantly, I don't actually understand the necessity of using the 5-min scans. According to Table S4, only 12 out of 20 subjects have those scans in the sleep-deprived awake condition. The quantification of spectral power of LFOs shouldn't require the continuation of data. To me, it is completely OK to filter the data in the low-frequency range, cut them into 30-sec segments, concatenate those of the same wake/sleep conditions, and then quantify their spectral power, even the 30 seconds are shorter than the LF range (0.012 - 0.034 Hz).

Authors’ response: We thank the reviewer for the thoughtful comments regarding potential sources of bias in our analysis and for the helpful suggestions on how to address them.

To address the concern regarding the potential N1 or N2 contribution to LFO spectral power in defined wakefulness scans, we have now evaluated the EEG scoring for all 5-minute scans included in the analysis: For scans classified as wakefulness, the average proportion of epochs scored as wakefulness by the two scorers was 98% (rested wakefulness: 98.4%; sleep-deprived wakefulness: 97.7%). None of the remaining 2% of epochs were classified as N1 or N2/N3 sleep but as either artifacts or scorer disagreement (e.g., artifact–N1, artifact–N2, artifact–N3, artifact–REM, or N1–N2). That is, we do not believe that N1 or N2 sleep has made any major contribution to the rested versus sleep deprived wakefulnees difference.

Based on these new analyses, we have added the following to the manuscript in the Results section:

Lines 165-168: *“For the 5 min wakefulness scans included in analysis, an average of 98%* *of the simultaneously recorded EEG was classified as wakefulness, with none of the remaining 2% consistently classified as either N1, N2 or N3 sleep. For sleep scans, 91% was classified as N2 or N3 sleep, 0.02% as wakefulness and the remainder as N1 sleep, artifacts or scorer disagreement.”*

The reviewer asks why we did not use 30-second instead of 5-min scans by applying a low-frequency filter to evaluate LFOs spectral power. This would have been viable if one can assume that the LFO waves are sinus-shaped. However, if as indicated by Fultz et al., 2019, LFOs are be more *pulse*-like with separate pulses occurring ~ 0.07-2 times per min (= at 0.012-0.034 Hz), the suggested framework risks contaminating the LFO-power of neighboring epochs with no LFO activity. As an example, if three 30-sec epochs were scored as wake-sleep-wake and an LFO-pulse occurs in the sleep epoch, filtering the data around the LF-frequencies would explicitly construct a sine-wave with those frequencies. That sine-wave is much slower than the 30s epoch and therefore, LFO-power will "spill over" into neighboring epochs, risking wake-epochs being noted as having high LFO-activity. Moreover, since the wavelength of LFOs are longer than 30-sec, neighboring epochs will be artificially highly correlated, which would need to be accounted for in statistical models, thereby necessitating a more complex statististical model.

Nonetheless, we did conduct a sensitivity analysis using 2-minute periods. In this dataset, all four 30-second EEG epochs (100%) within each 2-minute window were classified as *either* wakefulness or NREM sleep (N2/N3). To ensure that each 2-minute period could include at least two full cycles of the LFOs, we applied a high-pass filter at 0.02 Hz and examined a narrower LFO band (0.02–0.034 Hz) compared to that used in the 5-minute dataset. Spectral analysis and statistical procedures were performed as described in Methods for the 5-minute dataset and corroborate the findings from the 5-min epochs.

Results, lines 183-185:
*“A sensitivity analysis using data from 2-min periods fully classified as either wakefulness of NREM sleep rather than 5-min scans confirmed our main finding, showing a 96% increase in awake LFOs following sleep deprivation (*p *= 0.038; see supplementary materials).”*

Supplementary Materials, lines 188 - 204: ***“Effect of sleep deprivation and NREM sleep on LFOs (0.02–0.034 Hz) in a 2-min MREG dataset***

*We conducted a sensitivity analysis of the effect of sleep and sleep deprivation on LFOs using a 2-minute MREG dataset with stricter vigilance state classification than the primary 5-min dataset. Here, all four 30-second EEG epochs (100%) in each 2-minute interval were required to be classified as either wakefulness or NREM sleep (stages N2 or N3) by the two independent EEG scorers (see classification criteria in Methods). In addition, wakefulness scans had to occur during lights-on periods and sleep scans during lights-off periods, as was also the case for 5-min scans. Due to the shorter time window, data were then high-pass filtered at 0.02 Hz, and LFOs were analysed in a narrower frequency band (0.02–0.034 Hz) than in the 5-min intervals. Otherwise, spectral analysis and statistical procedures were performed as described in Methods for the 5-minute dataset.*

*Analysis of this 2-minute dataset (N_RestedAwake_= 19; N_SleepdeprivedAwake_= 12; N_N2N3_= 16) showed that sleep deprivation was associated with an increase LFO spectral power of 96% (*p *= 0.038), while NREM sleep led to a numerical, but non-significant, decrease in power compared to sleep-deprived wakefulness (*p *= 0.38). These findings confirm the main results observed in the 5-minute dataset.”*

3. Why aren't there any results for N1 sleep stage, which could be critical?

Authors’ response: We agree that including N1 sleep stage would have been very valuable but unfortunately, across all MREG data from the rested and sleep-deprived placebo scans, only 32 epochs (30 seconds each) were classified as N1 sleep and 11 of these were from a single participant. Because of the lack of N1 sleep data, we did not attempt to compare these to N2 and N3 sleep.

We have added this clarification to Methods lines 600-601:
*“N1 sleep was not included in analysis due to a limited number of epochs scored as such (n = 32, with 11 originating from a single participant).”*

4. Should the upper frequency limit for LFOs be 0.034, as shown in Figure 2, rather than 0.34, which appear in several places in the paper?

Authors’ response: We thank the reviewer for catching this typo. The correct upper frequency limit is 0.034 Hz, as shown in Figure 2. This has now been corrected in the manuscript.

5. Are 34 data points (14 for the placebo group whereas 17 carvedilol group) in Figure 3 due to the availability of 5-min N2/N3 sleep data? Again, my comment above applies to here. The power of LFOs can be assessed using 30-sec epochs.

Authors’ response: Yes, that is correct — the 34 data points in Figure 3 reflect the availability of 5-minute N2/N3 sleep data.

6. The associations between the brain pulsations of respiratory and cardiac frequencies and delta power ratio (Figure 4F-G) are largely overlapped with their differences seen between awake and sleep (Figure 4A-D). The associations are largely driven by the differences between data points from awake (blue) and sleep (orange) conditions, which are known to have significantly different delta power. If the purpose of this association analysis was to further emphasize the link between these pulsations with slow wave activity, it should be conducted within sleep condition. In addition, what is the rationale of excluding the sleep-deprived awake from this analysis?

Authors’ response: The purpose of this analysis was indeed to validate the observed effects of NREM sleep and sleep depth on respiration- and cardiac-driven brain pulsations using a quantitative approach that did not rely on manual EEG-based sleep staging. This analysis also alloweded us to include a larger amount of data, as epochs with scorer disagreement as well as N1 sleep was included, which is not the case in the wakefullness vs N2N3 sleep and sleep depth analyses.
To clarify this, we have slightly revised the following text in the manuscript

Lines 252–255:
*“To validate the found effects of NREM sleep and sleep depth on respiration- and cardiac-driven brain pulsations in a quantitative and sleep-scoring independent manner, we quantified EEG delta-power ratio across all 30-sec epochs from well-rested and sleep-deprived placebo scans, irrespective of their vigilance state classification”*

Morever, we agree with the reviewer that the associations seem to be driven by the inherent difference in delta power between wakefulness and sleep. However, this is both expected and central to the analysis: if the scorer-dependent finding of increased MREG power in cardiorespiratory frequency ranges during N2/N3 sleep is valid, a similar pattern should emerge using delta power as a continuous, scorer-independent proxy for sleep and sleep depth (Figure 4F–G).

Sleep-deprived wakefulness was not excluded from the delta power analysis; the colour symbols in Figure 4F–G reflects scored vigilance states and is included solely for visualization, to help the reader see how different states are distributed. These labels were not used in the analysis.

However, we recognise that the figure legend may have caused confusion and have revised it for clarity:

**Legend, lines 245-247:**
“*Colours represent the scored sleep stages (blue: wakefulness; red: NREM sleep stage 2 and 3; green: uncertain scorings) and are included for visualisation only. Sleep stage was not included in analysis*. ..“

7. Negative results were mentioned but not shown in a few places, e.g., lines 408-410 in page 15, lines 356-357 in page 13, lines 217-219 in page 8. It would be more appropriate to provide those data as supplementary materials if they are less important.

**Authors’ response:** We acknowledge the reviewer’s point and have revised the manuscript accordingly. The relevant statements have been updated and additional data included:

Original lines 408-410 (now revised lines 453-458)
*“Regarding LFOs, there is currently no consensus in their appropriate frequency range. We chose a range consistent with B-waves (0.01 – 0.034 Hz), whereas others have applied a broader frequency band (~ 0.01 – 0.1 Hz) [26,38]. In a posthoc analysis, we assessed whether using a 0.01 – 0.1 Hz band would alter our results and found them consistent with the band used in our study (see supplemetary results)”*

The analysis and results have now been added to Supplementary Materials (lines 205-216):

***“Effect of sleep deprivation and NREM sleep on LFOs in a 0.01-0.098 Hz frequency band in the 5-minute MREG dataset***

*To assess the robustness of our main LFO findings, we conducted a sensitivity analysis on the same 5-min dataset used for our primary analyses, applying a broader 0.01–0.098 Hz frequency band instead of the originally chosen 0.01–0.034 Hz band (see S4 Table).*

*We found that sleep deprivation increased LFO spectral power in the 0.01–0.098 Hz band by 146% (p = 0.01), while NREM sleep led to a 101% reduction compared to the preceding period of sleep-deprived wakefulness (p = 0.02).*

*These results are thus consistent with those observed using the narrower B-wave band, but showed stronger effect sizes and lower p-values. We did not pursue additional analyses in this broader range, as a systematic comparison of LFO frequency bands is outside the scope of the present study.”*

Original lines 356-357:

“*Since plasma norepinephrine was borderline increased during sleep deprivation, we also tested for an association between (change in) norepinephrine levels and LFOs measured in sleep deprived wakefulness or sleep, but found none”.*

We have now removed this sentence from the Discussion. Since the submission of the current manuscript, new preclinical data (Hauglund et al., Cell, Feb 2025) has been published suggesting that norepinephrine modulates cerebrovascular oscillations—i.e., LFOs—during sleep.

Given this development, we do not find it sufficient to include these simple correlations between LFOs and norepinephrine levels in the supplementary materials. Instead, we plan to investigate this potential relationship more thoroughly in future work.

Original lines 217-219 (now revised lines 226-228):

*“We did not observe any correlation between PVT performance and spectral power in the respiration and cardiac frequency bands (p_adj_, _all_ > 0.18).*

8. The effect of carvedilol on LFOs are interpreted as compelling evidence suggesting that LFOs "originate" from cerebrovascular oscillations. Is this to imply the non-neural physiological nature of LFOs? Can we really say that? Like traditional fMRI techniques, the MREG measures BOLD effect that is essentially vascular. Even LFOs originate from neural activity, carvedilol is expected to have effects on them by affecting coupled vascular responses. Moreover, there are increasing evidence suggesting that the cerebral vasomotion are associated with low-frequency neural activity. For fMRI LFOs, recent studies also suggested they may take highly organized pattern as waves propagating across cortical hierarchies, and the existence of whole-brain low-frequency brain activity at single-neuron level. Therefore, it is important to clarify the interpretation and implication of the findings regarding carvedilol's effect.

**Authors’ response:** We agree with the reviewer that LFOs – or cerebrovascular oscillations – arise from both neural and non-neural sources.

Because carvedilol is known to inhibit smooth muscle contraction, the pharmacological intervention should affect LFOs regardless of how they are generated – whether via neurovascular coupling, autonomic regulation or autoregulatory responses to changes in e.g. ICP or CO2.

However, while numerous fMRI-BOLD and glymphatic studies indicate that LFOs reflects cerebral vasomotion, to our knowledge, no previous studies have demonstrated this link via pharmacological intervention. Out finding therefore provides an important piece of evidence that MR-detected whole-brain LFOs originate in cerebrovascular oscillations (regardless of their modulator(s)) – and that they may be amenable to pharmacological modulation.

We recognize that this could have been written more clearly and have revised the following text section of the Discussion:

(Lines 353-358):

*“Notably, we discovered that intervention with the adrenergic antagonist carvedilol – which is known to inhibit smooth muscle contraction and thereby potentially decreases cerebral vasomotion [45,46] – was associated with a reduction in LFOs. This observation supports the current understanding that LFOs originate from cerebrovascular oscillations, and offers encouraging evidence that these oscillations may be amenable to pharmacological modulation.”*

9. Should there be "control" frequency bands to show that changes in brain pulsations in the respiratory and cardiac ranges are indeed related to these two physiological processes? One reason I ask about this is that the cardiac frequency range is overlapped with that for slow waves (0.5-4 Hz), which are significantly implicated as discussed in the Discussion section. Thus, it could be important to find out if similar changes are seen in the frequency ranges that are either between the respiratory and cardiac rhythms, i.e., 0.4-0.5 Hz, or higher than typical heart rate at rest, e.g., 2-4 Hz?

**Authors’ response:** We thank the reviewer for this helpful suggestion and agree that a control frequency band is useful for confirming the physiological specificity of the observed changes in brain pulsations.

Based on the reviewer’s suggestion, we have defined a control frequency band with the same width as the cardiac frequency band (11 bins of 0.002 Hz). Specifically, we selected the range of 3.54–3.74 Hz, which falls within the 0.5–4 Hz slow-wave band but lies above typical heart rates and their harmonics (e.g., ~2 Hz and ~3 Hz), thereby minimizing overlap with cardiac-related signals.

We applied the same linear mixed-effects model as used in our primary analyses (but without controlling for respiratory or cardiac rates) to this control frequency band in the same participant sample. This analysis revealed no significant effect of either sleep deprivation (p = 0.685) or NREM sleep (p = 0.257) on MREG spectral power in the 3.54–3.74 Hz range.

This result has been added to the main manuscript

Lines 229–233:
*“Lastly, to confirm that effects of vigilance state on brain pulsations in cardiorespiratory frequency ranges are indeed linked to these physiological processes, we assessed MREG spectral power in a control frequency band (3.54 – 3.74 Hz), matched in width to the cardiac band and located above typical heart rates and their harmonics. This analysis revealed no significant effect of sleep deprivation (*p *= 0.685) or NREM sleep stages N2 and N3 (*p *= 0.257).“*

**Reviewer # 3**

Ulv Larsen and colleagues present the results of a sleep study with a nested randomised crossover trial. I was asked to review the paper due to the inclusion of the randomised trial; my particular area is statistics in clinical trials.

Firstly, I would say that this seems like a well-conducted study (if a little hard to follow at times, for a non-specialist), and the analyses appear to have been done quite well. I will have some comments on the statistics (I cannot help myself), but these are mainly to do with aspects of the presentation.

Authors’ response: We are grateful for the reviewer’s careful reading of our work and for the constructive statistical advice. The points raised have prompted us to clarify the analyses and results, and to refine some of the wording.

However, I will start with the randomised crossover trial element of the study. This is a 2x2 crossover trail of carvedilol vs. placebo. The facts that it was double blind, that the randomisation was done by someone not otherwise involved in the trial, and that there was a washout period between treatment phases, makes this a well-designed and conducted trial.

The first place I usually start with any trial is the sample size justification. It took me a while to find it, hidden away at the end of one of the supplements, and I would have put it in the main methods section of the paper. The calculation is reproducible, which is a good sign.

Authors’ response: We acknowledge the reviewer’s opinion but prefer to keep the power analysis in the Supplementary to keep the main text as short as stringent as possible.

However, the primary outcomes of the trial are "MREG spectral power within the LFO, respiration and cardiac frequency bands" (line 603), compared at two time points (when awake, and during NREM sleep; Figure 6), suggesting a total of 6 analyses considered "primary" for the trial. Therefore, whist the study had 85% power to detect differences at p<0.01, there was only 83.9% power at a more robust p<0.00833 (i.e. 0.05/3). Still, that is still quite good power, so the sample size is fine. My point is that none of the between-treatment differences shown in Figure 6 meets either of these significance thresholds. So, it could be argued that these results, whilst suggestive of treatment effects, do not provide "compelling evidence supporting the hypothesis that LFOs originate from cerebrovascular oscillations" (lines 330-331). Perhaps the authors should be a little less enthusiastic about the crossover trial results.

Authors’ response: We agree that correcting for multiple comparisons is important. For this reason, we applied Bonferroni corrections when comparing sleep deprived wakefulness, N2 and N3 sleep, as well as when comparing sleep effects across grey matter, white matter and CSF. For these analyses, we report the adjusted p-values in both text and figures.

However, we respectfully disagree with the notion that corrections are required due to the examination of MREG spectral power across the LFO, respiration and cardiac frequency bands. These bands represent three physiologically distinct phenomena in the brain (cerebrovascular oscillations, respiration-driven brain pulsations and cardiac-driven brain pulsations), which are each governed by separate mechanisms.

Our aim was to specifically investigate the effects of sleep, sleep deprivation and treatment on all three types of brain pulsations. As such, we are not selectively analyzing frequency bands to identify favorable results; rather, we report findings for all three frequency bands separately.

To clarify this point, we have added the following to Methods; in lines 661-663:

*“…were considered the primary outcome measures, reflecting low frequency cerebrovascular oscillations, respiration-driven brain pulsations and cardiac driven brain pulsations, respectively. “*

That said, we acknowledge the reviewer’s concern that our interpretation of the crossover trial results may have appeared overly enthusiastic, given the p-values for the treatment effects. In response to Reviewer 2, we have toned down the interpretation that LFOs originate from cerebrovascular oscillations (please see above).

Finally, to address the difference in alpha levels used in the power calculation versus in the statistical analyses reported throughout the manuscript, we have added the following clarification to the Supplementary File 1:

Lines 181-184:  *“The Cohen’s d found by Xie et al. is based on rodent data and may be too optimistic for our study as variability and measurement error are typically more pronounced in human studies. For that reason, we chose to apply an alpha level of 0.01 in the power calculation instead of the 0.05 level applied in the main statistical analyses.”*

Otherwise, the crossover trial is quite well analysed, though with a rather elaborate mixed effects model. Personally, I would adopt a simpler model and look at the sensitivity of the results to alternative methods or further adjustments, but what has been done is fine. That said, I would be explicit about including a fixed effect for period effects; this may be already be done as a side effect of including random effects for scan sessions, but it is not immediately obvious.

Authors’ response: We thank the reviewer for highlighting the need to describe how we account for period effects (i.e., well-rested scan versus first sleep deprived scan versus second sleep deprived scan) more explicitly. In the original analysis we already controlled for session order by including “scan-day” as a random intercept nested within participant.

To clarify this in the manuscript, we have now renamed “scan day” to “period effect” in the description of the linear mixed model in the Methods section (lines 672-673). For further clarity on model parameters, we have also renamed “sleep opportunity” (lights on versus lights off) to “scan type” (lines 673-674).

Additionally, To verify robustness of our modelling, we have now conducted a sensitivity analysis in which we re-fitted the linear mixed models with “period effect” included as an fixed effect rather than a random effect. The period coefficient was non-significant in all three frequency-bands (p_all_ ≥ 0.68), and the carvedilol–placebo estimates remained essentially unchanged; they were identical for the LFO and cardiac bands, and shifted only trivially in the respiration band (*β* = 0.03 to 0.04; *p* = 0.554 to 0.550).

Based on this, we have added the following to the manuscript:

Methods, Statistical Analyses, lines 681-683:
*“Lastly, in a sensitivity analysis, we included the period effect as a fixed effect rather than a random intercept in the linear mixed model described above.”*

Results lines 312-313:

*“These carvedilol-versus-placebo estimates were unaffected by whether scan-day (period effect) was modelled as a random intercept or an explicit fixed effect (see Methods).“*

Beyond the randomised trial, a lot of analyses are presented looking at changes in different parameters between different time points during the study, and correlations between different measures. There is a tendency to adopt language that implies that the associations observed are causal. Whilst many of these assertions may well be true, given the quite tightly controlled conditions in the study, it does not automatically apply that an association observed in a non-randomised, unblinded study, is a causal association. Any statement of cause and effect is, to some degree, taking a leap from the data to the interpretation, so a little caution is recommended.

Authors’ response: We thank the reviewer for this insightful comment and fully agree on the importance of clearly distinguishing between association and causality.

In response to this feedback, we have carefully reviewed the Results, Discussion and Conclusion sections to identify language that may have inadvertently implied causality in the context of observational findings.

In the Results section, we have revised two statements to remove implied causality. The updated versions are as follows:

Line 169:

*“Sleep deprivation was associated with a 120% increase in awake spectral power..”*

Lines 217-218:

*“Compared to rested wakefulness, spectral power in sleep deprived NREM sleep increased by 150% and 42% in the…“*

In the Discussion section, we believe that interpretation and cautious speculation are appropriate when grounded in data and well-reasoned arguments. That said, we acknowledge the reviewer’s perspective and have taken steps to adopt a more careful language where warranted. Accordingly, we have revised the language throughout the Discussion and Conclusion sections to temper and rephrase several statements.

*See* lines 348, 349-350, 351, 367, 390, 395-396, 411-412 and 467-468

Lastly, we have added the following to the limitations section of the Discussion to highlight that the tightly controlled study may limit the generalisability of our findings:

Lines 448-450:

*“Additionally, the study was conducted under tightly controlled conditions and included only young, healthy individuals confirmed to be good sleepers, which further warrants caution when extrapolating the results to broader populations.“*

We believe these revisions more accurately reflect the inferential strength appropriate for our results and trust they address the reviewer's concerns. We appreciate the opportunity to improve the precision of our manuscript.

The sheer number of analyses done in this study does lay it open to type 1 error, and this could perhaps be recognised as a limitation.

Authors’ response: We thank the reviewer for raising the important point regarding the potential for Type I error given the number of analyses conducted. This is of course a critical consideration, which we now acknowledge in the limitations section of the Discussion, lines 458-459:

*“Finally, while we attempted to appropriately adjust for multiple testing, we acknowledge that the number of analyses increases the overall risk of Type I errors.”*

Finally, and I accept this is a particular bugbear of mine, I have never liked the use of the standard error as a descriptive statistic. When the aim is to illustrate a distribution (of a roughly Normal measure), then mean and standard deviation should be used. When the aim is to illustrate the precision of an estimate, then the estimate (e.g. mean, or mean difference) and a 95% confidence interval is a better option. My own personal feeling is that the use of estimate +/- SE, either in text, or particularly in figures, is a way of making differences appear more striking. However, I know this is common practice away from my own sphere of statistical analysis, so I am not going to insist that the authors change what they have done.

Authors’ response: We appreciate the reviewer’s preference for presenting variability using standard deviations (SD) or confidence intervals rather than standard error of the mean (SEM).

To address this point, we have revised Table 1 to report SD in a separate column rather than using the mean ± SD format. Hopefully, this clarifies the measure used.

Regarding the use of estimated means ± SEM in the figures, we have opted to retain the current format.

Importantly, percent changes and p-values are reported for all results in the text, providing additional context for interpreting the figures. We believe this approach strikes a balance between figure clarity and transparency in reporting.

**Editorial requests requiring changes to the manuscript**

Thank you for stating" Approval was granted by the Danish ethics committee of the Capital Region of Denmark (journal ID: H-16045933) and the study adhered to the principles of Declaration of Helsinki.Written informed consent was obtained from all participants before enrolment in the studies.

Please insert an Ethics Statement at the beginning of your Methods section, under a subheading 'Ethics Statement' and include the details. NOTE: It must include:
1) The name(s) of the Institutional Review Board(s) or Ethics Committee(s)
2) The approval number(s), or a statement that approval was granted by the named board(s) [3) A statement that formal consent was obtained (must state whether verbal/written) OR the reason consent was not obtained (e.g. anonymity). NOTE: If child participants, the statement must declare that formal consent was obtained from the parent/guardian.]

An etichs statement section has now been added to the beginning of Methods.
See lines 479-481

We noticed that you used "data not shown (data not reported) " in the manuscript. We do not allow these references, as the PLOS data access policy requires that all data be either published with the manuscript or made available in a publicly accessible database. Please amend the supplementary material to include the referenced data or remove the references.

The supplementary materials and results have been amended to include all references data. See response to reviewer request on the same matter above.

Currently, [Graphical Abstract] in your submission file inventory does not have an in-text citation. If the figure is no longer to be included as part of the submission, please remove it from the file inventory.

[Graphical Abstract] has been changes to Fig 7 and is now referenced in the Discussion line 330.
