# Supplementary material for: Sleep deprivation and sleep intensity exert distinct effects on cerebral vasomotion and brain pulsations driven by the respiratory and cardiac cycles
Source: PLoS Biol. 2025 Nov 20;23(11):e3003500. doi: 10.1371/journal.pbio.3003500 (PMC12633874; doi:10.1371/journal.pbio.3003500)
Supplement: Attachment — Submitted filename: ResponseToReviewers_PBIOLOGY-D-25-00068R1_2ndRound.docx [file pbio.3003500.s018.docx]

**PLOS Biology ms #PBIOLOGY-D-25-00068R1**

**Response to Reviewers 1. 2^nd^ round of comments.**

**Sleep deprivation promotes cerebral vasomotion while respiration- and cardiac-driven brain pulsations escalate with sleep intensity**

**Authors**

Sara Marie Ulv Larsen^1,2,†^, Sebastian Camillo Holst^1,3,†^, Anders Stevnhoved Olsen^1,4^, Brice Ozenne^1,5^, Dorte Bonde Zilstorff^1^, Kristoffer Brendstrup-Brix^1,2^, Pia Weikop^6^, Simone Pleinert^1^, Vesa Kiviniemi^7,8^, Poul Jørgen Jennum^9,10^, Maiken Nedergaard^2,6,11^, Gitte Moos Knudsen^1,12*^

**Reviewer 1**

The authors have done a commendable job revising the manuscript. While the revision is much improved, I still have lingering issues with terminology and inadequately supported claims. While I believe the results are of significant interest, I think these issues should be addressed before acceptance for publication.

Chiefly, the terminology often remains vague and/or ambivalent and at the same time suggestive, making the work prone to misinterpretation and appear to have unwarranted claims. What the authors measure are MRI signal fluctuations in different frequency bands. The manuscript uses numerous terms to tie these to physiological pulsations and to eventually equate them to movement of interstitial fluid in support of waste clearance.

For example, in the abstract alone, five different terms are used to describe pulsations and several of these are vague/hard to understand (“brain oscillations”, “brain pulsations”, “physiological brain pulsations”, “cerebrovascular oscillations”, “pulsations in the brain parenchyma”). Why not say : “we used rapid MRI to study vascular pulsations originating from cardiac and respiratory cycles as well as from myogenic mechanisms, all of which cause movement of CSF and brain tissue and may have relevance for glymphatic clearance”.

We thank the reviewer for the thorough reading of our manuscript and the helpful suggestions for improvement.

First of all, as per the suggestion of the reviewer, we have changed the terminology and now consistently refer to ‘*brain pulsations’* regardless of whether there is a measurable external validation of the pulse (heart beat and respiratory cycle) or not. That is, ‘*Low frequency brain oscillations (LFOs)’* are now termed ‘*Low frequency brain pulsations (LFPs)’.* We hope that helps the readability.

From the suggested language change in the abstract, we realize that the reviewer perceives all MREG-detected brain pulsations (be it low frequency or cardio- and respiratory-driven pulsations) as elicited by changes in the vasculature. Our data do, however, show that the vasodilator carvedilol has an effect on LFP’s but not on respiration-driven brain pulsations. The project design was created exactly to address this question and it is to us a strong indication that we are in fact looking at vasomotion when it comes to LFPs, whereas strength of cardio-respiratory brain pulsations is primarily indicative of extravascular factors such as CSF and water content and flow in the brain.

To make this distinction clearer and address the reviewer’s concern that the term ‘cerebrovascular oscillations’ is too vague, we have revised the manuscript to consistently use ‘*cerebral vasomotion’* when describing the physiological phenomenon associated with LFPs, as this term is widely used in preclinical literature and in previous MREG studies. We chose vasomotion over alternatives such as myogenic activity for three reasons:

1. It is the most straightforward and neutral term to describe low-frequency vasoconstriction–vasodilation dynamics in the cerebral vasculature, without implying specific underlying (e.g. myogenic) mechanisms.
2. Carvedilol, our intervention, is a known vasodilator, making vasomotion the most consistent descriptor.
3. The term is widely used in both preclinical and MREG studies. In previously published MREG literature, vasomotion is commonly used to describe LFPs (see refs. 17 and 38–40), while in preclinical studies it refers to slow/low-frequency constriction-dilation dynamics of the cerebral vasculature at comparable frequencies to LFPs (e.g., ref. 14 and new ref 23; Hauglund et al., Cell, 2025, DOI: [10.1016/j.cell.2024.11.027](https://doi.org/10.1016/j.cell.2024.11.027)).

We also incorporated the reviewer’s feedback to simplify the language in the abstract, which now refers to pulsations and vasomotion.

Another vague term is “brain fluid flow” (as well as “waste clearing fluid flow”, “brain fluid dynamics”, “glymphatic flow”). What is this? First, fluid could mean CSF, blood, and interstitial (extracellular) fluid. Upon rereading the manuscript, I believe the authors mean the latter. Secondly, the authors need to distinguish between convective or “net” flow and pulsatile flow.

We use the term “brain fluid flow” as a general descriptor for the movement of CSF within the ventricles, subarachnoidal spaces, perivascular spaces, and interstitial spaces. Similarily, “brain fluid dynamics” refer to the temporal and spatial changes in these CSF movements. We now define these terms in the Introduction (lines 68-70). We acknowledge that, in some instances, the term “brain fluid flow” was too broad to clearly discussing our and others’ results in the previous version of the manuscript, and we have revised the language to be more specific where appropriate.

Regarding the term “glymphatic flow”, we define it in the Introduction and have otherwise limited its use to the Discussion, when referring to preclinical studies in which the term is used.

Related to the term “brain fluid flow”, and critical to the claims in the manuscript, the authors seem to interpret part of the MREG signal to reflect interstitial fluid movement. This however does not make sense considering the minuscule pulsatile movements of brain tissue (see the literature on MRI displacement encoding, e.g. Greitz et al Neuroradiology 1992). In tissue, these can only be observed with the use of strong encoding gradients, and even then only in the phase data (not magnitude data). Previous MRI research has applied various types of MRI contrast (including T2* that the authors claim MREG uses. The understanding of this work is that cardiac and respiratory vascular pulsations result mostly in signals at or near the pial surface, and the slower pulsations (myogenic effects from neurovascular control and CO2/sympathetic mechanisms) accentuate areas with high blood volume (grey matter, large veins and their surrounds). At the minimum, they authors should explain how MREG would be able to detect interstitial fluid pulsations, and what are potential pitfalls with this interpretation.

As noted above, we do not make statements about what part of the CSF movement we are measuring with MREG spectral power in cardiorespiratory frequency ranges, as the method is not suited for that purpose.

We believe that what we are measuring is brain pulsations induced by cardio-respiratory cycles, and that the strength of these is influenced by the water/CSF content in the imaged voxels, including both perivascular spaces and the brain parenchyma/interstitial spaces.

To make this clear to the reader, we have added the following to the limitations section in the Discussion, Lines 800-801:

*“Moreover, given the coarse spatial resolution of MREG, we cannot distinguish CSF in perivascular spaces from CSF and water in the brain parenchyma, and further chose to assess CSF space pulsations in the lateral ventricles to minimize….”*

While our data support that MREG power in the LFP frequency range reflects vasomotor activity (and possibly its associated effects on CSF flow), the source of the MREG-detected signal changes in cardiorespiratory frequency bands is likely to originate from vascular as well as extravascular factors, such as water and CSF content and movement in perivascular and interstitial spaces. This interpretation is consistent with that of the MREG developers’ (ref 38) and has also been adopted in other MREG papers (see ref 17, 39–40).
Accordingly, higher MREG spectral power in cardiorespiratory frequency ranges (i.e. stronger respiration- and cardiac-driven brain pulsations) is indicative of increased inflow to and/or a greater amount of CSF and water in perivascular spaces and the brain parenchyma.

We recognise that we may have overemphasized CSF influx into the brain parenchyma in our discussion of the results and have now revised the language to ensure that CSF in perivascular spaces is explicitly mentioned in the manuscript where relevant.

Lastly, to accommodate the reviewers suggested clarification of the MREG signal interpretation, we have revised the Introduction and Discussion:

Introduction, lines 97-105:

*“Accelerated brain imaging techniques, such as Magnetic Resonance Encephalography (MREG) [37], have emerged as valuable tools for this purpose. MREG is a functional T2*-weighted imaging method with millisecond-level temporal resolution, enabling non-invasive assessment of low-frequency brain pulsations (LFPs) as well as brain pulsations in respiratory and cardiac cycle frequency ranges. LFPs are thought to reflect vasomotion, while the strength of respiration- and cardiac-driven brain pulsations has been suggested to reflect both vascular and extravascular factors such as CSF and water content in the brain [17,38]. Accordingly, variability in these pulsations indicates changes in brain fluid flow.”*

Discussion, lines 344-348:

*“The strength of LFPs and respiration- and cardiac driven brain pulsations, as measured with T2*-weighted accelerated imaging techniques, has been suggested to reflect either the magnitude of CSF flow in perivascular spaces and the brain parenchyma [17,38] or the underlying compliance of brain tissue, encompassing factors such as brain CSF and water amount and tissue viscosity [44,45].”*

Detailed comments are interspersed (in red) with the authors’ responses to the previous review below.

Thank you. We have addressed each comment individually below.

To make the read-through easier, we have included only the comments and questions for which Reviewer 1 provided additional feed-back following our original response. Also, note that references have been updated.

An overview of the color-coding is provided below:
*- Reviewer 1’s original comments (black text)
- Authors’ original response (blue text)*

*- Reviewer 1’s feed-back to revisions (red text)*

*- Authors’s 2nd round of response (green text)*

1. It is not clear why cardiac and respiratory pulsations would be stronger during N3 sleep compared to other states. For example, the cardiac cycle becomes more regular and slower during N3. Would increased pulsation power during N3 then mean that each pulse has stronger effects? Is it possible that some of the pulsation power during other states is missed due to it being outside the integration window? Stabilizing of cardiac and respiratory rate are well known properties of N3 sleep. This is partly because N3 is less punctuated with micro arousals. For example, cardiac rate changes during microarousals (which are most prevalent outside N3) can easily lead to rate increases of 0.2Hz, possibly moving energy outside the window. This can and should be tested by performing the same analysis that is performed on the MREG data to the peripheral cardiac signal. Ditto for the respiratory pulsations.

Authors’ response:

Thank you for this insightful comment.

The reviewer asks whether our observations could be confounded by respiratory and cardiac differences between N3 sleep and other sleep states. We believe this is unlikely, as brain pulsation power in the respiratory or cardiac frequency bands was computed for each 30-second MREG epoch based on the simultaneously recorded respiration- and heart rate signals (see lines 622-637). That is, if heart or respiration rates changed in an individual during e.g. N2 sleep, the frequency window for assessing MREG spectral power was shifted accordingly. We understand if this critical information can be missed, due to the journal requirements where the method follows results. For that reason, we have now also added the information to Results:

Lines 209–211:

*“.. MREG spectral power within the respiration and cardiac frequency bands was
individually tailored for each 30-sec epoch based on simultaneously recorded respiration- and heart rate signals (see Methods)”*

Moreover, as also described in the manuscript, we included epoch-specific respiration- and heart-rates as fixed effects in our linear mixed models to account for both the 1/F effect and potential influences of changing cardiorespiratory rates on brain pulsation power.

We have now added the following sentence to the model description:

Lines 675–676:

*“To account for vigilance-state related changes in peripheral cardiorespiratory signals as well as the impact of..*

As per the reviewer’s suggestion, we have now applied the same analysis used on the MREG data to the peripheral cardiac and respiratory signals. When we assess the effect of sleep depth on respiration and heart rates, we find no change in respiration rate and only a slight increase in heart rate from N2 to N3 sleep (+1.6%, *p* = 0.01; Table S5). This finding is now added to the Results:

Lines 150-152:

*“...while the heart rate increased slightly during N3 vs. N2 sleep (1.6%, p =
0.01) with no change in respiration rate (S5 Table).”*

That is, in this data set we did not see any major differences in heart rate between N2 and N3 sleep. We propose that this could be due to the volunteers being sleep deprived which may be associated with higher sympathetic nervous system activity and increased cardiovascular reactivity (Bigalke et al., 2021. DOI: https://doi.org/10.1096/fasebj.2021.35.S1.02387).

What I meant is that outside N3, K-complexes and micro-arousals lead to biphasic changes in heart rate over a brief (<30s interval) periods. This does not need to result in a change of epoch-averaged hear rate. However, associated pulsation energy can be missed as spectral band is broadened (to as much as 0.4 Hz!). To avoid this confound, the authors should perform the suggested analysis on the cardiac signal. This means analyzing the spectral power of the cardiac (e.g. ECG) signal. The analyses the authors refer to do not accomplish this.

We thank the reviewer for clarifying this important point.

To address the concern, we have now first assessed the epoch-wise standard deviations (SD) of heart rate across sleep states. We find that the average SD was 0.115 Hz in N2 sleep and 0.0754 Hz in N3 sleep. While this confirms that heart rate is more variable in N2 compared to N3 sleep, it also indicates that the width of our cardiac frequency bands—defined as the epoch-wise average heart rate ± 0.1 Hz —is sufficient to capture most pulsation energy.

To further test whether the reported increase in MREG spectral power in the cardiac frequency band during N3 sleep could be driven by cardiac pulsation energy falling outside the defined frequency band in N2 sleep due to high variability in heart rate, we also conducted a sensitivity analysis. Specifically, we excluded the five participants (out of 16 with confirmed N2 sleep) who had the highest heart rate SD during N2 sleep (SD range: 0.012–0.018 Hz) and re-reran the sleep depth analysis on the remaining 11 participants. This analysis produces results similar to those reported for the full sample (shown in line 227-229) demonstrating a 44% increase in MREG power in the cardiac frequency band from N2 to N3 sleep (p < 0.001).

Together, these findings have convinced us that the brief (biphasic) changes in heart rate during N2 sleep do not account for the found increase cardiac-driven brain pulsations during N3 sleep in our study.

Regarding the reviewer’s suggestion to analyse the spectral power of the peripheral cardiac signal: We agree that this is a valuable direction for future work (including assessing the effect of metrics such as heart rate variability, blood pressure, and sympathetic tone). However, we consider this beyond the scope of the present study. We hope the analyses presented above sufficiently address the reviewer’s concern.

**3. Referencing is frequently inaccurate and should be corrected:**

**Line 74:** “Emerging preclinical evidence indicates that slow waves also play an important role in brain fluid dynamics (7)”. Reference 7 is a review paper and it is not clear what evidence in this paper supports this statement. Please cite direct evidence.

Authors’ response:

We would appreciate if the reviewer could point us to a more recent comprehensive and complete review on brain fluid dynamics, glymphatic regulation, and the proposed role of slow waves to introduce readers to the topic in the Introduction. Also, the review does not stand alone but is followed by citations of original research (Refs. 8–10).

I read all the four references the authors used to support their statement (refs 7-10), but none of them shows a causal relationship between SWA and brain fluid dynamics. Reference 9 does show a relationship, but it is only correlational. Therefore, this statement is not warranted.

We do not understand this critique, as the statement does not claim causality but only present the background for our study—namely, that preclinical evidence suggests slow waves (among other factors) play a role in brain fluid dynamics.

However, to address the reviewer’s concern, we have slightly revised the sentence in question and replaced “indicates” with “suggests” to soften the wording.

Lines 67–68:
*“Emerging preclinical evidence suggests that slow waves also play an important role in brain fluid dynamics [7]…”.*

Also, reference 7 cites the Fultz Science paper, but does not cite Picchioni (reference 46 in current manuscript), who shows, in contrast with Fultz, that a dominant contribution to CSF flow during N2 is autonomic, not neural activity. Autonomic activity (CO2 changes, sympathetic vasoconstriction) is shown to be a major contributor to LFOs studied here and this is not mentioned at all in the entire manuscript, nor in Fultz. This should be corrected to provide an evenhanded account.

Authors’ response:

Thank you for pointing this out. We have now added the Picchioni reference (reference 41 in revised manuscript) to the Introduction line 106:
 *“Additionally, drowsiness and autonomic arousal appear to enhance LFOs in wakefulness [40, 41], ..”*

Ref 41 shows that autonomic arousal is dominant during N2 (not “drowsiness” or “wakefulness”) so this statement is misleading. Better to state “LFOs are enhanced during drowsiness and light sleep [40,41] an have a dominant contribution from autonomic arousals [41]”. By the way, the first discovery of this was by Fukunaga et al, MRI 24, 2006.

We have revised the sentence, along with the one preceding it, to follow the reviewer’s suggestion.

Lines 105-108:
*“Previous human studies have shown that NREM sleep stages N1 and N2 enhance all three types of brain pulsations [28, 39, 40], with sleep effects involving larger brain regions in N2 than in N1 sleep [40]. Additionally, LFPs increase during drowsiness [41], in N1 sleep following sleep deprivation [40], and with autonomic arousal [42].”*

**Line 81:** “Respiratory and cardiac rhythms generate pressure waves that physically propel CSF into and through the parenchyma [15,16]”. Reference 15 does not show influx into or through the parenchyma. Reference 16 shows influx into the spinal cord (not “through”), and contradicts 15 in that respiratory, not cardiac effects dominate.

Authors’ response: We acknowledge that the current statements may be somewhat imprecise. To address this, we have revised the text and incorporated three additional references to provide stronger support for the statements:

Lines 73-78: *“While sleep overall promotes brain fluid flow, it is believed that
this flow is driven by an interplay of low frequency cerebrovascular oscillations, respiration, and the cardiac cycle. Respiratory in- and exhalations, as well as the heartbeat, generate pressure waves that propagate through the brain, physically propelling CSF into and through CSF spaces and the brain parenchyma [14-18].*

References 14-18 should be separated and placed after each relevant statement. I believe only 15 and 18 show evidence of parenchymal involvement!

Thank you for noticing this.

We have now placed the appropriate references after each relevant statement, as suggested. We also revised the sentence to remove the direct refence to parenchyma, since we agree with the reviewer that [16] and [20] provide evidence of parenchymal involvement, while [19] illustrates directional flow in perivascular spaces, and [17] shows how physiological impulses propagate through the brain (MREG study). Instead, at the beginning of the section, we now mention parenchymal involvement together with the phrase “is believed to”.

See lines 79-81:
*“.. respiratory in- and exhalations [16–18] together with the heartbeat [17,19,20] generate pressure waves that propagate through the brain, physically propelling CSF into and through CSF spaces and the brain [16–20].”*

Note also that the section containing this sentence has been revised to be more specific about CSF-flow mechanisms and terminology, in line with the reviewer’s main comments (lines 75-81).

*In parallel, intrinsic cerebrovascular oscillations, also referred to as vasomotion, facilitate CSF-flow along periarterial spaces and into the brain [19,20].*

Not sure what “*intrinsic cerebrovascular oscillations”* or “vasomotion” refer to. “Vasomotion” is a highly controversial term and originally was used refer to oscillations that originate in arteries. They have been observed in a specific frequency band in isolated arteries or in animals under physiological stress. I am not of aware of any human data that show such oscillations where the origin is in the arteries. Always, there is a potential other explanation (neural activity, or autonomic activity). Similarly, much of what is discussed in 19 and 20 is neurogenic (evoked by neuronal stimuli through neurovascular response) or possibly with an autonomic component. Why not stay away from “vasomotion” and just refer to LFO??

We thank the reviewer for this perspective.

Following the reviewer’s suggestion at the beginning of the document, we have replaced all mentions of *oscillations –* including *cerebrovascular oscillations -* with alternative wording. As argued in our previous response, and based on our experimental design including the vasodilator compound carvedilol, we have support for the notion that LFPs reflect a vasomotion, i.e., dilation and contraction of the cerebral vasculature. Accordingly, we have replaced all instances of ‘cerebrovascular oscillations’ with ‘vasomotion’ or ‘cerebral vasomotion’ throughout the manuscript.

While we acknowledge the reviewer’s point that, e.g., neuronal activity could be an important mechanism impacting LFPs, the term vasomotion broadly describes the concept of vasodilation and vasoconstriction in the cerebral vasculature without inferring the underlying cause. Moreover, use of the term *vasomotion* is consistent with both prior MREG studies (refs. 17, 39-40), preclinical work referenced here (ref. 14), and a recent paper in Cell examining norepinephrine’s effects on vasomotion (Hauglund et al., 2025; new ref 23).

We agree with the reviewer that LFPs/vasomotion may have multiple origins, which we also touch upon in the Introduction. To clarify this further, the statement in question has been revised and we have added the Hauglund et al. reference:

Lines 81-82:
*“Recent discoveries suggest that vasomotion itself is regulated by neural activity, norepinephrine and the sleep-wake cycle [15, 21–23].*

As for the potential involvement of autonomic arousal, this is already included later in the Introduction line 108 and in the Discussion lines 388-392.

4. The manuscript would benefit from sharpening up the terminology. At times, imprecise, and confusing language is used: “brain oscillations/pulsations”: What
is meant? Surely, the MRI resolution is insufficient to separate fluid (blood, CSF) from tissue. What is the MREG signal sensitive to? Likely not movement of brain

tissue itself, whose visualization needs strong encoding gradients. Almost certainly, most of the pulsatility observed in this work comes from CSF, either in ventricular or subarachnoid space. “fluid”. What is meant? Blood or CSF? “cerebrovascular vasomotor“ what is this. Does this include all of CO2, sympathetic, and local neural activity mediated effects? Maybe use “vasomotor”
or “vasogenic” effects and make clear they can originate from various mechanisms (maybe by using a reference).

Authors’ response: We thank the reviewer for the constructive feedback. We have now changed the text as detailed below (see A and B).

A) Brain oscillations/pulsations:

The terms *“respiration- and cardiac-driven brain pulsations”* are aligned with prior studies investigating MREG spectral power, i.e. MR-detected pulsations in the brain, within the respiratory and cardiac frequency ranges.

The term Low frequency brain oscillations (LFOs) denotes MREG spectral power in the 0.012-0.034 Hz frequency range and does not make any presumptions about the specific biological origin of this signal.

Oscillatory signals in the brain < 0.1 Hz are currently referred to by various terms in the literature (e.g., cerebrovascular oscillations, *slow oscillations*, *vasomotion*, *vasomotor pulsation*, *vasogenic oscillations*, *B-waves/pseudo B-waves*, etc.). Here, we generally use ‘cerebrovascular oscillations’, but also incorporate some of the other terms into the manuscript to acknowledge this diversity.
However, we recognize that this could deserve a little more specificity and therefore tightened up the language in the manuscript.

Regarding the suggestion to elaborate on LFO mechanisms, we briefly address these in the Introduction (lines 77–81). A more detailed discussion of local mediators falls outside the scope of this study, as they were not directly investigated and compared here.

Lastly, as a common term for the three investigated physiological signals in the brain, we originally used *“brain oscillations.”* However, reading through the paper again, we acknowledge the reviewer’s point and have revised the manuscript to use individual descriptors where possible and now instead use the terms *“physiological brain pulsation”* when referring to all signals collectively.
B) MREG signal:

MREG is a functional T2*-weighted ultrafast imaging technique (10 Hz), meaning that the signal obtained every 100 ms is influenced by both the BOLD (Blood- Oxygen-Level-Dependent) signal and water in, for example, CSF and interstitial fluid spaces.

See my main comments. There is no evidence, and it is very unlikely that MREG specifically detects interstitial fluid movement. No physical mechanisms is presented to even lend support to this possibility.

We refer the reviewer to our detailed response to this matter provided below his/her main comment at the beginning of the response letter.

In conclusion, we agree with the reviewer that MREG does not specifically detect interstitial fluid movement.

**Line 149:** “respiration” : respiratory rate Throughout manuscript: Clarify that the CSF signal is taken from the ventricles, and that “grey matter” and “white matter” unavoidably contain a mixture of signals due to partial volume effects in light of the coarse resolution of MREG. In fact, the “grey matter” signal is likely dominated by subarachnoid CSF and this may even be the case for white matter. This should be clarified.

Authors’ response:

Thank you for noting the inconsistency in the use of "respiration" versus "respiratory rate." We have now revised the manuscript to ensure that “respiration” is used when the word is a noun, and “respiratory” is used when it is an adjective.

This was not a noun versus adjective issue. The respiratory cycle can affect MREG through either changes in respiratory rate and/or depth. The authors should specify what is meant.

We thank the reviewer for further elaborating on the original comment.

To clarify, we use the term respiration to denote the cyclic process of inhalation and exhalation. Accordingly, respiration-driven brain pulsations refers to brain pulsations temporally aligned with respiratory movements, irrespective of their exact rate or depth.

Respiratory rates were used to define epoch-specific respiratory frequency bands in the MREG spectra and were also included in the statistical models to account for the 1/f effect and variations in respiration rate. Thus, in this study we do not examine how respiration rate itself influences brain pulsations, but rather correct for it.

However, the reviewer is correct (if we understand his/her comment correctly), that we cannot distictly separate effects of respiratory rates from those of tidal volume in our study. That said, tidal vital volume is known to decrease slightly during NREM sleep (<https://doi.org/10.1016/j.jsmc.2012.06.011>), as is also the case for respiration rate in our dataset.

In the following, we have now specified the role of respiratory rate in our study:

Introduction line 101:
*“…pulsations in respiratory and cardiac cycle frequency ranges.”*

Results lines 212-215:
*“..MREG spectral power within the respiration and cardiac frequency bands was individually tailored for each epoch based on simultaneously recorded respiration and heart rate signals, which were also included in the statistical models to account for changes in cardiorespiratory rates (see methods).*

In Methods (lines 563-568), we clarified already how the CSF signal is defined. We have now further elaborated on this in the limitations section:

Lines 450-453:

*“Moreover, given the coarse spatial resolution of MREG, we assessed CSF pulsations in the lateral ventricles to minimize partial volume effects from adjacent tissues. As a result, our data do not allow for examination of CSF pulsations in the fourth ventricle, as has been done by other studies cited here.”* Regarding the point that grey and white matter masks include interstitial fluid and CSF, this is a central argument supporting our interpretation that the MREG signal reflects water content and/or tissue compliance. We hope this is clearly conveyed in the following revised statement from the Discussion section:

Lines 343-350:
*“Our data support this interpretation for respiration- and cardiac-driven brain pulsations demonstrating that, irrespective of vigilance state, these pulsations are significantly stronger in CSF spaces than in grey and white matter. During rested wakefulness, they are also more pronounced in grey matter compared to white matter*

My point was that the “grey matter” mask has more CSF than the “white matter” mask. So if the pulsation energy is mostly in CSF, then based on partial volume effects you expect more MREG signal in CSF versus GM versus WM, irrespective of the tissue water content or compliance or interstitial fluid (“brain fluid”) content. This gets back to the imprecise use of language mentioned above.

Thank you for clarifying this.

We fully agree with the reviewer. As detailed in our response to the main comment at the beginning of the document, we believe that MREG spectral power in the cardiorespiratory frequency ranges is influenced by CSF and water in both perivascular and interstitial spaces.

We hope our revisions regarding the MREG signal and its interpretation throughout the manuscripts have made this clear.

*“Considering the differences in water and CSF content, as well as
tissue viscosity, across these three brain regions [43,44], our results indicate that the relative water content of a given region (or voxel) is closely associated with the strength of the MREG-detected respiration- and cardiac-driven brain pulsations. Together, this suggests that an increase in CSF influx to the brain parenchyma will amplify brain pulsatility.”*

This is a stretch, see above.

The text section has been revised in accordance with our responses above.
See lines 351-356.

5. Several statements are not well justified.

**Line 323:** “and that increases in CSF influx to the brain parenchyma will amplify these pulsations” CSF influx into the parenchyma has not been demonstrated, see above.

Authors’ response: In context, this specific sentence appears in the Discussion and reflects our interpretation of what an increase in the strength of MREG-detected pulsations in grey and white matter may indicate. The sentence has been revised to better convey this interpretation.

Lines 349–350:
*“Together, this suggests that an increase in CSF influx into the brain parenchyma will amplify brain pulsatility.”*

This is a stretch, see above.

The sentence has been revised. Moreover, we would like to note that the sentence appears in the Discussion section, where we believe it appropriate to speculate on what increases in the strength of respiration- and cardiac-driven brain pulsations may indicate with regard to CSF-flow during sleep.

See lines 355-356:

“*Together, this suggests that an increase in CSF influx into perivascular spaces and the brain parenchyma will amplify brain pulsatility.”*

**Line 379:** “the association between sleep slow waves – i.e. deeper sleep and higher delta power – and brain pulsation strength strongly support the recent suggestion that the synchronous neuronal activity of slow-wave sleep drive fluid and solute movement physio-mechanically into and through the brain [11,53]” How would higher delta power increase cardiac and respiratory cycle related pulsations?

Authors’ response: If sleep slow waves – which are more prominent with higher delta power and deeper sleep – increase influx of CSF into the brain parenchyma, we would expect respiration- and cardiac-driven brain pulsations to likewise strengthen as delta power and sleep depth increases. This is what we observe. We have changed the Discussion to make this clearer:

Lines 395-398:
*“Because the strength of these MREG detected physiological brain pulsations are likely to scale with tissue water content, their enhancement during NREM sleep is compatible with a sleep-dependent increase in CSF influx to the brain parenchyma, which could be explained by an expansion of extracellular space volume, similar to observations made in the mouse brain [8,10].”*

This is a stretch, see above.

The statement has now been further revised in accordance with our responses to the reviewer above and at the beginning of the document.

See lines 401-405.

**Line 387:** “Consequently, we here provide support for the hypothesis that humans - alike mice – have a sleep- and slow-wave dependent exchange of CSF and interstitial fluid, clearing the brain of waste products”. This statement I feel is inaccurate. I think what has been shown is that barogenic pulsations appear strongest during N3, potentially providing a mechanistic explanation for a previously observed preference for waste clearance to occur during N3-like conditions.

Authors’ response: We are unsure why (if that is correctly understood) the reviewer focuses purely on the results re. the observations in N3 sleep; the study has a number of additional outcomes that align with the interpretations made. However, to make a more clear distinction between our data and discussion regarding their potential implications for waste clearance, we have modified as shown below:

Lines 418-421:

*“Consequently, we here provide support for the hypothesis that humans - alike mice – have a sleep- and slow-wave-dependent influx of CSF into the brain parenchyma, which, by causing an exchange of CSF and interstitial fluid, may be involved in clearing the brain of waste products”*

This is a stretch, see above. The simplest explanation is that partial volume effects with CSF explain the GM-WM difference in MREG signal. The statement should be weakened and the (I believe based on physics) much more likely explanation should be discussed.

We are unsure which part of this statement the reviewer considers a stretch. In this section, we are not discussing differences between GM and WM signals, but rather the significant increase in MREG spectral power in the cardiorespiratory frequency bands during NREM sleep (correlating with delta power and sleep depth).

Based on our understanding of the MREG signal, together with our results and previous preclinical findings, we respectfully believe it is appropriate to include this interpretation in the Discussion.

If, however, the reviewer’s concern was the absence of an explicit mention of CSF in perivascular spaces, we have revised the text to address this:

Lines 425-426: *… sleep- and slow-wave-dependent influx of CSF into perivascular spaces and the brain parenchyma, which, by causing an exchange of CSF and..”*

**Line 420:** “these important findings offer crucial insights into how the vasomotor system in the brain responds to sleep deprivation, potentially serving as a means to mitigate the accumulation of brain waste products”

Authors’ response: It is not clear to us what aspect of this statement the reviewer finds problematic, if any. We believe our data justify this speculation.

Apologies, I sent the pre-final version of my review. I was not sure about the logic here. Since cardiac and respiratory band MREG signals do not show the increase with sleep deprivation, do the authors then suggest that they are less relevant for clearance.

Thank you for the clarification. The cardiac- and respiration-driven brain pulsations come into play in N2 and N3 sleep, less so in sleep deprivation. In contrast, LFPs/vasomotion seem to be primarily promoted by heightened sleep pressure. However, our data do not allow for quantitative assessment of the individual contributions of brain pulsations, and therefore, we will abstain from attempting to make such a suggestion.

We acknowledge that the distinction could have been clearer in the conclusion and have now revised it accordingly (incl. previously mentioned updates)

Lines 473–490:

*“We show that heightened sleep pressure and slow-wave-rich sleep exert distinct effects on cerebral vasomotion and on the strength of brain pulsations induced by the respiratory and cardiac cycles.*

*Our investigation unveils that following 35 hours of wakefulness, MR-detected LFPs intensify throughout the entire brain, and that objective measures of sleep pressure exhibit a positive correlation with the magnitude of LFPs during sleep. Additionally, our study suggests that pharmacological vasodilation of the cerebral vasculature affects LFPs, providing support for the notion that LFPs originate from vasomotion. Because cerebral vasomotion is thought to be a key driver of CSF flow in the brain, these important findings offer crucial insights into how the vasomotor system responds to sleep deprivation, potentially serving as a means to mitigate the accumulation of brain waste products. Furthermore, we illustrate that during NREM sleep stages N2 and N3, respiration- and cardiac-driven brain pulsations intensify in both grey and white matter, with strength increasing as sleep deepens. As the strength of these brain pulsations likely reflect brain fluid motion and brain water and CSF content, our observations align with preclinical evidence of a slow-wave-dependent influx of CSF into perivascular spaces and the brain parenchyma. These new findings collectively contribute to our understanding of the intricate dynamics of homeostatic sleep mechanisms, vasomotor activity, brain fluid flow and the clearance of waste products from the brain.**”*

**Line 422:** “Furthermore, we illustrate that as sleep depth increases, brain pulsations induced by respiration and cardiac activity intensify in both grey and white matter. As the brain pulsations likely reflect brain fluid flow, our observation aligns with preclinical evidence of a slow-wave-dependent influx of CSF into the brain parenchyma.

Authors’ response: It is unclear which aspect of this statement the reviewer is questioning. Consequently, we have not changed any text.

Apologies, I sent the pre-final version of my review. As above, I take issue with the statement “As the brain pulsations likely reflect brain fluid flow”

Thank you for clarifying.

We have revised this statement in the conclusion to be more specific in meaning, consistent with the other revisions in the manuscript.

See lines 483–488 and the revised conclusion inserted above.
